# Michael addition of *P*-nucleophiles to azoalkenes provides simple access to phosphine oxides bearing an alkylhydrazone moiety

**DOI:** 10.3389/fchem.2023.1177680

**Published:** 2023-04-13

**Authors:** Alexandr O. Kokuev, Alexey Yu. Sukhorukov

**Affiliations:** Laboratory of Organic and Metal-Organic Nitrogen-Oxygen Systems, N. D. Zelinsky Institute of Organic Chemistry, Russian Academy of Sciences, Moscow, Russia

**Keywords:** organophosphorus compounds, phosphine oxides, azoalkenes, Michael addition, hydrazones, reactive intermediates, chelating ligands

## Abstract

β-Hydrazonophosphine oxides are precursors of useful organophosphorus compounds, including phosphorylated *N*-heterocycles, α-aminophosphonates, and vinylphosphonates. In this work, a general transition metal-free synthesis of β-hydrazonophosphine oxides was developed. The method relies on the Michael addition of phosphine oxides R_2_P(O)H to reactive azoalkenes (1,2-diaza-1,3-butadienes), which are generated *in situ* from α-halohydrazones and Hunig’s base. The reaction stereoselectively leads to *Z*-isomers of β-hydrazonophosphine oxides that are stabilized by intramolecular hydrogen bonding. The conversion of the products thus obtained into potential chelating ligands was showcased.

## 1 Introduction

**Scheme 1:**
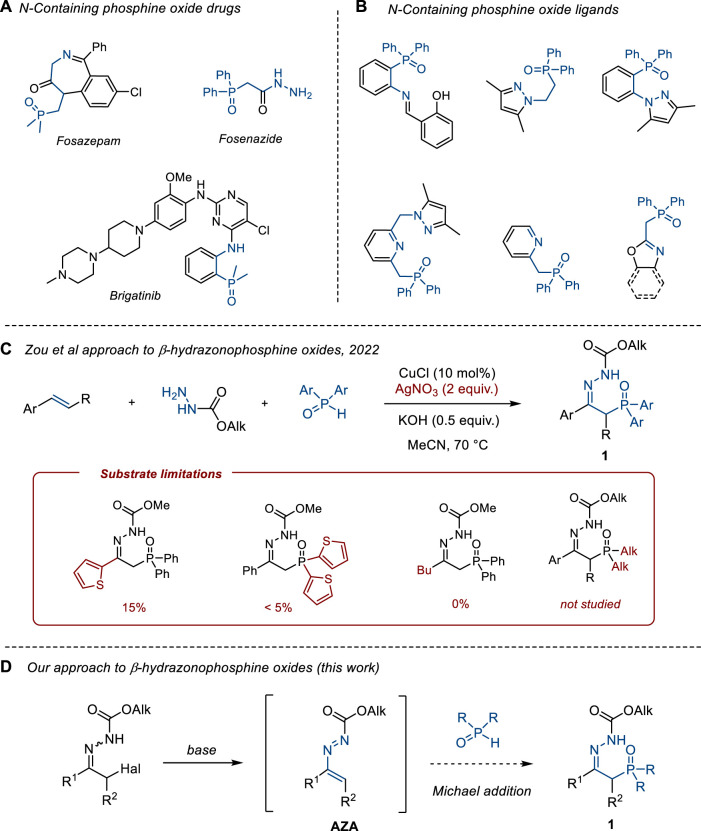
Background of this study. **(A)** *N*-Containing phosphine oxide drugs. **(B)** *N*-Containing phosphine oxide ligands. **(C)** Zou et al approach to hydrazonophosphine oxides **1**. **(D)** Our approach to hydrazonophosphine oxides **1**.

Organophosphorus compounds containing nitrogen are extensively used as pharmaceutical ingredients (Rodriguez and Gallo-Rodriguez, 2019; Yu et al., 2020) and pesticides (Ajiboye et al., 2022). Although the majority of phosphorous-containing drugs correspond to derivatives of phosphoric and phosphonic acids (esters/ amides), phosphine oxides are also applied in clinical practice (Yu et al., 2020). Well-known examples include fosazepam (Nicholson and Wright, 1977), brigatinib (Huang et al., 2016), and fosenazide (Zainkonnikova et al., 1980) (Scheme 1, A). Apart from a biological use, phosphine oxides having additional nitrogen functionality can serve as ligands for transition metal catalysis (Minghetti et al., 1998; Pailloux et al., 2011; Zhang et al., 2012; Aleksanyan et al., 2013; Nyamato et al., 2015; Junges et al., 2019; Enikeeva et al., 2023) and as building blocks for organic synthesis *via* Wittig and HWE-type reactions (Palacios et al., 1994) (Scheme 1B).

Despite the tremendous recent progress in the assembly of the carbon–phosphorus bond *via* transition metal and photoredox catalysis (Tappe et al., 2010; Luo et al., 2017), the development of practical methods for the synthesis of *N*-containing organophosphorus compounds is still in high demand. For instance, this applies to the synthesis of β-hydrazonophosphine oxides **1**, which were previously shown to be convenient precursors of phosphorylated pyrazoles (Palacios et al., 1996; Hassen and Hajjem, 2006), pyrroles (Palacios et al., 1999), pyrrolones (Palacios et al., 2001), pyridinones (Palacios et al., 2001), pyridazines (Palacios et al., 2005b), phosphinyl-substituted ketones (Corbel et al., 1985; Ogundipe et al., 2022b), 1-aza- and 1,2-diaza-1,3-butadienes (Palacios et al., 2006; Hassen et al., 2008), α-aminophosphonates (Palacios et al., 2004; Palacios et al., 2005a), α,β-unsaturated hydrazones (Palacios et al., 1993; Palacios et al., 1994), azaprolines (de los Santos et al., 2008), and vinylphosphonates (He et al., 2020). Recently, Zou et al. developed a Cu-catalyzed hydrazono-phosphinoylation of alkenes with a diarylphosphine oxide approach to β-hydrazonophosphine oxide **1** (Ogundipe et al., 2022a) (Scheme 1C). However, the substrate scope of this method is mostly limited to α-C and P-aryl-substituted products. Heteroaryl- and alkyl-substituted hydrazones **1** are not accessible *via* this reaction, as exemplified in Scheme 1C. Also, the method requires the use of excess silver salt (2 equiv.) as an oxidant, which makes the procedure inapplicable for large-scale synthesis. Similar limitations apply to a related Cu-catalyzed phosphono-hydrazonation of alkynes (Ogundipe et al., 2022b). Other approaches to β-hydrazonophosphine oxide **1** rely on the Arbuzov reaction of α-chlorohydrazones with alkyl phosphinites (Corbel et al., 1985; Delarue-Cochin et al., 2007) and the Michael addition of hydrazines to phosphinyl-substituted allenes (Palacios et al., 1993) or vinyl phosphonium salts (Ovakimyan et al., 2002). These methods require either sophisticated precursors or drastic reaction conditions, thus limiting the substrate scope.

Based on our recent studies on azoalkenes (1,2-diaza-1,3-butadienes, **AZA**) as enolates umpolung synthons (Semakin et al., 2016; Ushakov et al., 2019; Kokuev et al., 2021), we envisioned that this approach can be efficiently used to access hydrazones of type **1** in a transition metal-free fashion. Being highly active Michael acceptors, azoalkenes react with nucleophiles (Grignard reagents, enolates, amines, silyl azides, ylides, etc.) to give α-substituted hydrazones (Attanasi and Caglioti, 1986; Schantl, 1996; Attanasi et al., 2002; Attanasi et al., 2009; Lemos, 2009; Lopes et al., 2018; Ushakov et al., 2022). Surprisingly, the addition of *P*-nucleophiles to azoalkenes remains underexplored (Attanasi et al., 1992; Attanasi et al., 2005; Attanasi et al., 2008), and no examples of the addition of disubstituted phosphine oxides R_2_P(O)H have been reported to the best of our knowledge. Thus, here, we wish to report our studies on the reaction of phosphine oxides R_2_P(O)H with azoalkenes **AZA** that led to the development of a general and practical method for the synthesis of β-hydrazonophosphine oxides **1**.

## 2 Results and discussion

### 2.1 Optimization of reaction conditions

Being of a labile species liable to dimerization and other side reactions, azoalkenes are usually generated *in situ* by dehydrohalogenation of α-halohydrazones (Zhang et al., 2016; Lopes et al., 2018). Due to instability issues, finding suitable conditions for efficient coupling is often challenging and requires special research for each particular nucleophile. The nature of the base was shown to be essential as it controls the stationary concentrations of the **AZA** intermediate and the nucleophile (if the nucleophile is taken in the H-form) (Kokuev et al., 2021). Thus, initially, the reaction of model acetophenone-derived hydrazone **2a** (*Z*-isomer) and diphenylphosphine oxide **3a** in the presence of different bases was studied (Table 1). In these experiments, diphenylphosphine oxide **3a** was initially treated with a base in THF, and then the solution of hydrazone **2a** in THF was slowly added *via* a syringe pump.

**TABLE 1:** Reaction of α-bromohydrazone **2a** with diphenylphosphine oxide **3a**: an optimization study.

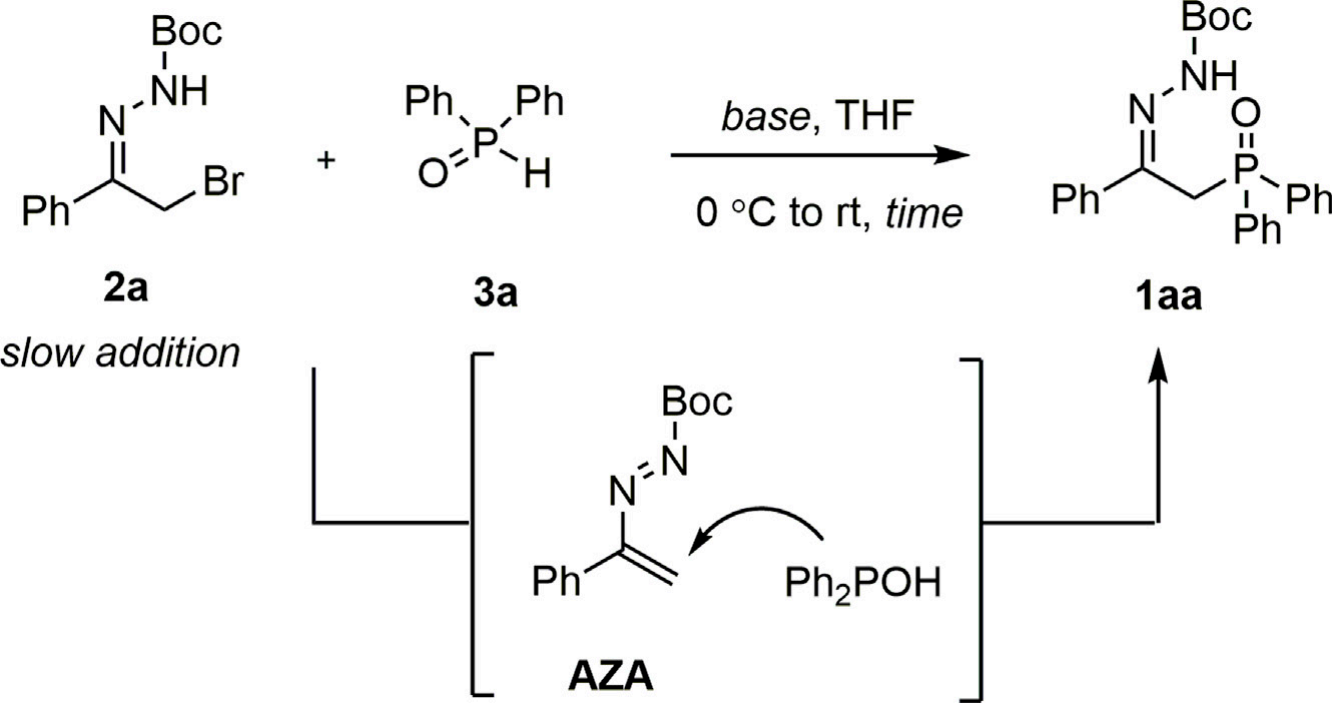

Entry	Base (equiv.)	Time, h	Amount of 3a (equiv.)	Yield of 1aa, %*^a^*
1	None	24	1.5	—
2	K_2_CO_3_ (3.0)	24	1.5	46
3	Cs_2_CO_3_ (3.0)	24	1.5	34
4	^*t*^BuOK (3.0)	24	1.5	27
5	NaH (3.0)	24	1.5	35
6	DBU (3.0)	24	1.5	34
7	Et_3_N (3.0)	24	1.5	54
8	^*i*^Pr_2_NEt (3.0)	24	1.5	84
**9**	^***i***^ **Pr**_**2**_ **NEt (2.5)**	**2**	**1.1**	**88 (86** ***^b^***)

^a^ The yield was determined by ^1^H NMR with an internal standard (CH_2_Br_2_) after aqueous work-up.

^b^ Yield of isolated product **1aa** (column chromatography).

Bold line corresponds to optimized reaction conditions.

In the absence of a base, no desired hydrazone **1aa** was formed (Table 1, entry 1). Alkali metal carbonates are commonly applied as bases to generate azoalkenes from α-halohydrazones (Lopes et al., 2018). Indeed, reactions with potassium and cesium carbonates afforded the desired phosphorylated hydrazone **1aa**, yet only in moderate yields (Table 1, entries 2, 3). The use of stronger bases, such as ^*t*^BuOK and NaH, did not lead to any improvement in the product yield (Table 1, entries 4, 5). We then switched to organic amine bases that are rarely used to generate azoalkenes. While DBU showed poor performance (Table 1, entry 6), triethylamine gave the desired product in 54% yield (Table 1, entry 7). We reasoned that these amines may undergo a competitive Michael addition to the transient **AZA**, leading to side products (Vuillermet et al., 2023). Gratifyingly, the use of a more sterically encumbered Hunig’s base (^*i*^Pr_2_NEt) resulted in the highest yield (84%) of hydrazone **1aa** (Table 1, entry 8). The amount of diphenylphosphine oxide **3a**, the base, and the reaction time could be reduced while maintaining the efficiency of the reaction (Table 1, entry 9).

### 2.2 Substrate scope studies

**Scheme 2:**
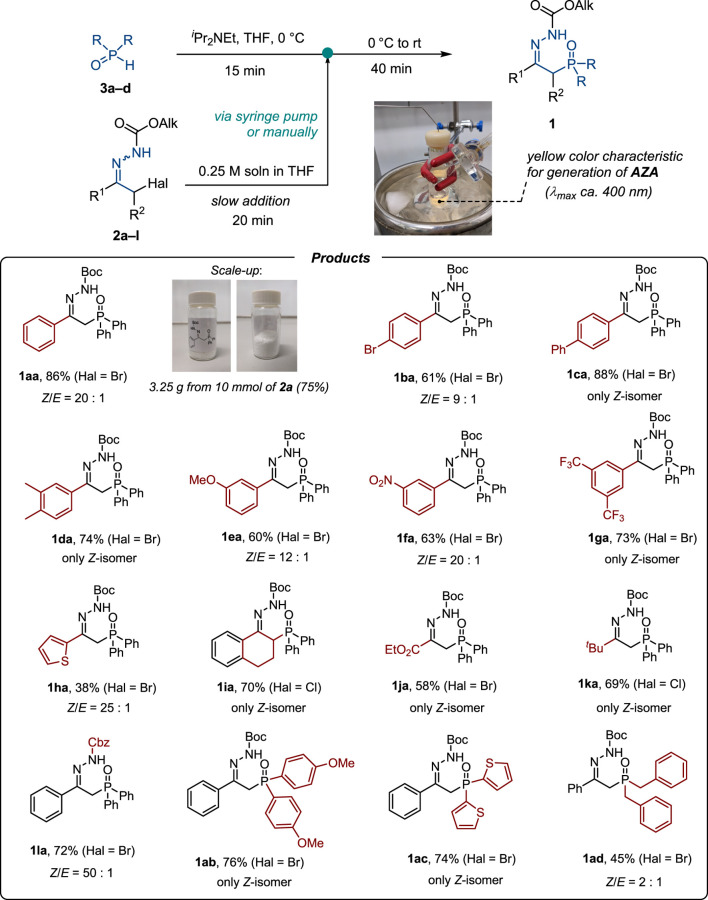
Substrate scope studies. Boc–^*t*^BuOC(O), Cbz–PhCH_2_OC(O). Yields and *Z*/*E* ratios are given for isolated products.

The substrate scope of the reaction was studied under optimized conditions (Scheme 2). α-Bromohydrazones **2a**−**g** derived from substituted acetophenones delivered the desired products **1a**−**g** in the reaction with diphenylphosphine oxide **3a** in high yields. Both electron-rich and electron-poor substituents in the aromatic ring were tolerated in the reaction. It is noteworthy that the challenging thiophenyl-substituted product **1ha** was obtained in 38% yield, which is higher than that in the literature method (Scheme 1C) (Ogundipe et al., 2022a). Tetralone-derived product **1ia** was prepared in 70% yield, demonstrating that β-substituted azoalkenes efficiently enter Michael addition with diphenylphosphine oxide **3a**. The method proved to be applicable to aliphatic substrates, as exemplified by the successful synthesis of products **1ja** and **1ka**. Apart from Boc-hydrazones, the corresponding Cbz-derivatives were shown to enter the process efficiently (as demonstrated by the successful preparation of product **1la**).

The scope of phosphine oxides was also studied. Apart from diphenylphosphine oxide **3a**, di(4-methoxyphenyl)phosphine oxide **3b**, di(2-thienyl)phosphine oxide **3c**, and dibenzylphosphine oxide **3d** afforded the corresponding *P*-substituted hydrazones **1ab**−**1ad** in moderate to high yields (Scheme 2). It is of note that di(2-thienyl)phosphine oxide **3d** was not tolerated in Zou’s method; thus, products of type **1ac** could not be accessed (cf. with Scheme 1C) (Ogundipe et al., 2022a).

The process is scalable, as demonstrated by the synthesis of 3.25 g of product **1aa** without a significant loss of efficiency. It is of note that the required slow addition of a solution of hydrazone **3** could be performed manually without the need for the syringe pump technique.

**Scheme 3:**
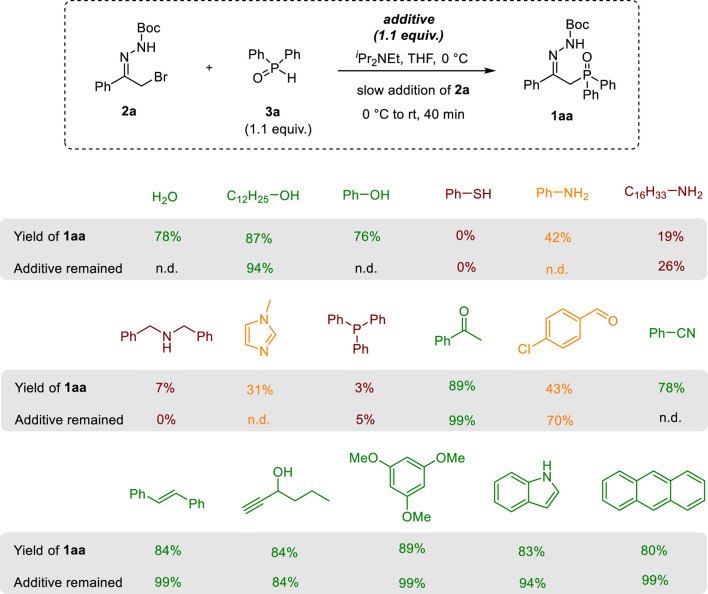
Study of the effect of additives on the reaction efficiency. Yields were determined by ^1^H NMR with an internal standard (trichloroethylene) in the reaction mixtures. n.d.—not determined, Boc–^*t*^BuOC(O). The following color coding is used: green–additives that are tolerated; red–additives inhibiting the reaction to a large extent; orange–additives showing a moderate effect on the product yield.

Studies on the substrate scope show that the developed approach to β-hydrazonophosphine oxides **1** is more general than the methods reported previously. To further explore the scope and limitations of our method, the Glorius additive approach was followed (Collins and Glorius, 2013). In this approach, the functional group tolerance and reaction robustness are assessed by performing the model reaction in the presence of simple and commercially available additives bearing common functionalities. A set of seventeen additives was chosen for this study, including those possessing alkene, alkyne, hydroxyl (alcohol, phenol), thiol, amine (primary, secondary, tertiary, heterocyclic), nitrile, aldehyde, ketone, electron-rich aromatics, and tertiary phosphine moieties (Scheme 3).

Accordingly, the reaction of model bromohydrazone **2a** with diphenylphosphine oxide **3a** was performed in the presence of each of these additives. The yields of the Michael addition product **1aa** and the additive were measured as indicators of the effect of the functional group on the reaction efficiency and tolerance of the functional group under the reaction conditions (the results are summarized in Scheme 3). As seen from this study, water, aliphatic alcohols, and phenols are well tolerated in the reaction. In contrast, thiophenol completely inhibited the formation of the desired product. Amines, including aniline and imidazole, were tolerated poorly. In a similar fashion, triphenylphosphine had a dramatic negative effect on the yield of product **1aa**. In all cases, these additives were consumed under the reaction conditions. These results are logical as thiols, amines, and phosphines are strong nucleophiles that can compete with Ph_2_P(O)H in the Michael addition to azoalkene intermediates (Semakin et al., 2016; Vuillermet et al., 2023). Aromatic ketones and nitriles did not interfere with the reaction, whereas the addition of *p*-chlorobenzaldehyde led to a substantial decrease in the yield of **1aa**. In the latter case, the nucleophilic addition of Ph_2_P(O)H to aldehyde is likely to compete with the Michael addition to the **AZA** intermediate (Zheng et al., 2013).

Since azoalkenes can act as heterodienes in the Diels–Alder reaction (Lopes et al., 2018), additives containing activated π bonds were also studied. We were pleased to find that alkenes, alkynes, electron-rich aromatic rings, and indole were fully tolerated. High yields of product **1aa** and excellent recovery of the additives were observed in all these experiments. Thus, the addition of Ph_2_P(O)H to **AZA** occurs faster than the Diels–Alder reaction and the Michael addition of π-nucleophiles.

### 2.3 Stereochemistry and reaction mechanism

For all the β-hydrazonophosphine oxides **1** obtained, the *Z*-isomer was selectively formed (assignment of the C=N bond configuration was performed on the basis of 2D NOESY and characteristic ^13^C and ^31^P NMR shifts, see Supplementary Material for details). We believe that the *Z*-isomer is thermodynamically more stable than the *E*-isomer due to the intramolecular hydrogen bond between the C(O)N−H and phosphine oxide moieties [no isomerization of (*Z*)-**1aa** was detected upon prolonged heating at 60°C]. The existence of this H-bond is corroborated by a strong downfield NMR shift (ca. 10–11 ppm in CDCl_3_) of the NH hydrogen in (*Z*)-**1** [ca. 8–9 ppm in (*E*)-**1**] and the appearance of ν_N−H_ stretching as a broad band at ca. 3,200 cm^-1^ in the FT-IR spectra. According to DFT calculations at wB97M-D4/def2-TZVP (CPCM: CH_2_Cl_2_) level of theory, the more stable (*Z*)-**1aa** differs from (*E*)-**1aa** by 3.5 kcal/mol (ΔG°). DFT simulation also supports the formation of the intramolecular H-bond (d N(H)…O 2.90 Å, see Supplementary Material for details).

**Scheme 4:**
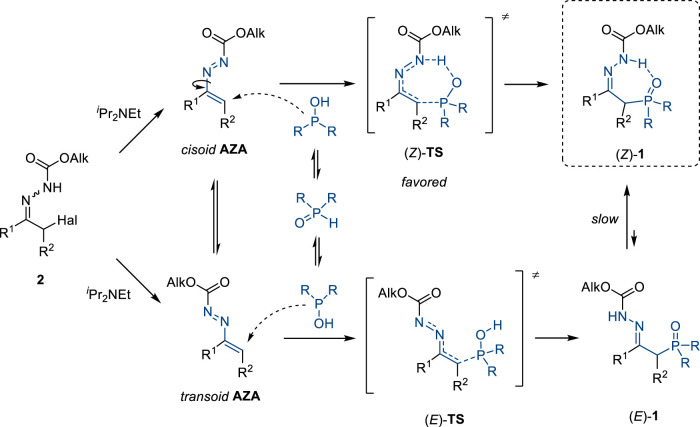
Suggested reaction mechanism.

Yet another possible reason for the preferential formation of *Z*-isomers is of kinetic origin. Both *transoid* and *cisoid* conformations of azoalkenes **AZA** are known to participate in cycloadditions and Michael-type reactions (Clarke et al., 1983; Ferguson et al., 1991). However, the observed *Z*-isomers of products **1** originate from the addition of diphenylphosphine oxide tautomer Ph_2_POH to the *cisoid* conformation of **AZA**. We believe that the corresponding transition state (*Z*)-**TS** is additionally stabilized by the formation of an intramolecular H-bond, as shown in Scheme 4. NMR analysis of crude reaction mixtures resulting from the synthesis of hydrazone **1aa** (before chromatographic separation) showed only trace amounts of the (*E*)-isomer, thus confirming that the (*Z*)-isomer is a kinetic product [*E*/*Z*-isomerization is relatively slow in acyl hydrazones, see Benassi et al. (1982)].

The intermediacy of **AZA** in the reaction was evidenced by the appearance of yellow color (λ_max_ ca. 400 nm), which is characteristic of azo compounds (see Scheme 2 and the Supplementary Material for the UV-Vis spectra). Also, dimer of **AZA** resulting from a self [4 + 2]-cycloaddition reaction was detected by HRMS and ^1^H NMR in the reaction of **2a** with Hunig’s base.

### 2.4 Post-transformations of hydrazone 1aa

**Scheme 5:**
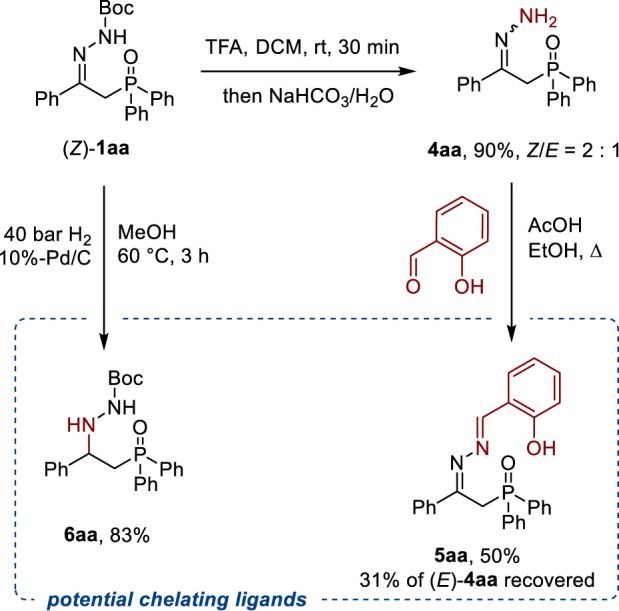
Post-transformations of Boc-protected β-hydrazonophosphine oxide **1aa** and synthesis of potential chelating ligands for transition metals. Boc–^*t*^BuOC(O).

As mentioned in the introduction, the reactivity of β-hydrazonophosphine oxides was extensively explored in previous studies. However, since Boc-protected derivatives **1** obtained by our method were not reported previously, some useful post-transformations were showcased on the model substrate **1aa** (Scheme 5). Deprotection of (*Z*)-**1aa** with TFA gave free hydrazone **4aa** in 90% yield as a 2:1 mixture of *Z*/*E*-isomers. The latter was then brought in the reaction with salicylic aldehyde to give azine **5aa**, which is a structural analog of known chelating phosphine oxide ligands (cf. with Scheme 1) (Hii et al., 1992). Remarkably, only the *Z*-isomer of hydrazone **4aa** underwent condensation with salicylic aldehyde, while the (*E*)-**4aa** isomer remained unreacted and was recovered. Another potential hydrazide-type ligand **6aa** was obtained by hydrogenation of the C=N bond in **1aa** over a Pd/C catalyst. It is of note that the nitrogen–nitrogen bond remained intact under these reductive conditions.

## 3 Materials and methods

For general experimental, instrumental, and computational methods, synthetic procedures, and full compound characterization, see the Supplementary Material.

### 3.1 General procedure for the synthesis of β-hydrazonophosphine oxide 1

To a stirred solution of phosphine oxide **3** (0.275 mmol) in THF (1 mL) was added ^*i*^Pr_2_NEt (0.625 mmol, 108 μL) at 0°C under an argon atmosphere. The mixture was stirred for 15 min at the same temperature. Then, a solution of α-halohydrazone **2** (0.25 mmol) in THF (1 mL) was slowly added in small portions over 20 min (manually or *via* a syringe pump). The mixture was stirred for 10 min at 0°C, and the cooling bath was removed. After stirring for additional 30 min, the mixture was concentrated in a vacuum. The residue was subjected to column chromatography on silica gel to give the corresponding β-hydrazonophosphine oxide **1**.

### 3.2 Gram-scale synthesis of β-hydrazonophosphine oxide 1aa

To a stirred solution of diphenylphosphine oxide **3a** (10.9 mmol, 2.2 g) in THF (40 mL) was added ^*i*^Pr_2_NEt (25 mmol, 4.32 mL) at 0°C. The mixture was stirred for 15 min at the same temperature. Then, a solution of α-bromohydrazone **2a** (10 mmol, 3.13 g) in THF (40 mL) was slowly added in small portions over 20 min. The mixture was stirred for 10 min at 0°C, and the cooling bath was removed. After stirring for additional 30 min, the mixture was concentrated in a vacuum. The residue was dissolved in ethyl acetate (500 mL) and extracted with water. The organic layer was concentrated in a vacuum, and the residue was triturated with methyl *tert*-butyl ether to give 2.92 g of product **1aa**. The mother liquor residue was subjected to column chromatography on silica gel to give an additional 330 mg of product **1aa**. Overall yield: 3.25 g (75%).

### 3.3 *Tert*-butyl 2-[2-(diphenylphosphoryl)-1-phenylethylidene]hydrazinecarboxylate (1aa)

White crystals. Mp 188°C–190°C (AcOEt). Mixture of *Z/E* isomers (ratio 20:1). ^1^H NMR (300 MHz, chloroform-*d*, *Z*-isomer) δ 10.93 (s, 1 H, NH), 7.68 (dd, *J* = 12.0, 8.3, 4 H, *o*-CH_Ph-P_), 7.57 – 7.03 (m, 11 H, Ph), 3.81 (d, *J* = 14.7 Hz, 2 H, CH_2_), 1.56 (s, 9 H, *t-*Bu). ^13^C NMR (76 MHz, DEPT, HMBC, chloroform-*d, Z-*isomer) δ 154.6 (C=O), 141.4 (C=N), 137.9 (d, *J* = 3.0 Hz, C_Ph_), 132.7 (d, *J* = 2.8 Hz, 2 CH_Ph-P_), 131.1 (d, *J* = 10.0 Hz, 4 CH_Ph-P_), 130.5 (d, *J* = 106 Hz, 2 C−P), 128.9 (CH_Ph_), 128.8 (d, *J* = 12.2 Hz, 4 CH_Ph-P_), 128.0 (2 CH_Ph_), 126.5 (2 CH_Ph_), 80.8 (*C*Me_3_), 33.4 (d, *J* = 63.7 Hz, CH_2_P), 28.3 (3 Me). ^31^P NMR (122 MHz, chloroform-*d*, HMBC, *Z*-isomer) δ 33.50. Characteristic 2D NOESY correlations (*Z-*isomer): NH/CH_2_P, NH/*o*-CH_Ph-P._^1^H NMR (300 MHz, chloroform-*d*, *E*-isomer, characteristic signals) δ 8.86 (br s, 1 H, NH), 4.14 (d, *J* = 15.2 Hz, 2 H, CH_2_P). ^31^P NMR (122 MHz, chloroform-*d*, *E*-isomer) δ 29.07. HRMS: *m/z* [M + H]^+^ calcd. for [C_25_H_28_N_2_O_3_P]^+^: 435.1832; found: 435.1825. Anal. Calcd. for C_25_H_27_N_2_O_3_P: C, 69.11%; H, 6.26%; N, 6.45%. Found: C, 68.84%; H, 6.47%; N, 6.18%.

## 4 Conclusion

In conclusion, a convenient transition metal-free method for the synthesis of useful β-hydrazonophosphine oxides from readily available α-halohydrazones was developed. The method features a broad substrate scope, mild reaction conditions, scalability, and stereoselectivity (*Z*-isomers are formed). The reaction tolerates numerous functional groups (alkene, alkyne moieties, electron-rich aromatic rings, indole, aliphatic and aromatic hydroxyl groups, and ketone and nitrile functionalities), as shown using the Glorius additive approach. The reaction mechanism most likely involves the generation of azoalkenes from α-halohydrazones followed by a hydrogen bond-assisted Michael addition of R_2_POH. Conversion of the β-hydrazonophosphine oxides thus obtained into potential chelating ligands was showcased through a chemoselective reduction of the C=N bond and deprotection/azine formation strategies.

## Data Availability

The original contributions presented in the study are included in the article/Supplementary Material; further inquiries can be directed to the corresponding author.
