# Supplementary material for: Michael addition of P-nucleophiles to azoalkenes provides simple access to phosphine oxides bearing an alkylhydrazone moiety
Source: Front Chem. 2023 Apr 13;11:1177680. doi: 10.3389/fchem.2023.1177680 (PMC10133514; doi:10.3389/fchem.2023.1177680)
Supplement: Supplementary file 1 [file DataSheet1.pdf]

Supplementary material

Michael addition of *P*-nucleophiles to azoalkenes provides simple access to phosphine oxides bearing an alkylhydrazone moiety

Alexandr O. Kokuev and Alexey Yu. Sukhorukov*

N. D. Zelinsky Institute of Organic Chemistry, Russian Academy of Sciences,

119991, Leninsky prospect, 47, Moscow, Russian Federation

* Correspondence: Alexey Yu. Sukhorukov, sukhorukov@ioc.ac.ru

Experimental

All reactions were carried out in oven-dried (150 °C) glassware. NMR spectra were recorded at room temperature (if not stated otherwise) with residual solvents peaks as an internal standard. Multiplicities are indicated by s (singlet), d (doublet), t (triplet), q (quartet), m (multiplet), br (broad). HRMS were measured on electrospray ionization (ESI) instrument with a time-of-flight (TOF) detector. Peaks in FTIR-spectra data are reported in cm-1 with the following relative intensities: s (strong), m (medium), w (weak), br (broad), sh (shoulder). Column chromatography was performed using silica gel 40-60 μm 60A with hexane/ethyl acetate mixtures as eluents. Analytical thin-layer chromatography was performed on silica gel plates with QF-254. Visualization was accomplished with UV light and or solution of p-anisaldehyde/H_2SO_4 in ethanol.

Dichloromethane, trimethylamine, and ^{i}Pr_2NEt were distilled from CaH_2; DBU was distilled from CaH_2 under reduced pressure. THF was distilled from $LiAlH_4$. Hexanes, methanol and ethyl acetate (EA) were distilled without drying agents. α-Haloketones, *tert*-butyl carbazate, benzyl carbazate, diphenylphosphine oxide, TFA and 10%-Pd/C were commercial reagents and used as received. Additives (phenol, thiophenol, 1-dodecanol, aniline, 1-hexadecylamine, dibenzylamine, *N*-methyl imidazole, triphenylphosphine, acetophenone, 4-chlorobenzaldehyde, benzonitrile, *trans*-stilbene, hex-1-yn-3-ol, 1,3,5-trimethoxybenzene, indole, and anthracene) were commercial reagents and used as received.

The stereochemical assignment of products was performed on the basis of 2D NOESY (for **1aa**) and known relationships between the configuration of the C=N bond and chemical shifts of α-C atoms in ^{13}C and ^{31}P NMR (see Table S1) as well as by the analogy with previously described hydrazones of type **1**.[1,2,3,4]

Table S1. Characteristic ^{13}C and ^{31}P NMR shifts in products **1**

**Products**	**^{13}C NMR shift of CH_2-P, ppm**		**^{31}P NMR shift of CH_2-P, ppm**	
	(*Z*)-isomer	(*E*)-isomer	(*Z*)-isomer	(*E*)-isomer
**1aa-1ha**, **1la**	33.2–33.9	n.d.	33.1–33.9	26.4–29.1
**1ja**, **1ka**	31.2, 31.6	n.d.	34.2, 32.8	n.d.
**4aa**	31.9	33.7	32.6	27.4

n.d. – not determined due to a low content of (*E*)-isomer

[1] Akacha, A.B.; Barkallah, S.; Zantour, H. *Magn. Reson. Chem.* **1999**, *37*, 916.
[2] Corbel, B.; Medinger, L.; Haelters, J.P.; Sturtz, G. *Synthesis* **1985**, 1048.
[3] Palacios, F.; Aparicio, D.; M. De Los Santos, J. *Tetrahedron* **1994**, *50*, 12727.

Initial α-halohydrazones **2a–l** (Figure S1) were prepared from corresponding α-haloketones and hydrazides according to literature protocols.[4,5,6,7]

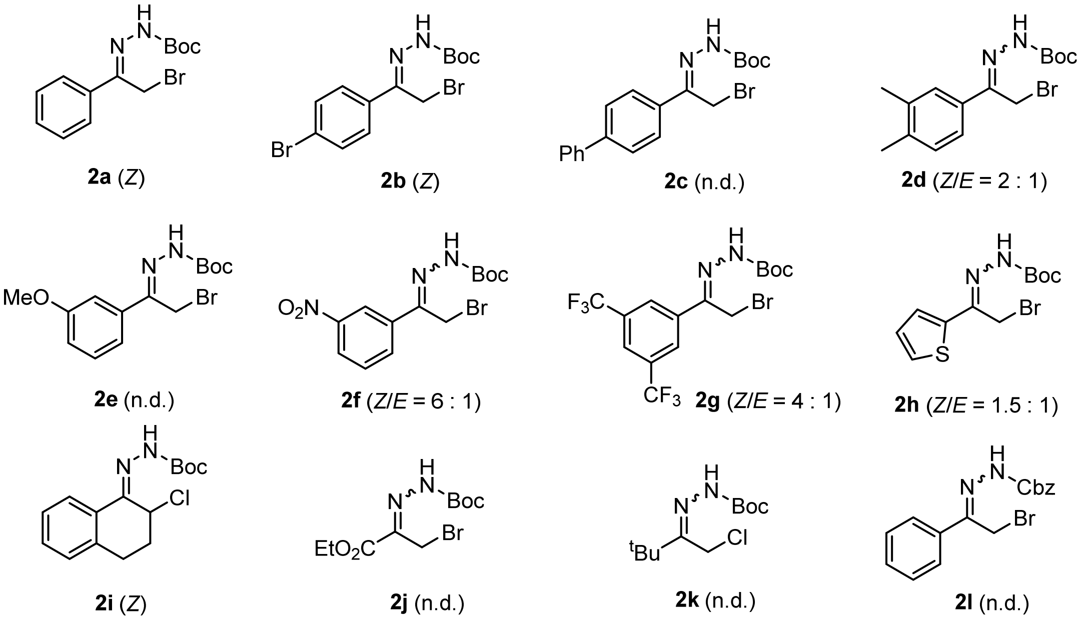

Figure S1. Structures of halohydrazones **2a–l**; n.d. – isomeric composition was not determined.

Initial phosphine oxides **3b**, **3c**, and **3d** (Figure S2) were prepared according to the literature protocol.[8]

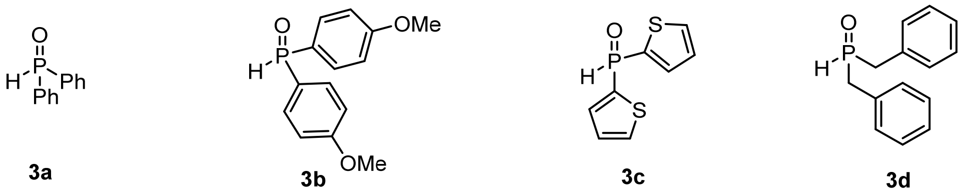

Figure S2. Structures of α-halohydrazones **3a–d**

General procedure for the synthesis of β-hydrazonophosphine oxides 1

To a stirred solution of phosphine oxide **3** (0.275 mmol) in THF (1 ml) was added $^{i}Pr_2NEt$ (0.625 mmol, 108 μl) at 0 °C under an argon atmosphere. The mixture was stirred for 15 minutes at the same temperature. Then, a solution of α-halohydrazone **2** (0.25 mmol) in THF (1 ml) was slowly added in small portions over 20 minutes (manually or via a syringe pump, Figure S3). The mixture was stirred for 10 minutes at 0 °C and the cooling bath was removed. After stirring for additional 30 minutes, the mixture was concentrated in a vacuum. The residue was subjected to column chromatography on silica gel to give the corresponding β-hydrazonophosphine oxide **1**.

[4] Kokuev, A. O.; Ioffe, S. L.; Sukhorukov, A. Yu. *Tetrahedron Lett.*, **2021**, *83*, 153414.
[5] Semakin, A. N.; Kokuev, A. O.; Nelyubina, Y. V.; Sukhorukov, A. Y.; Zhmurov, P. A.; Ioffe, S. L.; Tartakovsky, V. A. *Beilstein J. Org. Chem.* **2016**, *12*, 2471.
[6] Guo, C.; Sahoo, B.; Daniliuc, C. G.; Glorius, F. *J. Am. Chem. Soc.* **2014**, *136*, 17402.
[7] Ramirez, F.; Kirby, A. F. *J. Am. Chem. Soc.* **1953**, *75*, 6026.
[8] Jia, K.; Li, J.; Chen, Y. *Chem. Eur. J.* **2018**, *24*, 3174.

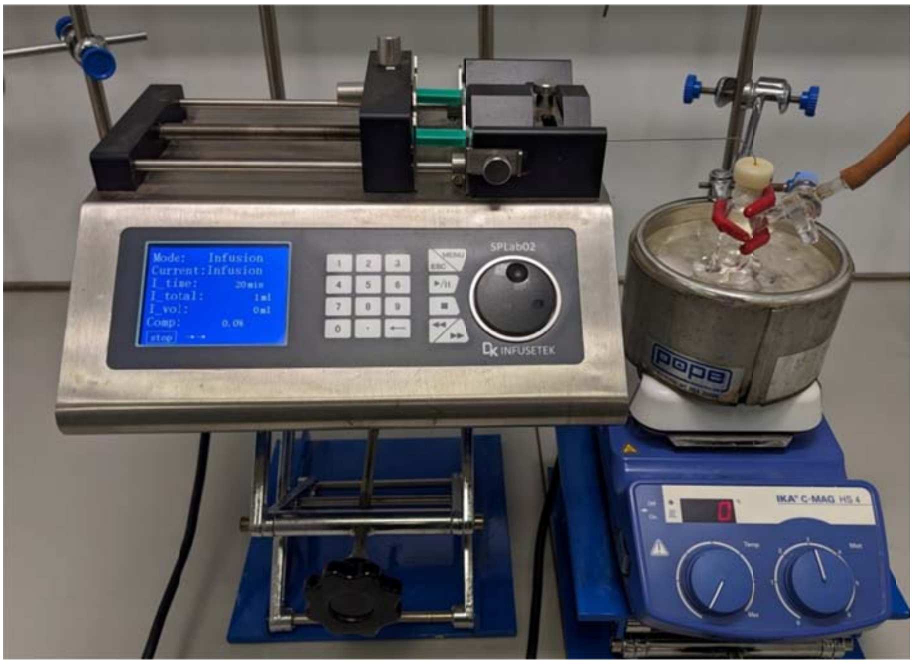

Figure S3. Typical reaction setup for the synthesis of β-hydrazonophosphine oxides **1**

Gram-scale synthesis of β-hydrazonophosphine oxide 1aa

To a stirred solution of diphenylphosphine oxide **3a** (10.9 mmol, 2.2 g) in THF (40 ml) was added $^{i}Pr_2NEt$ (25 mmol, 4.32 ml) at 0 °C. The mixture was stirred for 15 minutes at the same temperature. Then, a solution of α-bromohydrazone **2a** (10 mmol, 3.13 g) in THF (40 ml) was slowly added in small portions over 20 minutes. The mixture was stirred for 10 minutes at 0 °C, and the cooling bath was removed. After stirring for additional 30 minutes, the mixture was concentrated in a vacuum. The residue was dissolved in ethyl acetate (500 ml) and extracted with water. The organic layer was concentrated in a vacuum, and the residue was triturated with methyl *tert*-butyl ether to give 2.92 g of product **1aa**. The mother liquor residue was subjected to column chromatography on silica gel to give additional 330 mg of product **1aa**. Overall yield: 3.25 g (75 %).

Study of the effect of additives on the reaction efficiency (procedure)

To a stirred solution of diphenylphosphine oxide **3a** (56 mg, 0.275 mmol) and the corresponding additive (0.275 mmol) in THF (1 ml) was added $^{i}Pr_2NEt$ (0.625 mmol, 108 μl) at 0 °C under argon atmosphere. The mixture was stirred for 15 minutes at the same temperature. Then, a solution of α-halohydrazone **2a** (0.25 mmol) in THF (1 ml) was slowly added in small portions over 20 minutes. The mixture was stirred for 10 minutes at 0 °C and cooling bath was removed. The mixture was stirred for additional 30 minutes and then concentrated in a vacuum. The yield of product **1aa** and the amount of additive remained were determined by ^{1}H NMR with internal standard (trichloroethylene).

***Tert*-butyl 2-(2-(diphenylphosphoryl)-1-phenylethylidene)hydrazinecarboxylate (1aa)**

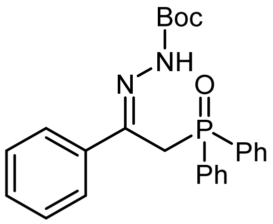

Prepared according to general procedure from 78 mg of bromohydrazone **2a** (0.25 mmol). Yield: 93 mg (86 %). White crystals. Mp 188 – 190 °C (EA).

Mixture of *Z/E* isomers (ratio 20 : 1).

^{1}H NMR (300 MHz, Chloroform-*d*, *Z*-isomer) δ 10.93 (s, 1 H, NH), 7.68 (dd, *J* = 12.0, 8.3, 4 H, *o*-$CH_{Ph\text{-}P}$), 7.57 – 7.03 (m, 11 H, Ph), 3.81 (d, *J* = 14.7 Hz, 2 H, CH_2), 1.56 (s, 9 H, *t*-Bu).

^{13}C NMR (76 MHz, DEPT, HMBC, Chloroform-*d, Z*-isomer) δ 154.6 (C=O), 141.4 (C=N), 137.9 (d, *J* = 3.0 Hz, C_{Ph}), 132.7 (d, *J* = 2.8 Hz, 2 $CH_{Ph\text{-}P}$), 131.1 (d, *J* = 10.0 Hz, 4 $CH_{Ph\text{-}P}$), 130.5 (d, *J* = 106 Hz, 2 C–P), 128.9 (CH_{Ph}), 128.8 (d, *J* = 12.2 Hz, 4 $CH_{Ph\text{-}P}$), 128.0 (2 CH_{Ph}), 126.5 (2 CH_{Ph}), 80.8 (*C*Me_3), 33.4 (d, *J* = 63.7 Hz, CH_2), 28.3 (3 Me).

^{31}P NMR (122 MHz, Chloroform-*d*, HMBC, *Z*-isomer) δ 33.50.

Characteristic 2D NOESY correlations (*Z*-isomer): NH/CH_2P, NH/*o*-$CH_{Ph\text{-}P}$

^{1}H NMR (300 MHz, Chloroform-*d*, *E*-isomer, characteristic signals) δ 8.86 (br s, 1 H, NH), 4.14 (d, *J* = 15.2 Hz, 2 H, CH_2).

^{31}P NMR (122 MHz, Chloroform-*d*, *E*-isomer) δ 29.07.

HRMS: *m/z* $[M+H]^+$ calcd. for $[C_{25}H_{28}N_2O_3P]^+$: 435.1832; found: 435.1825.

Anal. Calcd. for $C_{25}H_{27}N_2O_3P$: C, 69.11 %; H, 6.26 %; N, 6.45 %. Found: C , 68.84 %; H, 6.47 %; N, 6.18 %.

***Tert*-butyl 2-(1-(4-bromophenyl)-2-(diphenylphosphoryl)ethylidene)hydrazinecarboxylate (1ba)**

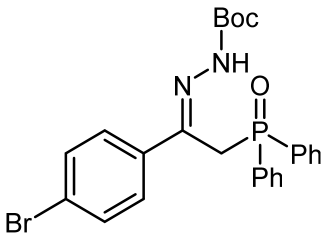

Prepared according to general procedure from 98 mg of bromohydrazone **2b** (0.25 mmol). Yield: 78 mg (61 %). White crystals. Mp 195 – 198 °C (toluene-MeOH).

Mixture of *Z/E* isomers (ratio 9 : 1).

^{1}H NMR (300 MHz, Chloroform-*d*, *Z*-isomer) δ 10.94 (s, 1 H, NH), 7.77 – 7.64 (m, 4 H, Ar), 7.61 – 7.37 (m, 6 H, Ar), 7.30 – 7.11 (m, 4 H, Ar), 3.79 (d, *J* = 14.6 Hz, 2 H, CH_2), 1.57 (s, 9 H, *t*-Bu).

^{13}C NMR (76 MHz, DEPT, Chloroform-*d, Z*-isomer) δ 154.6 (C=O), 143.66 (d, *J* = 9.7 Hz, C=N), 136.8 (d, *J* = 3.1 Hz, C_{Ar}), 132.9 (d, *J* = 2.9 Hz, 2 CH_{Ph}), 131.1 (2 CH_{Ar}), 131.1 (d, *J* = 10.2 Hz, 4 CH_{Ph}), 130.4 (d, *J* = 101.4 Hz, 2 C-P), 129.0 (d, *J* = 12.2 Hz, 4 CH_{Ph}), 128.0 (2 CH_{Ar}), 123.3 (C_{Ar}–Br), 81.2 (*C*Me_3), 33.2 (d, *J* = 63.6 Hz, CH_2), 28.4 (3 Me).

^{31}P NMR (122 MHz, Chloroform-*d*, *Z*-isomer) δ 33.33.

^{1}H NMR (300 MHz, Chloroform-*d*, *E*-isomer, characteristic signals) 4.11 (d, *J* = 15.1 Hz, 2 H, CH_2).

^{31}P NMR (121.49 MHz, Chloroform-*d*, *E*-isomer) δ 26.71.

HRMS: *m/z* $[M+H]^+$ calcd. for $[C_{25}H_{27}BrN_2O_3P]^+$: 513.0937; found: 513.0937.

Tert-butyl 2-(1-([1,1'-biphenyl]-4-yl)-2-(diphenylphosphoryl)ethylidene)hydrazinecarboxylate (1ca)

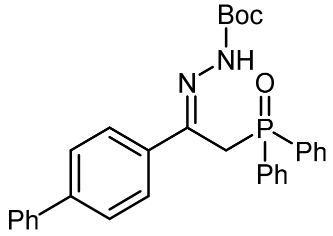

Prepared according to general procedure from 97 mg of bromohydrazone **2c** (0.25 mmol). Yield: 112 mg (88 %). White crystals. Mp 169 – 171 °C (EA).

Single *Z* isomer.

^{1}H NMR (300 MHz, Chloroform-*d*) δ 10.97 (s, 1 H, NH), 7.73 (dd, *J* = 12.0, 6.9 Hz, 4 H, Ar), 7.60 – 7.31 (m, 15 H, Ar), 3.85 (d, *J* = 14.6 Hz, 2 H, CH_2), 1.59 (s, 9 H, *t*-Bu).

^{13}C NMR (76 MHz, DEPT, Chloroform-*d*) δ 154.6 (C=O), 144.5 (d, *J* = 9.3 Hz, C=N), 141.4 and 140.2 (2 C_{Ar}), 136.8 (d, *J* = 3.0 Hz, C_{Ar}), 132.7 (d, *J* = 2.9 Hz, 2 CH_{Ph}), 131.1 (d, *J* = 9.9 Hz, 4 CH_{Ph}), 130.5 (d, *J* = 101.2 Hz, 2 C–P), 128.8 (d, *J* = 12.2 Hz, 4 CH_{Ph}), 128.7 (2 CH_{Ar}), 127.6 (CH_{Ar}), 126.9 (4 CH_{Ar}), 126.5 (2 CH_{Ar}), 80.9 (*C*Me_3), 33.2 (d, *J* = 63.5 Hz, CH_2), 28.4 (3 Me).

^{31}P NMR (122 MHz, Chloroform-*d*) δ 33.52.

HRMS: *m/z* $[M+H]^+$ calcd. for $[C_{31}H_{32}N_2O_3P]^+$: 511.2145; found: 511.2152.

Tert-butyl 2-(1-(3,4-dimethylphenyl)-2-(diphenylphosphoryl)ethylidene)hydrazinecarboxylate (1da)

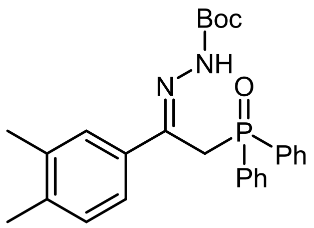

Prepared according to general procedure from 85 mg of bromohydrazone **2d** (0.25 mmol). Yield: 85 mg (74 %). White crystals. Mp 195 – 196 °C (EA).

Single *Z* isomer.

^{1}H NMR (300 MHz, Chloroform-*d*) δ 10.89 (s, 1 H, NH), 7.76 – 7.62 (m, 4 H, Ar), 7.44 (m, 6 H, Ar), 7.13 – 6.77 (m, 3H, Ar), 3.77 (d, *J* = 14.6 Hz, 2 H, CH_2), 2.13 (s, 3H, Me), 2.05 (s, 3H, Me), 1.56 (s, 9 H, *t*-Bu).

^{13}C NMR (76 MHz, DEPT, Chloroform-*d*) δ 154.8 (C=O), 145.1 (d, *J* = 9.9 Hz, C=N), 137.7 (C_{Ar}), 136.2 (C_{Ar}), 135.5 (d, *J* = 3.0 Hz, C_{Ar}), 132.7 (d, *J* = 2.8 Hz, 2 CH_{Ph}), 131.2 (d, *J* = 9.8 Hz, 4 CH_{Ph}), 130.8 (d, *J* = 101.2 Hz, 2 C–P), 129.2 (CH_{Ar}), 128.9 (d, *J* = 12.2 Hz, 4 CH_{Ph}), 127.9 (CH_{Ar}), 123.9 (CH_{Ar}), 33.5 (d, *J* = 63.9 Hz, CH_2), 80.9 (*C*Me_3), 28.5 (3 Me), 19.7 (Me), 19.6 (Me).

^{31}P NMR (122 MHz, Chloroform-*d*) δ 33.51.

HRMS: *m/z* $[M+H]^+$ calcd. for $[C_{27}H_{32}N_2O_3P]^+$: 463.2145; found: 463.2139.

Anal. Calcd. for $C_{27}H_{31}N_2O_3P$: C, 70.11 %; H, 6.76 %; N, 6.06 %. Found: C, 70.17 %; H, 6.25 %; N, 5.87 %.

***Tert*-butyl 2-(2-(diphenylphosphoryl)-1-(3-methoxyphenyl)ethylidene)hydrazinecarboxylate (1ea)**

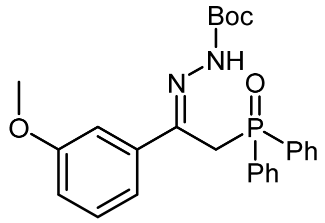

Prepared according to general procedure from 86 mg of bromohydrazone **2e** (0.25 mmol). Yield: 53 mg (60 %). White crystals. Mp 206 – 208 °C (EA).

Mixture of *Z/E* isomers (ratio 12 : 1).

^{1}H NMR (300 MHz, Chloroform-*d*, *Z*-isomer) δ 10.97 (s, 1 H, NH), 7.76 – 7.66 (m, 4 H, Ar), 7.59 – 7.34 (m, 6 H, Ar), 7.12 – 6.68 (m, 4 H, Ar), 3.82 (d, *J* = 14.7 Hz, 2 H, CH_2), 3.70 (s, 3 H, O-Me), 1.58 (s, 9 H, *t*Bu).

^{13}C NMR (76 MHz, DEPT, Chloroform-*d*, *Z*-isomer) δ 159.4 (=C–O), 154.6 (C=O), 144.7 (d, *J* = 9.9 Hz, C=N), 139.6 (d, *J* = 3.1 Hz, C_{Ar}), 132.7 (d, *J* = 2.8 Hz, 2 CH_{Ph}), 131.1 (d, *J* = 9.8 Hz, 4 CH_{Ph}), 130.6 (d, *J* = 101.3 Hz, 2 C–P), 128.9 (CH_{Ar}), 128.9 (d, *J* = 12.2 Hz, 4 CH_{Ph}), 119.0 (CH_{Ar}), 115.3 (CH_{Ar}), 111.7 (CH_{Ar}), 80.9 (*C*Me_3), 55.4 (OMe), 33.8 (d, *J* = 63.5 Hz, CH_2–P), 28.5 (3 Me).

^{31}P NMR (122 MHz, Chloroform-*d*, *Z*-isomer) δ 33.49.

^{1}H NMR (300 MHz, Chloroform-*d*, *E*-isomer, characteristic signals) δ 4.15 (d, *J* = 15.3 Hz, 2 H, CH_2)

^{31}P NMR (121.49 MHz, Chloroform-*d*, *E*-isomer) δ 26.96.

HRMS: *m/z* $[M+H]^+$ calcd. for $[C_{26}H_{30}N_2O_4P]^+$: 465.1938; found: 465.1943.

Tert-butyl 2-(2-(diphenylphosphoryl)-1-(3-nitrophenyl)ethylidene)hydrazinecarboxylate (1fa)

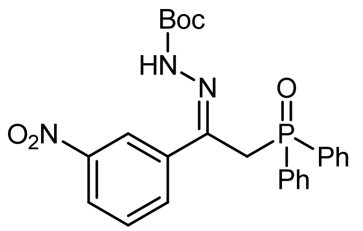

Prepared according to general procedure from 90 mg of bromohydrazone **2f** (0.25 mmol). Yield: 91 mg (63 %). White crystals. Mp 204 – 206 °C (EA).

Mixture of *Z/E* isomers (ratio 20 : 1).

^{1}H NMR (300 MHz, Chloroform-*d*, *Z*-isomer) δ 11.01 (s, 1 H, NH), 8.10 – 7.20 (m, 14 H, Ar), 3.87 (d, *J* = 14.4 Hz, 2 H, CH_2), 1.59 (s, 9 H, *t*-Bu).

^{13}C NMR (76 MHz, DEPT, Chloroform-*d*, *Z*-isomer) δ 154.4 (C=O), 147.9 (C–NO_2), 142.1 (C=N), 139.5 (d, *J* = 2.9 Hz, C_{Ar}), 133.1 (d, *J* = 2.8 Hz, 2 CH_{Ph}), 132.8 (CH_{Ar}), 131.1 (d, *J* = 9.9 Hz, 4 CH_{Ph}), 130.1 (d, *J* = 101.6 Hz, 2 C–P), 129.2 (CH_{Ar}), 129.2 (d, *J* = 12.3 Hz, 4 CH_{Ph}), 123.6 (CH_{Ar}), 120.8 (CH_{Ar}), 81.6 (*C*Me_3), 33.2 (d, *J* = 63.5 Hz, CH_2), 28.4 (3 Me).

^{31}P NMR (122 MHz, Chloroform-*d*, *Z*-isomer) δ 33.15.

^{1}H NMR (300 MHz, Chloroform-*d*, *E*-isomer, characteristic signals) δ 8.86 (br s, 1 H, NH), 4.20 (d, *J* = 15.2 Hz, 2 H, CH_2).

^{31}P NMR (121.49 MHz, Chloroform-*d*, *E*-isomer) δ 26.44.

HRMS: *m/z* $[M+H]^+$ calcd. for $[C_{25}H_{27}N_3O_5P]^+$: 480.1682; found: 480.1670.

FT-IR (thin layer, characteristic bands): ν 3195 (s, br, NH), 2979 (m, CH), 2927 (m, CH), 1730 (s, C=O), 1530 (s, NO_2), 1350 (s, sh,), 1248 (m, sh, P–O), 1152 (s, sh, P–O) cm^{-1}.

Tert-butyl 2-(1-(3,5-bis(trifluoromethyl)phenyl)-2-(diphenylphosphoryl)ethylidene)hydrazinecarboxylate (1ga)

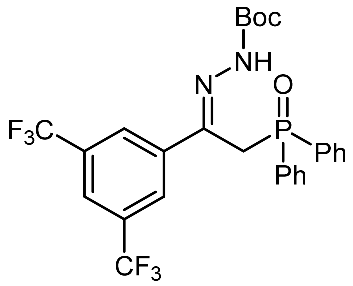

Prepared according to general procedure from 112 mg of bromohydrazone **2g** (0.25 mmol). Yield: 104 mg (73 %). White crystals. Mp 159 –162 °C (EA).

Single *Z* isomer.

^{1}H NMR (300 MHz, Chloroform-*d*) δ 11.04 (s, 1 H, NH), 7.77 – 7.39 (m, 13 H, Ar), 3.85 (d, *J* = 14.3 Hz, 2 H, CH_2), 1.59 (s, 9 H, *t*-Bu).

^{13}C NMR (76 MHz, DEPT, Chloroform-d) δ 154.3 (C=O), 141.8 (d, *J* = 8.3 Hz, C=N), 140.0 (d, *J* = 3.0 Hz, C_{Ar}), 133.2 (d, *J* = 2.9 Hz, 2 CH_{Ph}), 131.4 (q, *J* = 33.4 Hz, 2 *C*-CF_3), 131.0 (d, *J* = 9.9 Hz, 4 CH_{Ph}), 130.0 (d, *J* = 101.7 Hz, 2 C–P), 129.1 (d, *J* = 12.2 Hz, 4 CH_{Ph}), 126.4 (m, 2 CH_{Ar}), 123.0 (q, *J* = 272.9 Hz, 2 CF_3), 122.2 (m, CH_{Ar}), 81.6 (*C*Me_3), 33.4 (d, *J* = 63.2 Hz, CH_2), 28.4 (3 Me).

^{19}F NMR (282 MHz, Chloroform-*d*) δ -62.85.

^{31}P NMR (122 MHz, Chloroform-*d*) δ 33.10.

HRMS: *m/z* $[M+H]^+$ calcd. for $[C_{27}H_{26}F_6N_2O_3P]^+$: 571.1579; found: 571.1575.

Tert-butyl 2-(2-(diphenylphosphoryl)-1-(thiophen-2-yl)ethylidene)hydrazinecarboxylate (1ha)

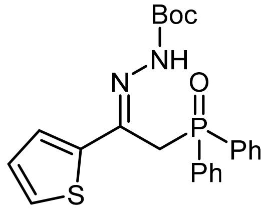

Prepared according to general procedure from 78 mg of bromohydrazone **2h** (0.25 mmol). Yield: 42 mg (38 %). White crystals. Mp 185 – 187 °C (EA).

Mixture of *Z/E* isomers (ratio 25 : 1).

^{1}H NMR (300 MHz, Chloroform-*d*, *Z*-isomer) δ 10.84 (s, 1H, NH), 7.83 – 7.69 (m, 4H, Ph), 7.58 – 7.38 (m, 6H, Ph), 7.14 (dd, *J* = 4.7, 1.5 Hz, 1H, thienyl), 6.77 – 6.65 (m, 2H, thienyl), 3.80 (d, *J* = 14.1 Hz, 2H, CH_2), 1.57 (s, 9H, *t*-Bu).

^{13}C NMR (76 MHz, Chloroform-*d,* DEPT, *Z*-isomer) δ 154.3 (C=O), 143.3 (d, *J* = 3.5 Hz, C_{Ar}), 140.6 (d, *J* = 10.0 Hz, C=N), 132.9 (d, *J* = 2.9 Hz, 2 CH_{Ph}), 131.1 (d, *J* = 9.8 Hz, 4 CH_{Ph}), 130.3 (d, *J* = 101.8 Hz, 2 C_{Ph}), 128.9 (d, *J* = 12.3 Hz, 4 CH_{Ph}), 127.9 (CH_{Ar}), 126.7 (CH_{Ar}), 125.7 (CH_{Ar}), 81.0 (*C*Me_3), 33.9 (d, *J* = 63.9 Hz, CH_2), 28.5 (3 Me).

^{31}P NMR (122 MHz, Chloroform-*d*, *Z*-isomer) δ 33.26.

^{1}H NMR (300 MHz, Chloroform-d, *E*-isomer, characteristic signals) δ 8.89 (br s, 1 H, NH), 4.07 (d, J = 15.3 Hz, 2 H, CH_2).

^{31}P NMR (122 MHz, Chloroform-*d*, *Z*-isomer) δ 26.84.

HRMS: *m/z* $[M+H]^+$ calcd. for $[C_{23}H_{26}N_2O_3PS]^+$: 441.1396; found: 441.1409.

Tert-butyl 2-(2-(diphenylphosphoryl)-3,4-dihydronaphthalen-1(2 H)-ylidene)hydrazinecarboxylate (1ia)

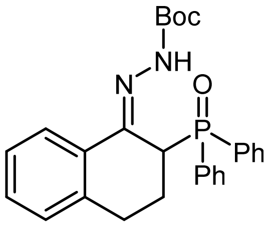

Prepared according to general procedure from 74 mg of chlorohydrazone **2i** (0.25 mmol). Yield: 81 mg (70 %). White crystals. Mp 186 – 187 °C (EA).

Single *Z* isomer.

^{1}H NMR (300 MHz, Chloroform-*d*) δ 11.05 (s, 1H, NH), 7.85 – 7.74 (m, 2H), 7.66 – 7.39 (m, 6H), 7.32 – 7.21 (m, 2H), 7.18 – 7.03 (m, 3H), 6.90 (d, *J* = 7.5 Hz, 1H) (2 Ph and Ar), 4.10 (ddd, *J* = 21.1, 8.7, 7.3 Hz, 1H, CH-P), 2.70 – 2.41 (m, 2H, CH_2), 2.15 – 1.80 (m, 2H, CH_2), 1.55 (s, 9H, *t*-Bu).

^{13}C NMR (76 MHz, Chloroform-d) δ 154.5 (C=O), 145.3 (d, *J* = 6.1 Hz, C=N), 138.0 (d, *J* = 1.6 Hz, C_{Ar}), 134.5 (d, *J* = 2.0 Hz, C_{Ar}), 132.7 (d, *J* = 2.8 Hz, CH_{Ph}), 132.6 (d, *J* = 2.8 Hz, CH_{Ph}), 131.9 (d, *J* = 9.5 Hz, 2 CH_{Ph}), 131.8 (d, *J* = 9.5 Hz, 2 CH_{Ph}), 130.2 (d, *J* = 97.4 Hz, C–P), 129.0 (CH_{Ar}), 128.9 (d, *J* = 11.9 Hz, 2 CH_{Ph}), 128.3 (d, *J* = 99.6 Hz, C–P), 128.50 (d, *J* = 11.7 Hz, 2 CH_{Ph}), 129.0, 126.9 and 126.7 (3 CH_{Ar}), 80.6 (*C*Me_3), 41.1 (d, *J* = 63.2 Hz, CH–P), 28.4 (3 Me), 27.6 (d, *J* = 8.9 Hz, CH_2), 25.5 (CH_2).

^{31}P NMR (122 MHz, Chloroform-*d*) δ 38.76.

HRMS: *m/z* $[M+H]^+$ calcd. for $[C_{27}H_{30}N_2O_3P]^+$: 461.1989; found: 461.1975.

Tert-butyl 2-(3-(diphenylphosphoryl)-1-ethoxy-1-oxopropan-2-ylidene)hydrazinecarboxylate (1ja)

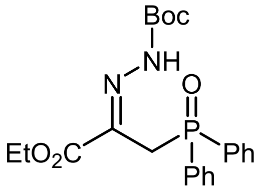

Prepared according to general procedure from 77 mg of bromohydrazone **2j** (0.25 mmol). Yield: 62 mg (58 %). White crystals. Mp 158 – 161 °C (EA).

Single *Z* isomer.

^{1}H NMR (300 MHz, Chloroform-*d*) δ 11.40 (s, 1 H, NH), 7.82 – 7.72 (m, 4 H, Ph), 7.59 – 7.42 (m, 6 H, Ph), 4.11 (q, *J* = 7.1 Hz, 2 H, O-CH_2), 3.82 (d, *J* = 14.7 Hz, 2 H, CH_2-P), 1.54 (s, 9 H, *t*-Bu), 1.16 (t, *J* = 7.1 Hz, 3H, CH_3).

^{13}C NMR (76 MHz, DEPT, Chloroform-*d*) δ 164.5 (d, *J* = 4.0 Hz, C=O), 152.9 (N–C=O), 134.9 (d, *J* = 8.8 Hz, C=N), 132.7 (d, *J* = 2.9 Hz, 2 CH_{Ph}), 130.9 (d, *J* = 10.0 Hz, 4 CH_{Ph}), 130.4 (d, *J* = 102.0 Hz, 2 C–P), 128.9 (d, *J* = 12.3 Hz, 4 CH_{Ph}), 82.1 (*C*Me_3), 62.2 (CH_2), 31.2 (d, *J* = 65.4 Hz, CH_2–P), 28.2 (3 Me), 14.0 (Me).

^{31}P NMR (122 MHz, Chloroform-*d*) δ 34.17.

HRMS: *m/z* [M+H]$^+$ calcd. for [$C_{22}H_{28}N_2O_5P^+$]$^+$: 431.1737; found: 431.1730.

FT-IR (thin layer, characteristic bands): ν 3160 (s, br, NH), 2981 (m, sh, CH), 1747 (s, C=O), 1710 (s, C=O), 1591 (w), 1535 (m, sh), 1243 (s, sh), 1153 (s, sh, P-O), 751 (s, sh).

Tert-butyl 2-(1-(diphenylphosphoryl)-3,3-dimethylbutan-2-ylidene)hydrazinecarboxylate (1ka)

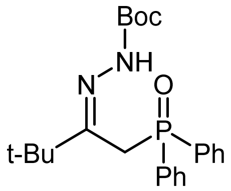

Prepared according to general procedure from 62 mg of chlorohydrazone **2k** (0.25 mmol). Yield: 71 mg (69 %). White crystals. Mp 192 – 194 °C (EA).

Single *Z* isomer.

^{1}H NMR (300 MHz, Chloroform-*d*) δ 11.14 (s, 1 H, NH), 7.72 (ddd, *J* = 11.8, 8.2, 1.5 Hz, 4 H, Ph), 7.62 – 7.40 (m, 6 H, Ph), 3.36 (d, *J* = 15.4 Hz, 2 H, CH_2), 1.53 (s, 9 H, *t*-Bu), 0.79 (s, 9 H, *t*-Bu).

^{13}C NMR (76 MHz, DEPT, Chloroform-*d*) δ 154.8 (C=O), 151.6 (d, *J* = 9.5 Hz, C=N), 132.8 (d, *J* = 2.9 Hz, 2 CH_{Ph}), 131.3 (d, *J* = 9.6 Hz, 4 CH_{Ph}), 130.9 (d, *J* = 101.3 Hz, 2 C–P), 128.9 (d, *J* = 12.1 Hz, 4 CH_{Ph}), 80.3 (*C*Me_3), 39.0 (d, *J* = 2.8 Hz, *C*Me_3), 31.6 (d, *J* = 64.4 Hz, CH_2P), 28.5 (3 Me), 28.0 (3 Me).

^{31}P NMR (122 MHz, Chloroform-*d*) δ 32.76.

HRMS: *m/z* [M+H]$^+$ calcd. for [$C_{23}H_{32}N_2O_3P$]$^+$: 415.2137; found: 415.2145.

Benzyl 2-(2-(diphenylphosphoryl)-1-phenylethylidene)hydrazinecarboxylate (1la)

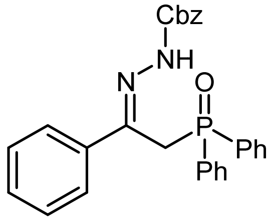

Prepared according to general procedure from 87 mg of bromohydrazone **2l** (0.25 mmol). Yield: 84 mg (72 %). White crystals. Mp 170 – 173 °C (EA).

Mixture of *Z/E* isomers (ratio 50 : 1).

^{1}H NMR (300 MHz, Chloroform-*d*, *Z*-isomer) δ 11.35 (s, 1 H, NH), 7.69 (ddd, *J* = 12.1, 8.3, 1.4 Hz, 4 H, Ph), 7.55 – 7.07 (m, 16 H, Ph), 5.32 (s, 2 H, CH_2-Ph), 3.84 (d, *J* = 14.8 Hz, 2 H, CH_2P).

^{13}C NMR (76 MHz, DEPT, Chloroform-*d*, *Z*-isomer) δ 155.5 (C=O), 146.4 (d, *J* = 9.5 Hz, C=N), 137.8 (d, *J* = 2.9 Hz, C_{Ph}), 136.4 (C_{Ph}), 132.7 (d, *J* = 2.9 Hz, 2 CH_{Ph}), 131.0 (d, *J* = 9.9 Hz, 4 CH_{Ph}),

130.2 (d, *J* = 101.6 Hz, 2 C–P), 128.8 (d, *J* = 12.2 Hz, 4 CH_{Ph}), 129.1, 128.5, 128.4, 128.1 and 126.6 (10 CH_{Ph}), 67.2 (CH_2–Ph), 33.7 (d, *J* = 62.9 Hz, CH_2–P).

^{31}P NMR (122 MHz, Chloroform-*d*, *Z*-isomer) δ 33.82.

^{1}H NMR (300 MHz, Chloroform-d, *E*-isomer, characteristic signals) δ 8.41 (br s, 1 H, NH), 4.15 (d, J = 15.4 Hz, 2 H, CH_2–P).

^{31}P NMR (122 MHz, Chloroform-*d*, *Z*-isomer) δ 28.32.

HRMS: *m/z* $[M+H]^+$ calcd. for $[C_{28}H_{26}N_2O_3P]^+$: 469.1665; found: 469.1676.

***Tert*-butyl 2-(2-(bis(4-methoxyphenyl)phosphoryl)-1-phenylethylidene)hydrazinecarboxylate (1ab)**

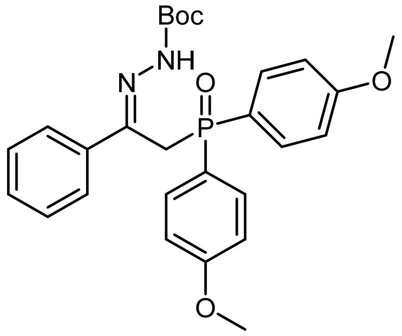

Prepared according to general procedure from 78 mg of bromohydrazone **2a** (0.25 mmol). Yield: 94 mg (76 %). White crystals. Mp 178 – 180 °C (EA).

Single *Z* isomer.

^{1}H NMR (300 MHz, Chloroform-*d*) δ 11.00 (s, 1 H, NH), 7.56 (dd, *J* = 11.5, 8.5 Hz, 4 H, Ar), 7.30 (d, *J* = 7.9 Hz, 2 H, Ar), 7.20 – 7.03 (m, 3H, Ar), 6.85 (dd, *J* = 8.5, 1.9 Hz, 4 H, Ar), 3.75 (s, 6 H, 2 OMe), 3.73 (d, *J* = 15.1 Hz, 2 H, CH_2), 1.54 (s, 9 H, *t*-Bu).

^{13}C NMR (76 MHz, Chloroform-*d*) δ 162.9 (d, *J* = 2.9 Hz, 2 =C–O), 154.6 (C=O), 145.3 (d, *J* = 9.6 Hz, C=N), 138.0 (d, *J* = 2.9 Hz, C_{Ph}), 132.9 (d, *J* = 11.2 Hz, 4 CH_{Ar}), 128.7 (CH_{Ph}), 127.9 (2 CH_{Ph}), 126.5 (2 CH_{Ph}), 121.9 (d, *J* = 108.1 Hz, 2 C–P), 114.3 (d, *J* = 13.1 Hz, 4 CH_{Ar}), 80.6 (*C*Me_3), 55.4 (2 OMe), 33.9 (d, *J* = 64.3 Hz, CH_2P), 28.3 (3 Me).

^{31}P NMR (122 MHz, Chloroform-*d*) δ 33.34.

HRMS: *m/z* $[M+H]^+$ calcd. for $[C_{27}H_{32}N_2O_5P]^+$: 495.2043; found: 495.2035.

FT-IR (thin layer): ν 3173 (s, br), 2978 (w, sh), 1731 (m), 1597 (s), 1570 (w), 1504 (m), 1367 (w), 1296 (m, sh), 1257 (s), 1168 (s, sh), 1122 (s), 1065 (w), 1027 (w), 886 (w), 830 (w), 806 (m), 755 (s), 694 (w), 667 (w), 610 (m, sh).

***Tert*-butyl 2-(2-(di(thiophen-2-yl)phosphoryl)-1-phenylethylidene)hydrazinecarboxylate (1ac)**

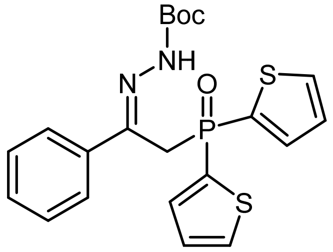

Prepared according to general procedure from 78 mg of bromohydrazone **2a** (0.25 mmol). Yield: 83 mg (74 %). White crystals. Mp 177 – 180 °C (EA).

Single *Z* isomer.

^{1}H NMR (300 MHz, Chloroform-*d*) δ 10.64 (s, 1 H, NH), 7.71 (td, *J* = 4.7, 1.1 Hz, 2 H, Ar), 7.62 (ddd, *J* = 7.5, 3.6, 1.1 Hz, 2 H, Ar), 7.47 – 7.36 (m, 2 H, Ar), 7.25 – 7.07 (m, 5H, Ar), 3.85 (d, *J* = 15.7 Hz, 2 H, CH_2), 1.56 (s, 9 H, *t*-Bu).

^{13}C NMR (76 MHz, DEPT, Chloroform-*d*) δ 154.4 (C=O), 144.0 (d, *J* = 10.3 Hz, C=N), 137.6 (d, *J* = 3.2 Hz, C_{Ph}), 136.7 (d, *J* = 10.4 Hz, 2 CH_{Ar}), 134.6 (d, *J* = 5.3 Hz, 2 CH_{Ar}), 131.2 (d, *J* = 117.4 Hz, 2 C–P), 129.0 (CH_{Ph}), 128.7 (d, *J* = 14.6 Hz, 2 CH_{Ar}), 128.0 (2 CH_{Ph}), 126.4 (2 CH_{Ph}), 80.9 (CMe_3), 36.5 (d, *J* = 73.1 Hz, CH_2), 28.4 (3 Me).

^{31}P NMR (122 MHz, Chloroform-*d*) δ 20.51.

HRMS: *m/z* $[M+H]^+$ calcd. for $[C_{21}H_{24}N_2O_3PS_2]^+$: 447.0960; found: 447.0950.

Tert-butyl 2-(2-(dibenzylphosphoryl)-1-phenylethylidene)hydrazinecarboxylate (1ad)

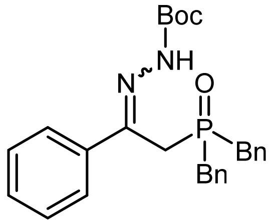

Prepared according to general procedure from 78 mg of bromohydrazone **2a** (0.25 mmol). Yield: 52 mg (45 %). White foam.

Mixture of *Z/E* isomers (ratio 2 : 1).

^{1}H NMR (300 MHz, Chloroform-*d*, *Z*-isomer) δ 10.43 (s, 1 H, NH), 7.67 – 7.56 (m, 4 H), 7.48 – 7.17 (m, 11 H), 3.21 (d, *J* = 13.7 Hz, 2 H, CH_2–P), 3.09 (m, 4 H, 2 CH_2Ph), 1.55 (s, 9 H, *t*-Bu).

^{13}C NMR (76 MHz, DEPT, Chloroform-*d*, *Z*-isomer) δ 154.4 (C=O), 144.3 (C=N), 137.6 (d, *J* = 2.9 Hz, C_{Ph}) , 130.5 (d, *J* = 7.5 Hz, 2 C_{Bn}), 129.9 (d, *J* = 5.4 Hz, 4 CH_{Bn}), 129.6 (CH_{Ph}), 129.1 (d, *J* = 2.5 Hz, 4 CH_{Bn}), 128.6 (2 CH_{Ph}), 127.5 (d, *J* = 3.0 Hz, 2 CH_{Bn}), 126.5 (2 CH_{Ph}), 80.8 (CMe_3), 36.2 (d, *J* = 60.9 Hz, 2 CH_2Ph), 29.9 (d, *J* = 57.8 Hz, CH_2–P), 28.4 (3 Me).

^{31}P NMR (122 MHz, Chloroform-*d*, *Z*-isomer) δ 45.90.

^{1}H NMR (300 MHz, Chloroform-*d*, *E*-isomer) δ 7.82 (br, 1 H, NH), 7.67 – 7.56, 7.49 – 7.14 and 7.07 – 7.01 (3 m, 15 H), 3.61 – 3.32 (m, 4 H, 2 CH_2Ph), 2.81 (d, *J* = 14.3 Hz, 2 H, CH_2–P), 1.55 (s, 9 H, *t*-Bu).

^{13}C NMR (76 MHz, DEPT, Chloroform-*d*, *E*-isomer, characteristic signals) δ 154.4 (C=O), 132.2 (d, *J* = 7.8 Hz, 2 C_{Bn}), 130.0 (d, *J* = 5.2 Hz, CH_{Bn}), 129.8 (CH_{Ph}), 128.8 (d, *J* = 2.2 Hz, 2 CH_{Bn}), 127.0 (d, *J* = 2.4 Hz, 2 CH_{Bn}), 126.6 (2 CH_{Ph}), 36.4 (d, *J* = 61.0 Hz, 2 CH_2Ph), 28.4 (3 Me).

^{31}P NMR (122 MHz, Chloroform-*d*, *E*-isomer) δ 44.90.

HRMS: *m/z* $[M+H]^+$ calcd. for $[C_{27}H_{32}N_2O_3P]^+$: 463.2145; found: 463.2142.

(2-Hydrazono-2-phenylethyl)diphenylphosphine oxide 4aa[9]

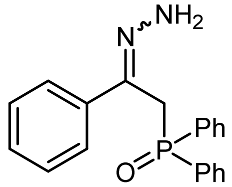

To a solution of Boc-hydrazone **1aa** (0.25 mmol, 109 mg) in DCM (0.5 ml) was added TFA (2.5 mmol, 191 µl). The mixture was stirred at rt for 30 min, then diluted with DCM (10 ml) and subjected to a standard aqueous work-up with DCM/saturated water solution of $NaHCO_3$ (10 mL). The organic extract was concentrated in vacuum to give 75 mg (90 %) of unprotected hydrazone **4aa** as a yellowish foam.

Mixture of *Z/E* isomers (ratio 2 : 1).

^{1}H NMR (300 MHz, Chloroform-d, *Z*-isomer) δ 7.78 – 7.63 and 7.53 – 7.05 (2 m, 15 H, 3 Ph), 6.72 (s, 2 H, NH_2), 3.89 (d, J = 14.5 Hz, 2 H).

^{13}C NMR (76 MHz, DEPT, Chloroform-d, *Z*-isomer) δ 142.7 (d, *J* = 10.1 Hz, C=N), 138.9 (d, *J* = 2.7 Hz, C_{Ph}), 132.3 (d, *J* = 2.8 Hz, 2 $CH_{Ph\text{-}P}$), 131.4 (d, *J* = 99.8 Hz, 2 C–P), 131.0 (d, *J* = 9.7 Hz, 4 $CH_{Ph\text{-}P}$), 128.7 (d, *J* = 12.0 Hz, 4 $CH_{Ph\text{-}P}$), 128.3 (CH_{Ph}), 128.0 (2 CH_{Ph}), 125.7 (2 CH_{Ph}), 31.9 (d, *J* = 65.5 Hz, CH_2).

^{31}P NMR (122 MHz, Chloroform-d, *Z*-isomer) δ 32.55.

^{1}H NMR (300 MHz, Chloroform-d, *E*-isomer) δ 7.90 (d, *J* = 8.2 Hz, 2 H, Ph), 7.78 – 7.63 and 7.53 – 7.05 (2 m, 13 H, Ph), 6.72 (s, 2 H, NH_2), 4.01 (d, *J* = 15.8 Hz, 2 H).

^{13}C NMR (76 MHz, DEPT, Chloroform-d, *E*-isomer) δ 142.7 (d, *J* = 10.1 Hz, C=N), 136.9 (C_{Ph}), 132.7 (d, *J* = 101.0 Hz, 2 C–P), 131.8 (d, *J* = 2.7 Hz, 2 $CH_{Ph\text{-}P}$), 131.1 (d, *J* = 10.5 Hz, 4 $CH_{Ph\text{-}P}$), 130.5 (CH_{Ph}), 128.3 (d, *J* = 12.2 Hz, 4 $CH_{Ph\text{-}P}$), 128.0 (2 CH_{Ph}), 127.9 (2 CH_{Ph}), 33.7 (d, *J* = 61.4 Hz, CH_2).

^{31}P NMR (122 MHz, Chloroform-d, *E*-isomer) δ 27.37.

HRMS: m/z $[M+H]^+$ calcd. for $[C_{20}H_{20}N_2OP]^+$: 335.1308; found: 335.1318.

[9] Bestmann, H. J.; Klein, O. *Tetrahedron Lett.* **1966**, *49*, 6181.

((*Z*)-2-(((*E*)-2-hydroxybenzylidene)hydrazono)-2-phenylethyl)diphenylphosphine oxide 5aa

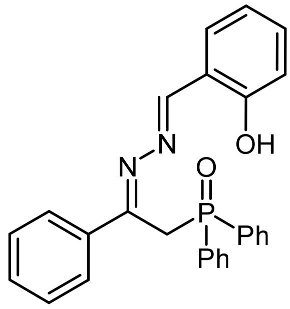

To a stirred solution of hydrazone **4aa** (0.5 mmol, 167 mg, *Z*/*E* = 2 : 1) in EtOH (3 ml) was added salicylaldehyde (0.6 mmol, 63 µl) and acetic acid (0.5 mmol, 29 µl). The mixture refluxed for 2 hours and the resulting solution was concentrated in a vacuum. The residue was dissolved in ethyl acetate (50 ml) and washed with saturated aqueous solution of $NaHCO_3$ (50 ml). After standard aqueous work-up, the combined organic phase was dried with Na_2SO_4 and concentrated in a vacuum. The residue was subjected to a column chromatography on silica gel to give two fractions: the first one contained 110 mg (50%) of product **5aa** and the second one contained 51 mg (31%) of unreacted (*E*)-**4aa**.

M.p. = 197–199 °C ($CHCl_3$). R_f = 0.65 (EtOAc)

Single isomer.

^{1}H NMR (300 MHz, Chloroform-d, HMBC, NOESY) δ 11.26 (s, 1 H, OH), 8.12 (s, 1 H, HC=N), 8.09 (m, 2 H, 2 CH_{Ph}), 7.80 – 7.69 (m, 4 H, 4 $CH_{Ph\text{-}P}$), 7.53 – 7.26 (m, 10 H, 6 $CH_{Ph\text{-}P}$, 3 CH_{Ph} and CH_{Ar}), 7.12 (d, *J* = 7.7 Hz, 1 H, CH_{Ar}), 7.03 (d, *J* = 8.3 Hz, 1 H, CH_{Ar}), 6.93 (dd, *J* = 7.7, 7.5 Hz, 1 H, CH_{Ar}), 4.21 (d, *J* = 15.3 Hz, 2 H).

^{13}C NMR (76 MHz, Chloroform-d, HMBC) δ 162.9 (d, *J* = 1.9 Hz, HC=N), 160.4 (d, *J* = 8.6 Hz, C=N), 159.4 (=C–OH), 136.9 (C_{Ph}), 133.0 (CH_{Ar}), 132.4 (CH_{Ar}), 132.07 (d, *J* = 2.9 Hz, 2 $CH_{Ph\text{-}P}$), 131.8 (d, *J* = 100 Hz, 2 C–P), 131.2 (d, *J* = 9.6 Hz, 4 $CH_{Ph\text{-}P}$), 130.9 (CH_{Ph}), 128.54 (d, *J* = 12.1 Hz, 4 $CH_{Ph\text{-}P}$), 128.5 (2 CH_{Ph}), 128.3 (2 CH_{Ph}), 119.5 (CH_{Ar}), 118.1 (C_{Ar}), 116.9 (CH_{Ar}), 34.21 (d, *J* = 60.5 Hz, CH_2).

^{31}P NMR (122 MHz, Chloroform-d) δ 26.90.

Characteristic 2D NOESY correlations: OH/CH_2P.

HRMS: m/z $[M+H]^+$ calcd. for $[C_{27}H_{24}N_2O_2P]^+$: 439.1570; found: 439.1567.

Tert-butyl 2-(2-(diphenylphosphoryl)-1-phenylethyl)hydrazinecarboxylate (6aa)

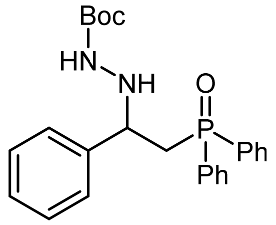

In the reaction vial, equipped with a magnetic stirring bar, were placed a solution of hydrazone **1aa** (0.25 mmol, 109 mg) in MeOH (1 ml) and 10%-Pd/C (54 mg). The vial was placed in steel autoclave, which was then flushed and filled with H_2 to a pressure of 40 bar. After stirring the mixture for 3 h at 60 °C, the autoclave was cooled to rt and slowly depressurized. The solution was centrifuged and concentrated in vacuum to give 91 mg (83 %) of hydrazide **6aa** as a white foam.

^{1}H NMR (300 MHz, 333 K, DMSO-d_6) δ 7.89 – 7.67, 7.61 – 7.36 and 7.32 – 7.08 (3 m, 4 H, 6 H and 5 H, 3 Ph), 4.36 (ddd, J = 6.7, 6.7, 6.6 Hz, 1 H, CH-N), 3.24 (br s, 1 H, NH), 2.89 – 2.69 (m, 2 H, CH_2), 1.31 (s, 9 H, 3 Me).

^{13}C NMR (76 MHz,333 K, DMSO-d_6) δ 155.8 (d, J = 3.2 Hz, C=O), 141.4 (d, J = 8.3 Hz, $C_{Ph\text{-}C}$), 134.3 (d, J = 98.0 Hz, $C_{Ph\text{-}P}$), 132.8 (d, J = 97.4 Hz, $C_{Ph\text{-}P}$), 131.3 (d, J = 2.6 Hz, $CH_{Ph\text{-}P}$), 131.0 (d, J = 2.7 Hz, $CH_{Ph\text{-}P}$), 130.4 (d, J = 9.3 Hz, 2 $CH_{Ph\text{-}P}$), 130.0 (d, J = 9.3 Hz, 2 $CH_{Ph\text{-}P}$), 128.3 (d, J = 11.5 Hz, 2 $CH_{Ph\text{-}P}$), 128.1 (d, J = 11.6 Hz, 2 $CH_{Ph\text{-}P}$), 127.7 (2 $CH_{Ph\text{-}C}$), 127.5 (2 $CH_{Ph\text{-}C}$), 126.9 ($CH_{Ph\text{-}C}$), 78.2 (CMe$_3$), 58.5 (CH), 35.2 (d, J = 69.3 Hz, CH_2-P), 27.9 (3 Me).

^{31}P NMR (122 MHz, 333 K, DMSO-d_6) δ 28.75.

HRMS: m/z $[M+H]^+$ calcd. for $[C_{25}H_{30}N_2O_3P]^+$: 437.1989; found: 437.1982.

DFT calculations

DFT calculations were performed with ORCA v.5.0.3[10] quantum chemistry package. wB97M-D4DFT functional with def2-TZVP basis set was used for geometry optimization and calculations of thermodynamics. Calculations were performed in CH_2Cl_2 (CPCM). Cartesian coordinates are given in angstroms; absolute energies for all substances are given in hartrees. Analysis of vibrational frequencies was performed for all optimized structures. All structures were characterized by only real vibrational frequencies.

(*E*)-**1aa**

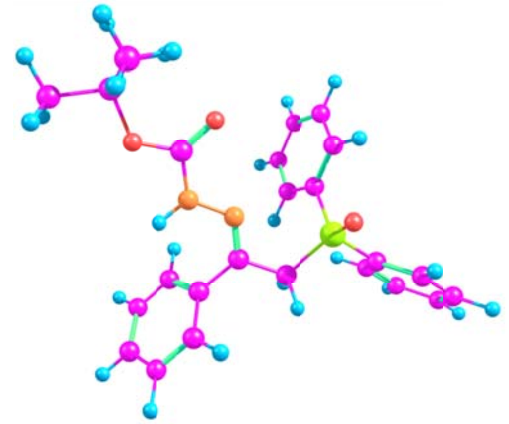

6	0.663540000	0.423480000	1.573194000
6	0.271568000	0.058443000	0.182628000
6	-0.800481000	-0.982847000	0.028076000
7	0.759996000	0.573641000	-0.872433000
7	1.739549000	1.506797000	-0.749472000
15	-1.266966000	-1.397672000	-1.676253000
8	-0.210056000	-2.095855000	-2.461230000
6	0.381541000	1.695132000	2.066129000
6	0.729714000	2.030848000	3.366381000
6	1.364369000	1.100841000	4.178494000
6	1.646238000	-0.168209000	3.690270000
6	1.292106000	-0.508873000	2.393277000
6	2.242028000	2.101072000	-1.877302000
8	3.221174000	2.933822000	-1.507681000
8	1.847065000	1.898893000	-3.002489000
6	3.955109000	3.733864000	-2.499798000
6	4.938905000	4.509105000	-1.638257000
6	4.696169000	2.819876000	-3.463673000
6	3.005741000	4.685728000	-3.211437000
6	-1.867861000	0.117465000	-2.450211000
6	-1.748037000	0.227610000	-3.831833000
6	-2.198035000	1.367514000	-4.482324000
6	-2.768847000	2.401914000	-3.754423000
6	-2.889053000	2.296760000	-2.374536000

[10] Neese, F. Software update: The ORCA program system—Version 5.0. *WIREs Comput Mol Sci*. **2022**, e1606.

6	-2.439349000	1.158728000	-1.723367000
6	-2.724238000	-2.448655000	-1.450664000
6	-2.648937000	-3.773973000	-1.865839000
6	-3.739671000	-4.618426000	-1.705346000
6	-4.907623000	-4.140495000	-1.129567000
6	-4.988312000	-2.816267000	-0.714518000
6	-3.901166000	-1.972596000	-0.875616000
1	-0.479522000	-1.925106000	0.481000000
1	-1.692417000	-0.679948000	0.581286000
1	2.140515000	1.735849000	0.148955000
1	-0.113640000	2.417772000	1.430477000
1	0.503753000	3.018316000	3.745012000
1	1.637453000	1.363898000	5.191336000
1	2.140589000	-0.894755000	4.320545000
1	1.507886000	-1.499017000	2.013564000
1	5.594576000	3.826460000	-1.099178000
1	5.548764000	5.150912000	-2.272019000
1	4.408294000	5.131480000	-0.918732000
1	5.353943000	3.426263000	-4.085660000
1	4.009829000	2.277592000	-4.106778000
1	5.308658000	2.107848000	-2.910906000
1	2.445435000	5.271693000	-2.483056000
1	3.591122000	5.370901000	-3.823871000
1	2.310752000	4.151750000	-3.852162000
1	-1.288888000	-0.573930000	-4.394167000
1	-2.097418000	1.449553000	-5.555930000
1	-3.116072000	3.292588000	-4.260506000
1	-3.327184000	3.104503000	-1.804274000
1	-2.525805000	1.097649000	-0.646845000
1	-1.735494000	-4.136571000	-2.316376000
1	-3.676396000	-5.647744000	-2.031215000
1	-5.757816000	-4.797581000	-1.005093000
1	-5.899520000	-2.441729000	-0.268453000
1	-3.976678000	-0.941211000	-0.556432000

Electronic energy	-1646.46739056
Zero point energy	0.47292736
Total thermal energy	-1645.96445739
Total enthalpy =	-1645.96351318
Final entropy term	0.08818340
Final Gibbs free energy	-1646.05169657
Number of imaginary vibrational frequencies = 0	

(*Z*)-**1aa**

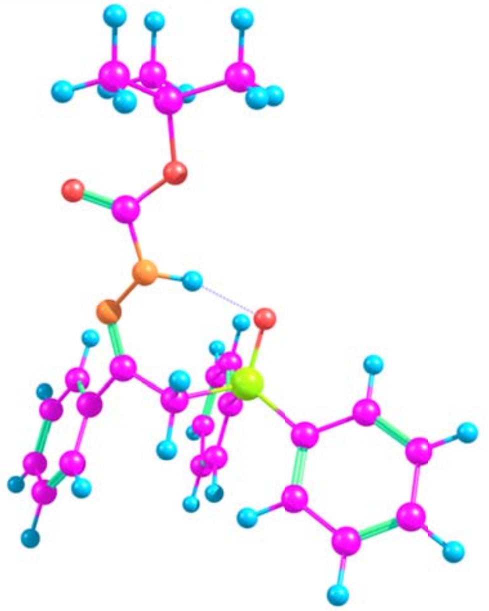

6	0.993160000	0.011083000	-2.081816000
6	1.182099000	0.339675000	-0.645487000
6	0.141743000	1.184928000	0.043083000
7	2.212076000	-0.151965000	-0.073672000
7	2.333745000	0.049402000	1.278969000
15	-1.036969000	0.148037000	0.956091000
8	-0.382348000	-0.508625000	2.130435000
6	1.297131000	-1.269617000	-2.537637000
6	1.107536000	-1.604944000	-3.868821000
6	0.614530000	-0.662788000	-4.761742000
6	0.313928000	0.616785000	-4.315521000
6	0.495200000	0.949999000	-2.981883000
6	3.502470000	-0.360811000	1.874266000
8	3.313763000	-0.372541000	3.198593000
8	4.525194000	-0.629111000	1.286713000
6	4.414627000	-0.673897000	4.124169000
6	4.896657000	-2.101557000	3.915964000
6	5.529721000	0.348350000	3.963957000
6	3.752925000	-0.529323000	5.485330000
6	-1.630796000	-1.053191000	-0.247295000
6	-1.325249000	-2.393394000	-0.037307000
6	-1.691568000	-3.344464000	-0.979770000
6	-2.357071000	-2.956282000	-2.132843000
6	-2.663252000	-1.617191000	-2.345871000
6	-2.302327000	-0.667193000	-1.405726000
6	-2.408315000	1.212355000	1.435723000
6	-2.908543000	2.230251000	0.625739000
6	-3.980086000	2.997064000	1.056760000
6	-4.556344000	2.753467000	2.296337000
6	-4.059392000	1.743526000	3.108379000

6	-2.987313000	0.974936000	2.680708000
1	0.595598000	1.858653000	0.772773000
1	-0.399739000	1.781250000	-0.685289000
1	1.489600000	0.005519000	1.847455000
1	1.663631000	-2.004372000	-1.835361000
1	1.335906000	-2.606431000	-4.207535000
1	0.461789000	-0.925477000	-5.799764000
1	-0.064469000	1.358095000	-5.006173000
1	0.259331000	1.952700000	-2.652456000
1	5.604220000	-2.352190000	4.705854000
1	4.056825000	-2.793478000	3.976359000
1	5.389061000	-2.219970000	2.955612000
1	6.253841000	0.205573000	4.765556000
1	5.127261000	1.358174000	4.042818000
1	6.038533000	0.239155000	3.011035000
1	2.922407000	-1.227583000	5.581390000
1	4.480188000	-0.741769000	6.267365000
1	3.379046000	0.484668000	5.621947000
1	-0.796464000	-2.684575000	0.859340000
1	-1.452373000	-4.385959000	-0.814462000
1	-2.637416000	-3.696574000	-2.869913000
1	-3.179185000	-1.314238000	-3.246457000
1	-2.536780000	0.373252000	-1.586134000
1	-2.469096000	2.439677000	-0.339432000
1	-4.361803000	3.787785000	0.425803000
1	-5.390604000	3.354994000	2.630924000
1	-4.503418000	1.556131000	4.076328000
1	-2.590024000	0.193427000	3.313389000

Electronic energy	-1646.47576784
Zero point energy	0.47412976
Total thermal energy	-1645.97225680
Total enthalpy =	-1645.97131259
Final entropy term	0.08596267
Final Gibbs free energy	-1646.05727526
Number of imaginary vibrational frequencies = 0	

Detection of AZA intermediate

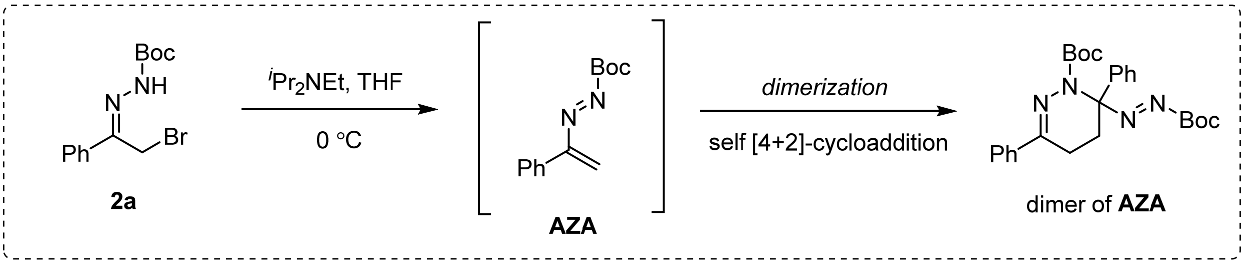

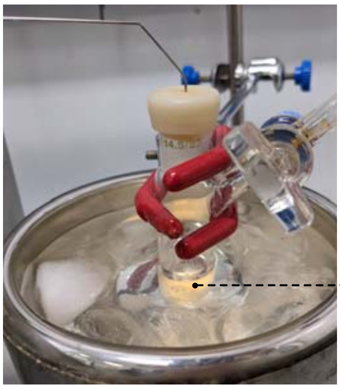

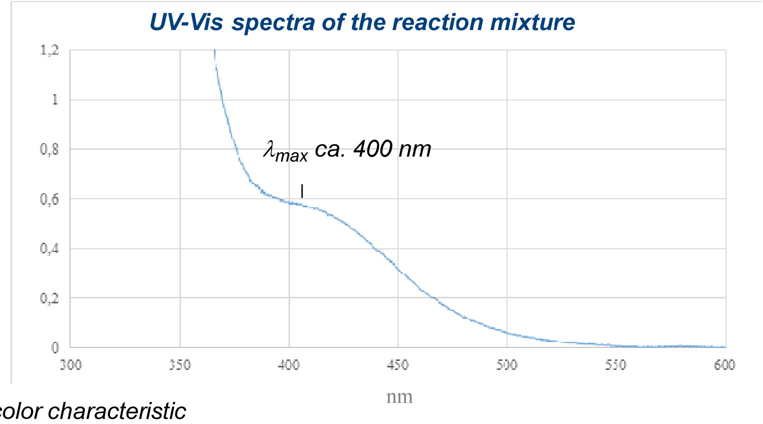

yellow color characteristic for generation of ***AZA***

^{1}H NMR spectra of the reaction mixture

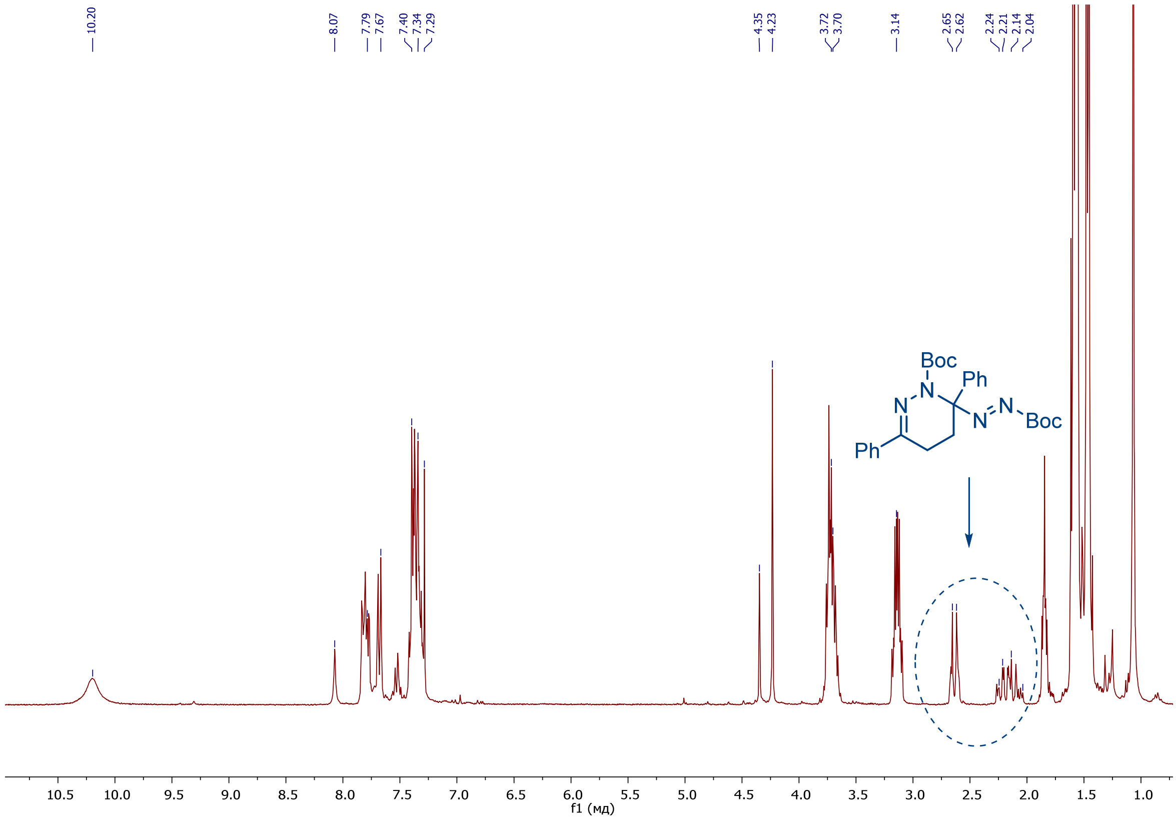

NMR for **AZA** dimer match with the literature data.[11]

[11] Kokuev, A. O.; Ioffe, S. L.; Sukhorukov, A. Yu. *Tetrahedron Lett.*, **2021**, *83*, 153414.

HRMS of the reaction mixture

Acquisition Parameter

Source Type	ESI	Ion Polarity	Positive	Set Nebulizer	0.4 Bar
Focus	Not active			Set Dry Heater	180 °C
Scan Begin	50 m/z	Set Capillary	4500 V	Set Dry Gas	4.0 l/min
Scan End	2500 m/z	Set End Plate Offset	-500 V	Set Divert Valve	Waste

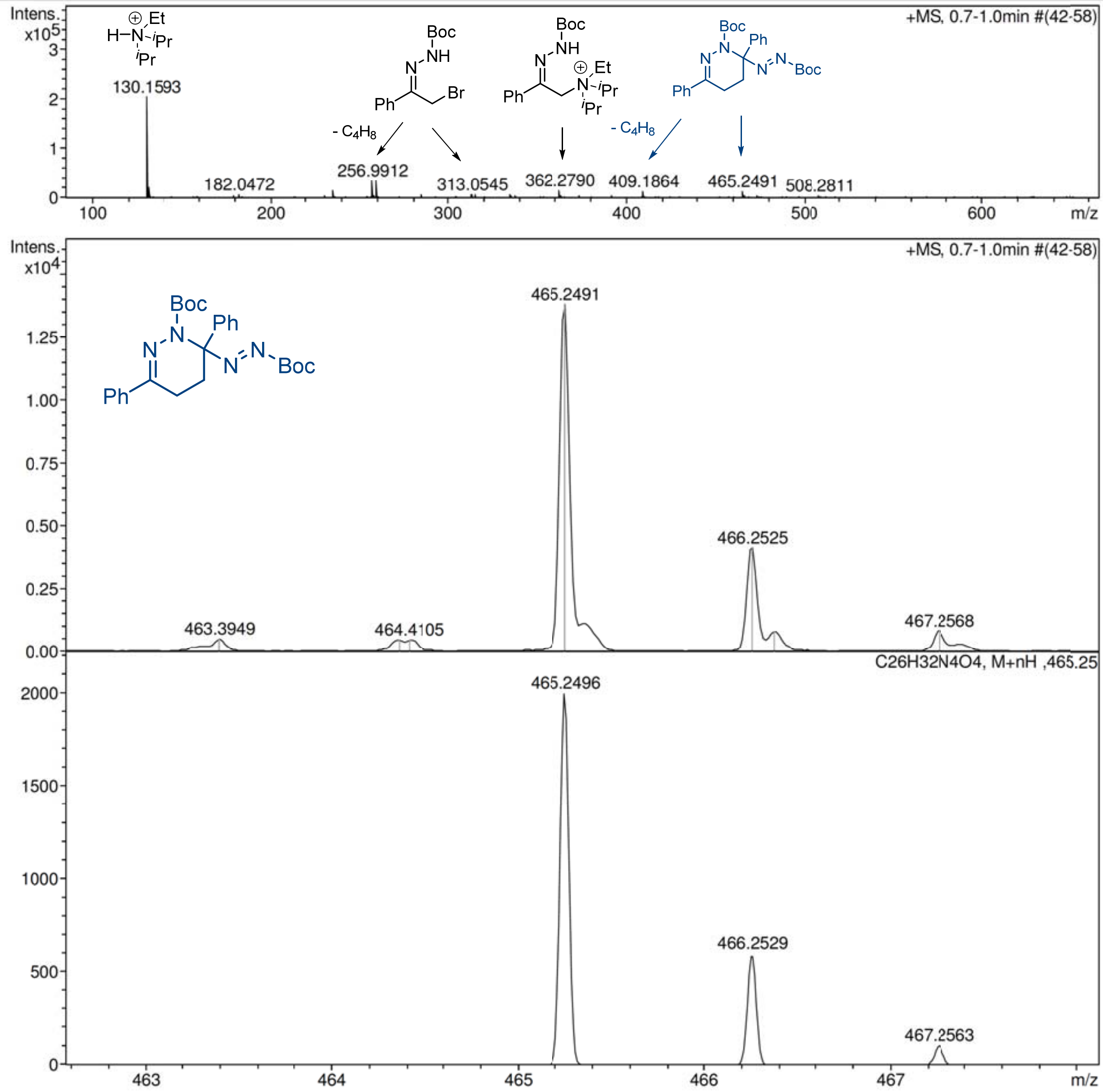

Copies of NMR and FT-IR spectra

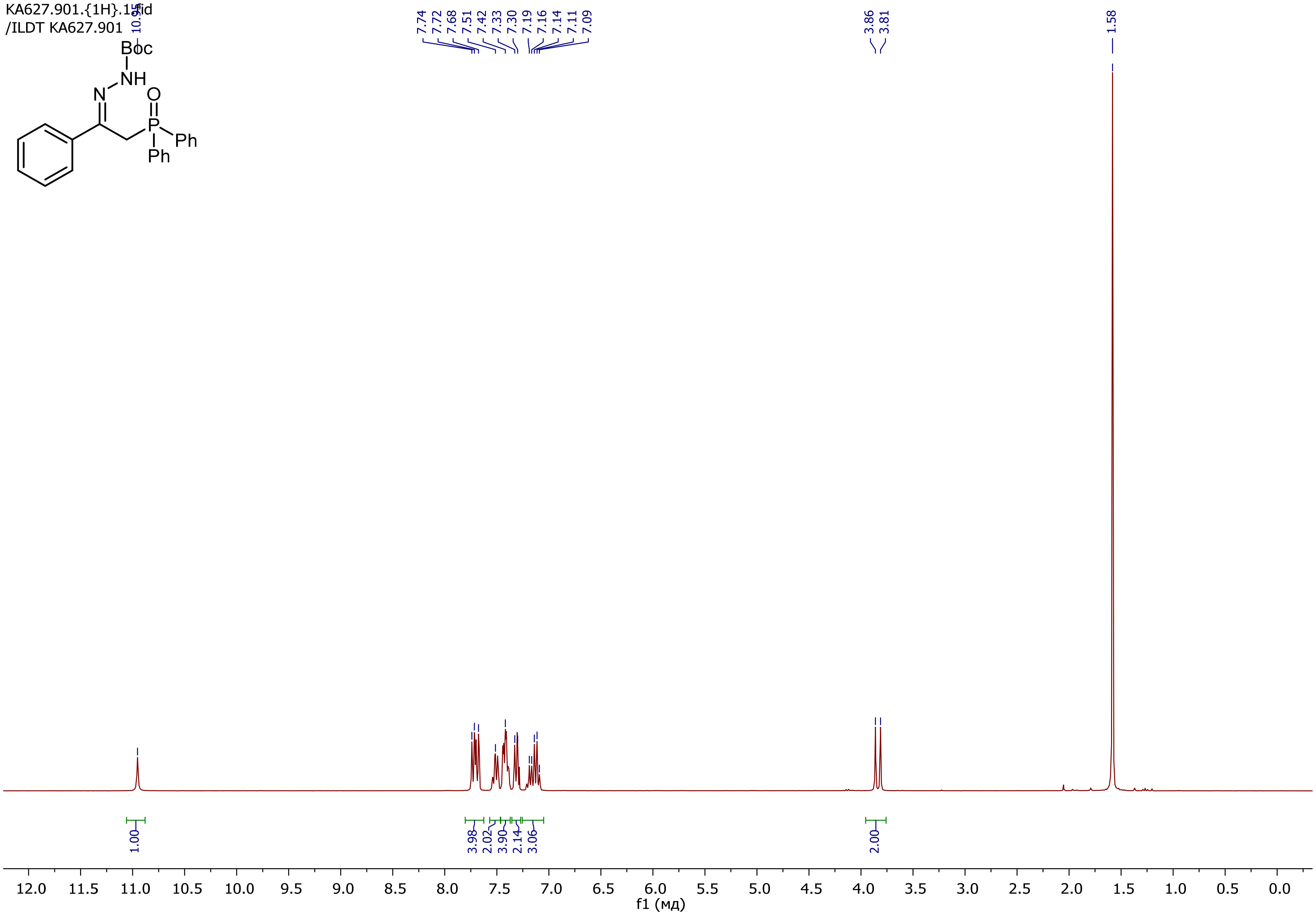

KA562.101.{1H}.1.fid
/ILDT KA562.101

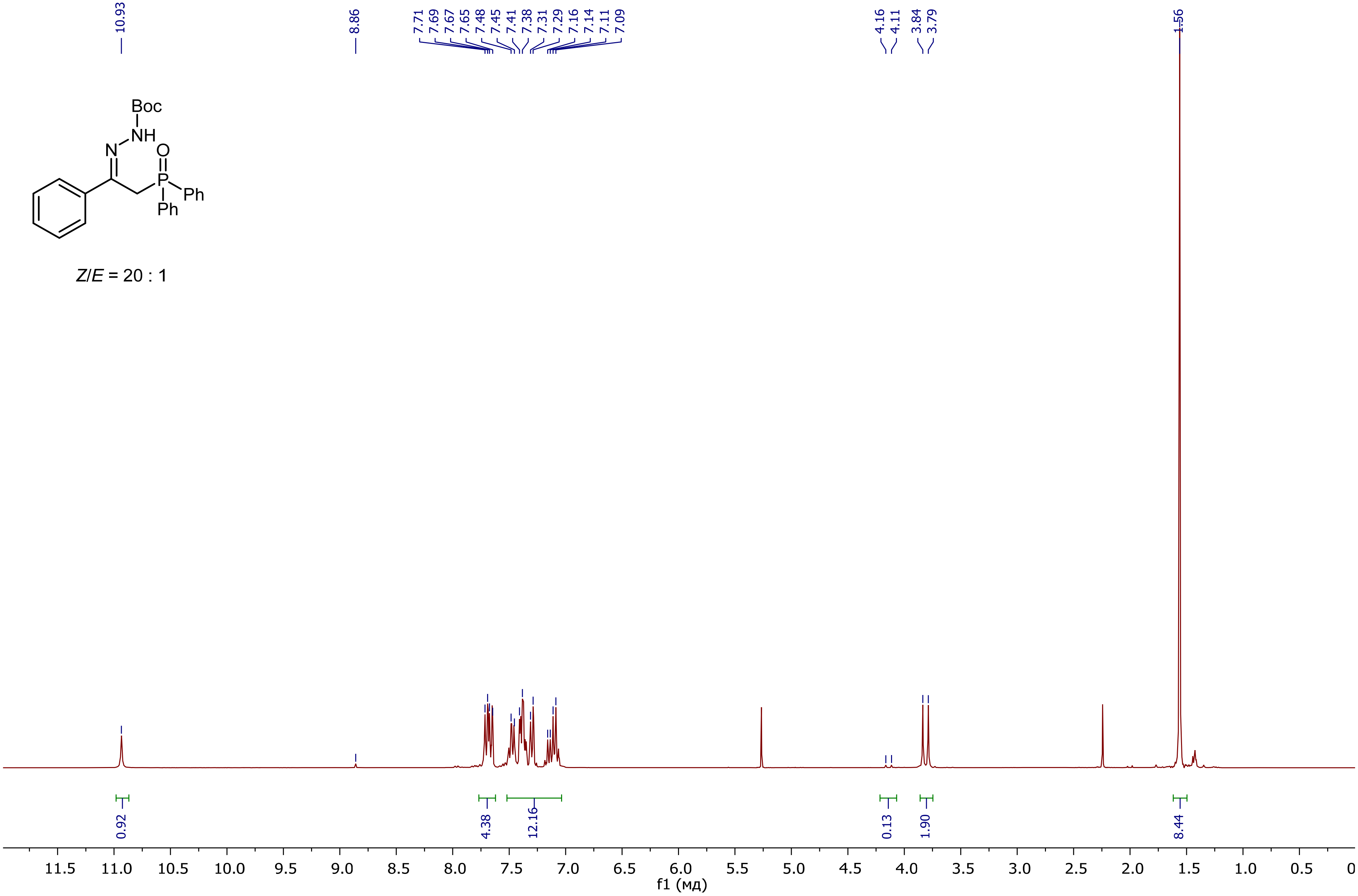

Z/*E* = 20 : 1

KA522.901.{13C}.2.fid
/ILDT KA522.901

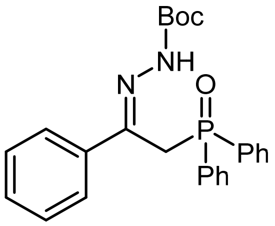

Z/*E* = 20 : 1

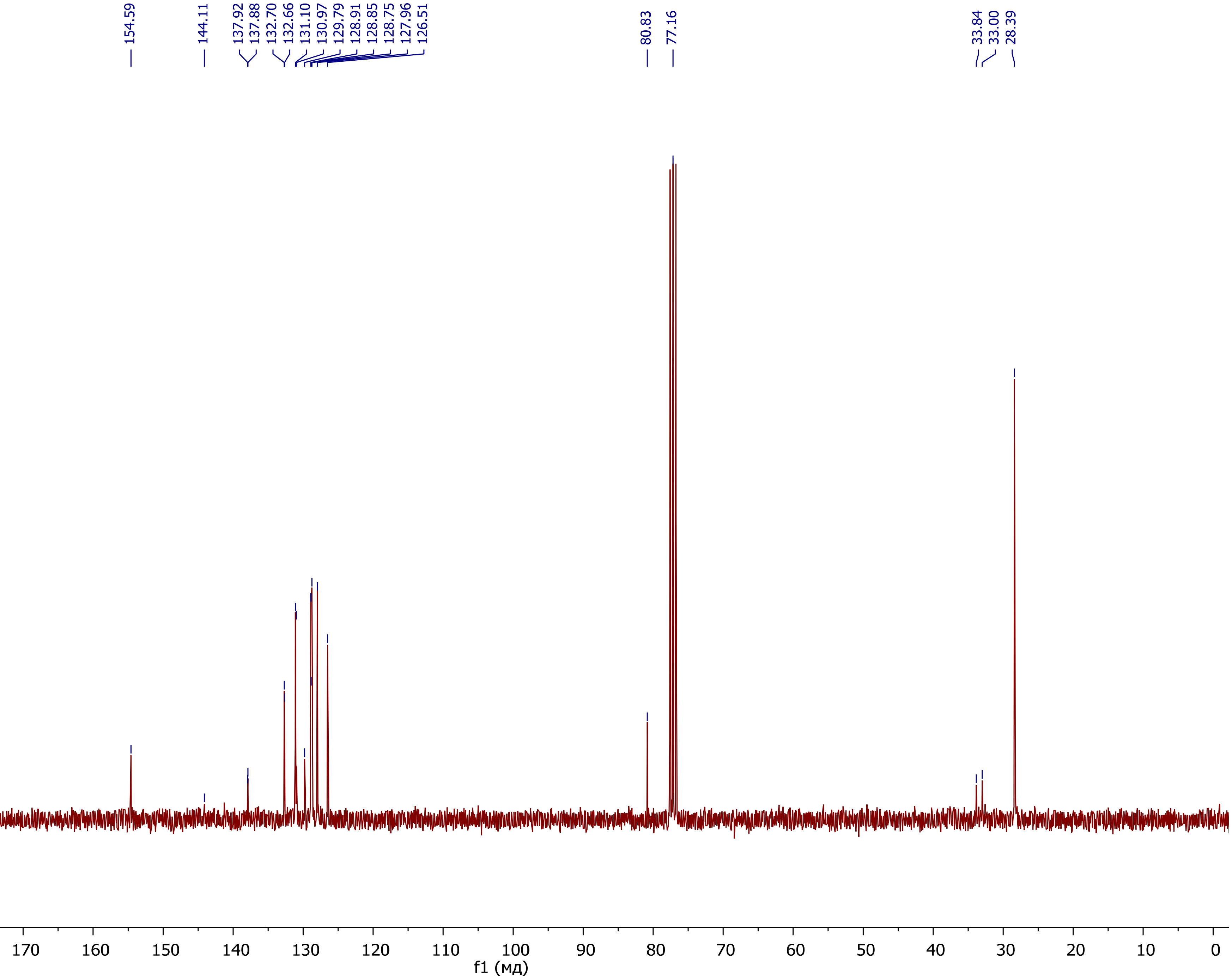

KA522.901.{13C}deptsp135.3.fid
/ILDT KA522.901

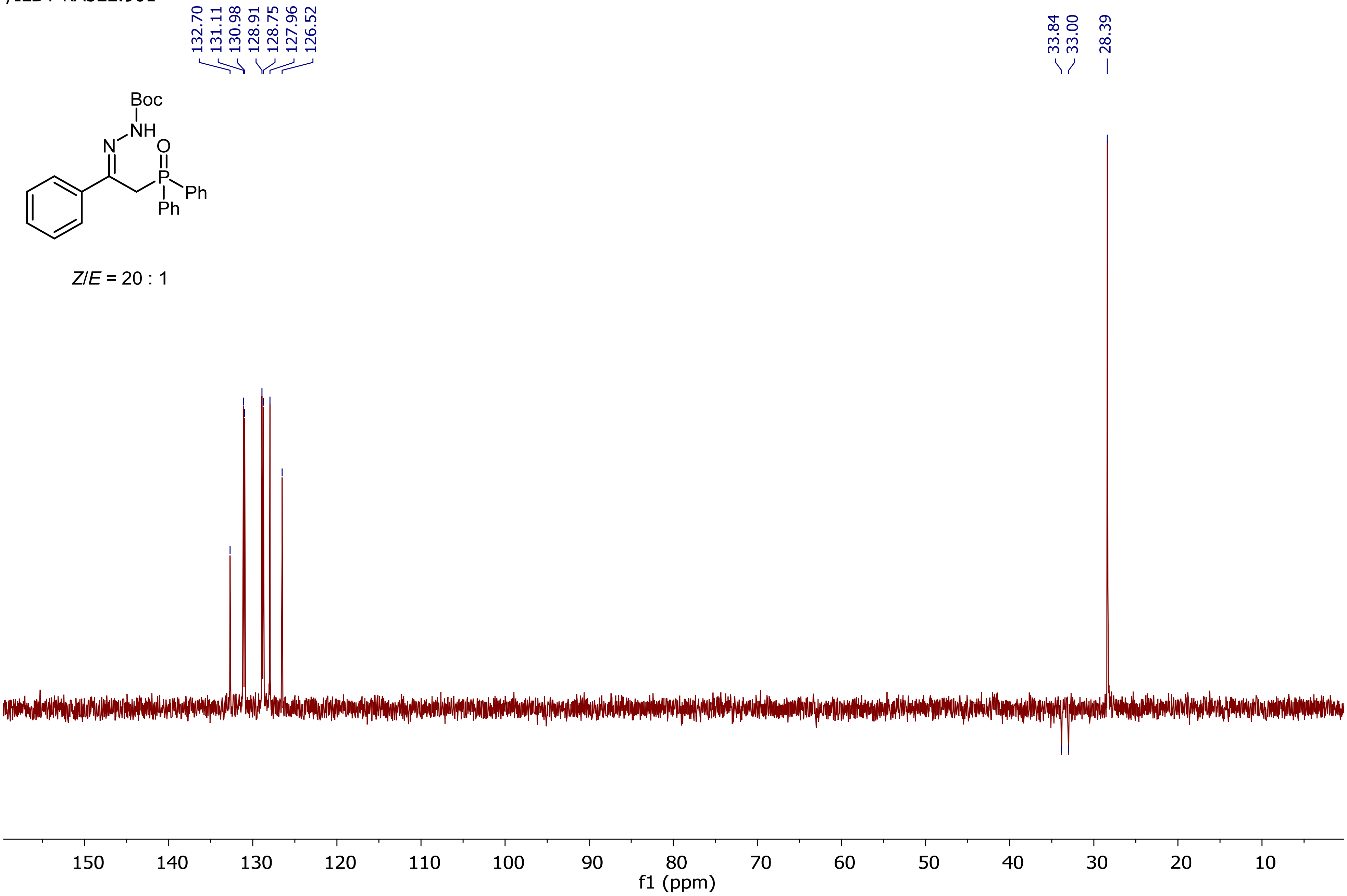

KA522.901.{31P}INVGATED.4.fid
/ILDT KA522.901

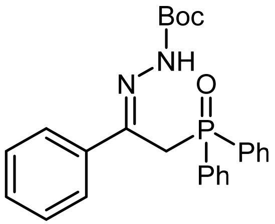

Z/*E* = 20 : 1

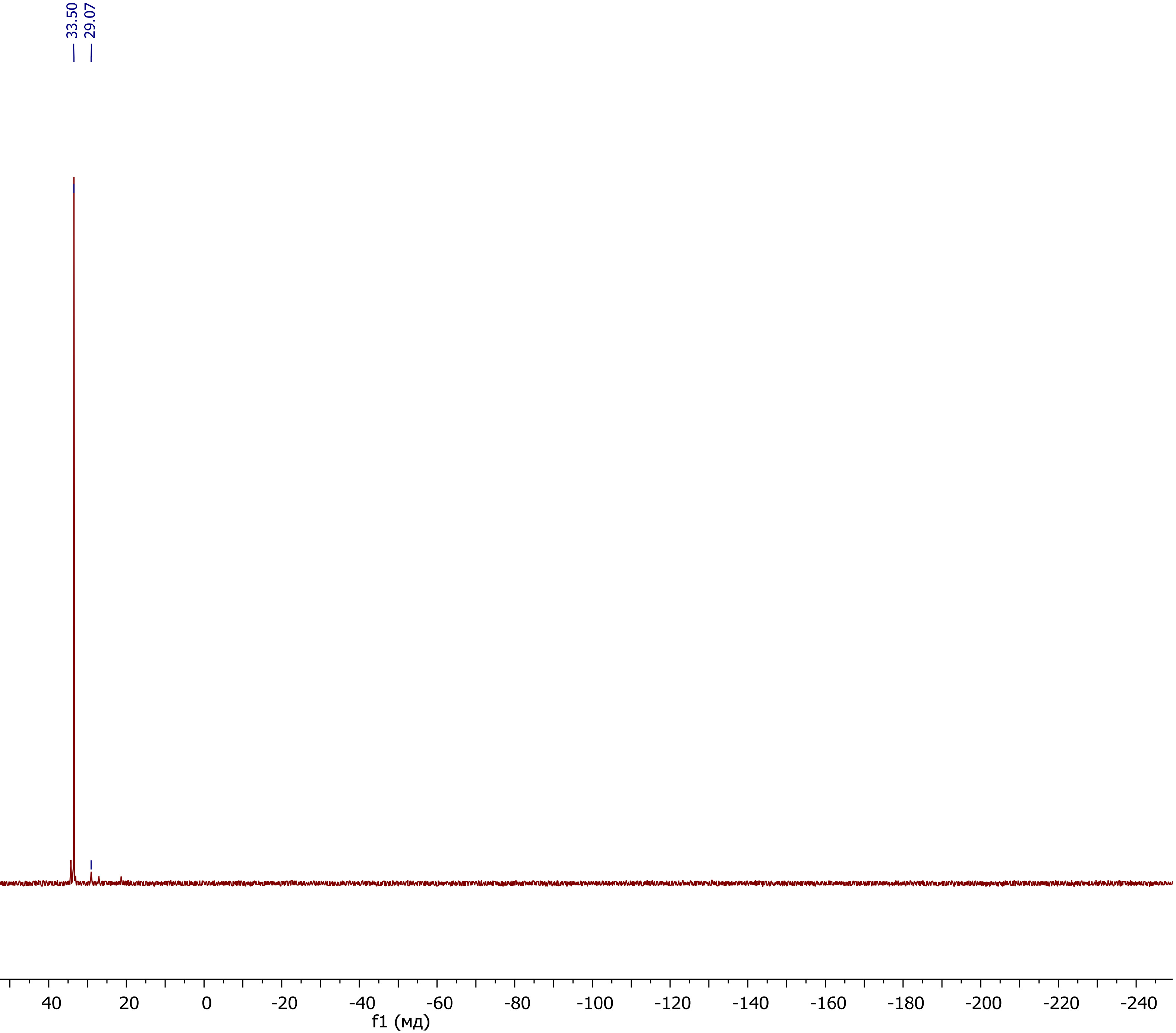

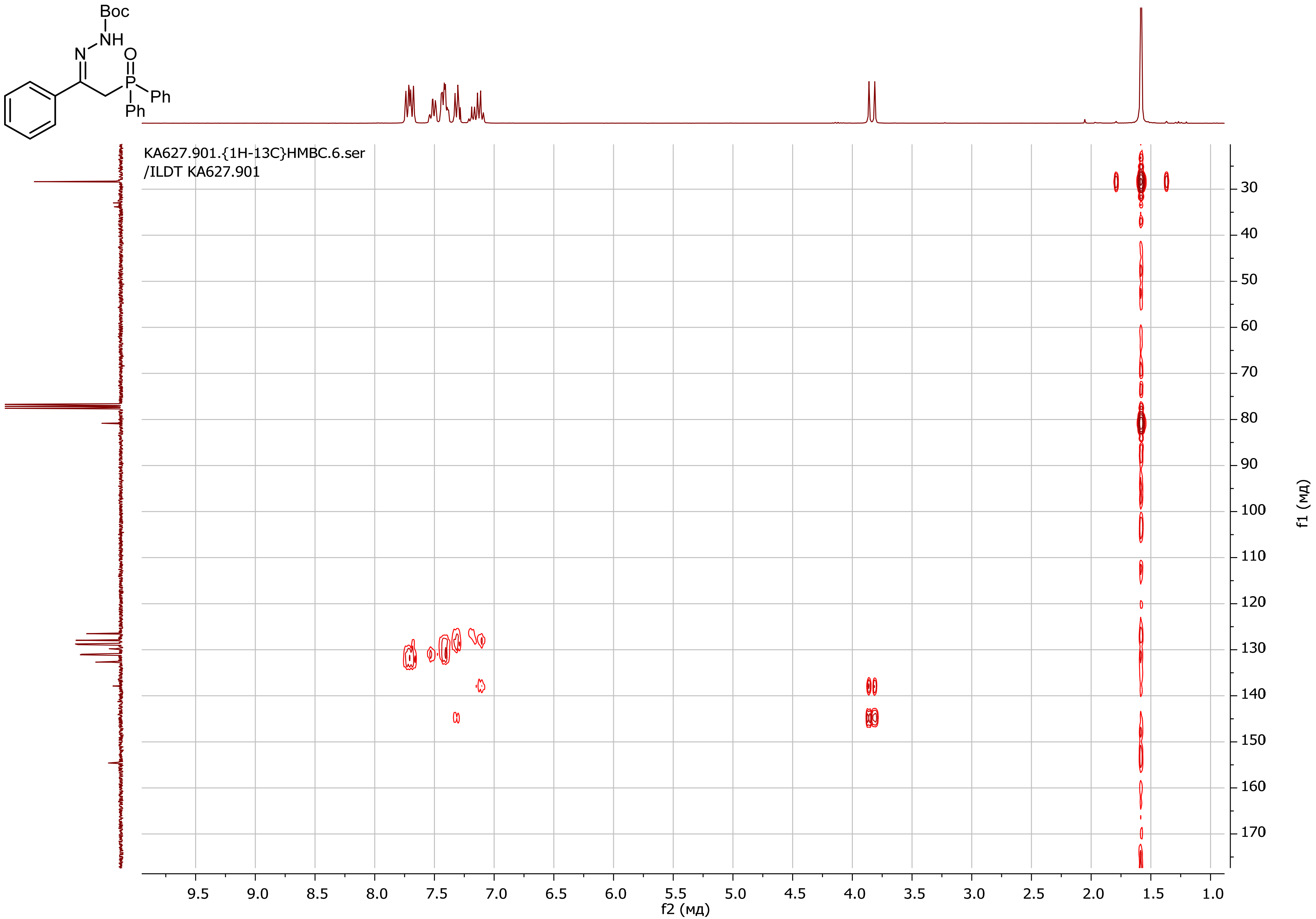
Boc
NH
N
O
P
Ph
Ph
KA627.901.{1H-13C}HMBC.6.ser
/ILDT KA627.901
f2 (мд)
f1 (мд)

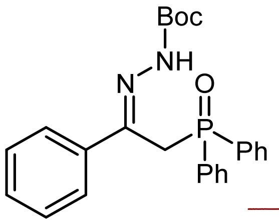
Boc
NH
N
O
P
Ph
Ph

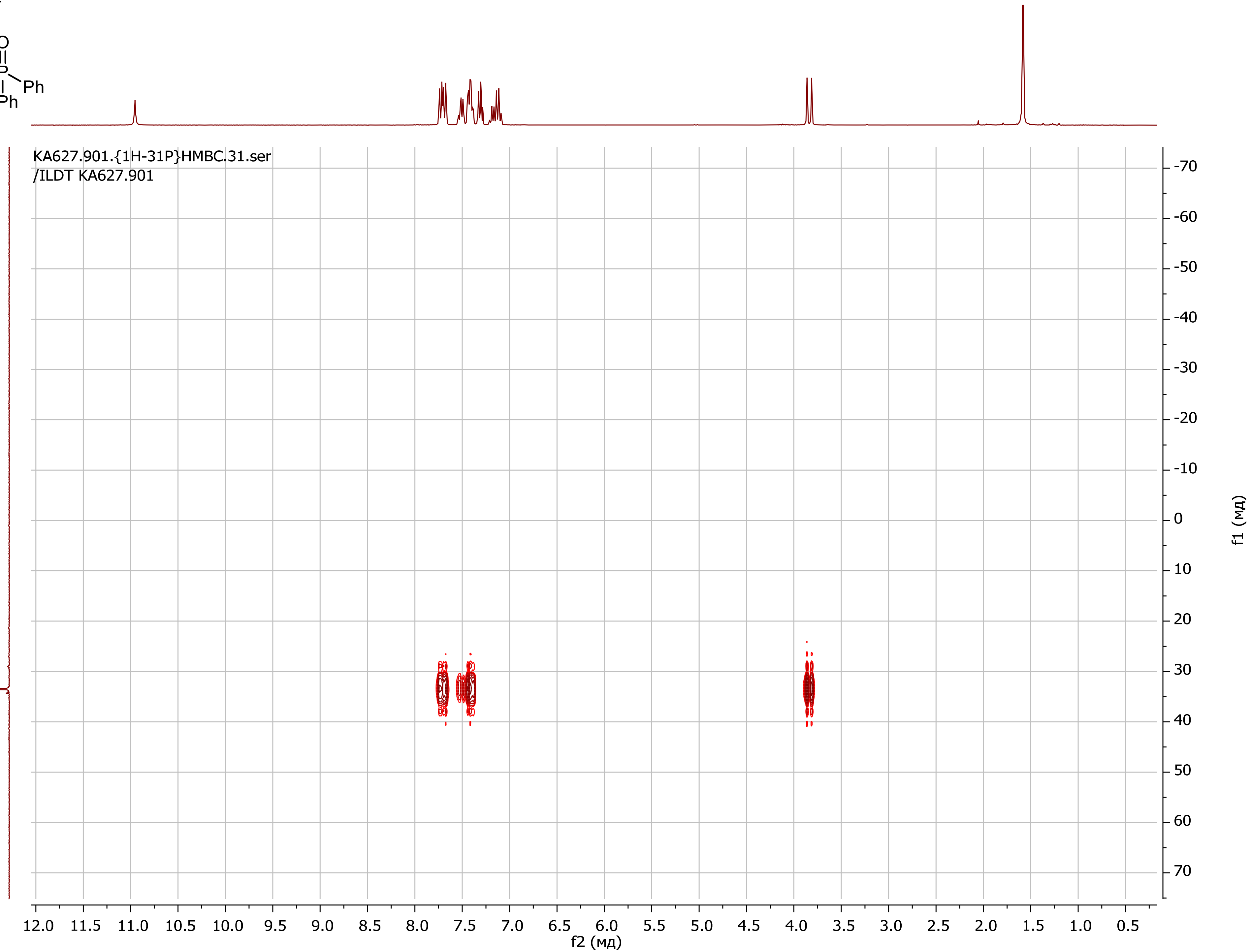
KA627.901.{1H-31P}HMBC.31.ser
/ILDT KA627.901
f2 (мд)
f1 (мд)

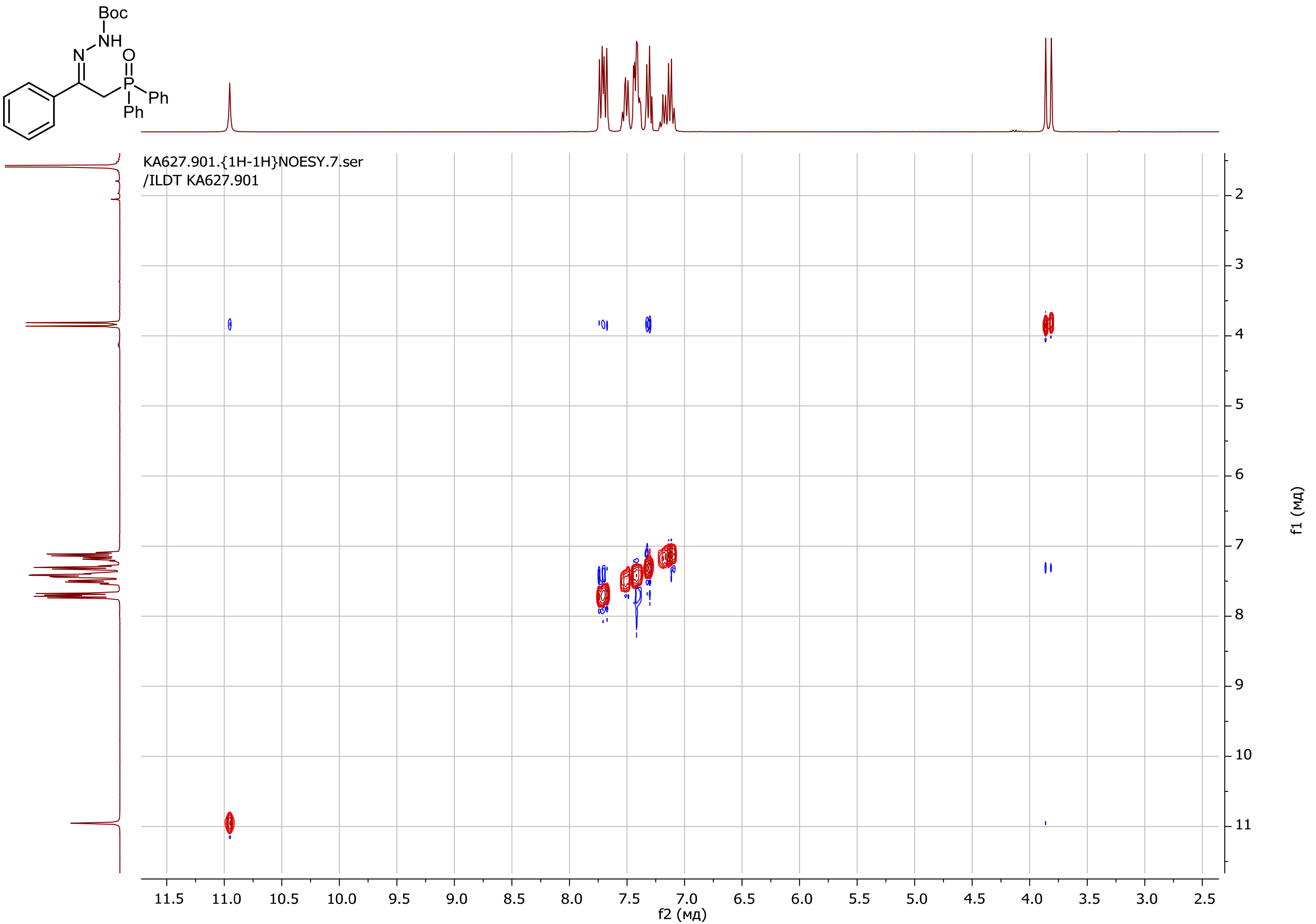

Boc
NH
N
O
P
Ph
Ph
KA627.901.{1H-1H}NOESY.7.ser
/ILDT KA627.901
f2 (мд)
f1 (мд)

KA564.701.{1H}.1.fid
/ILDT KA564.701

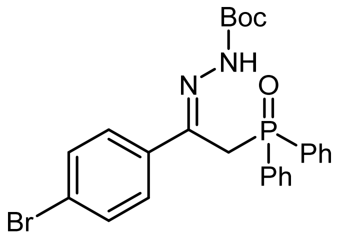

Z/*E* = 9 : 1

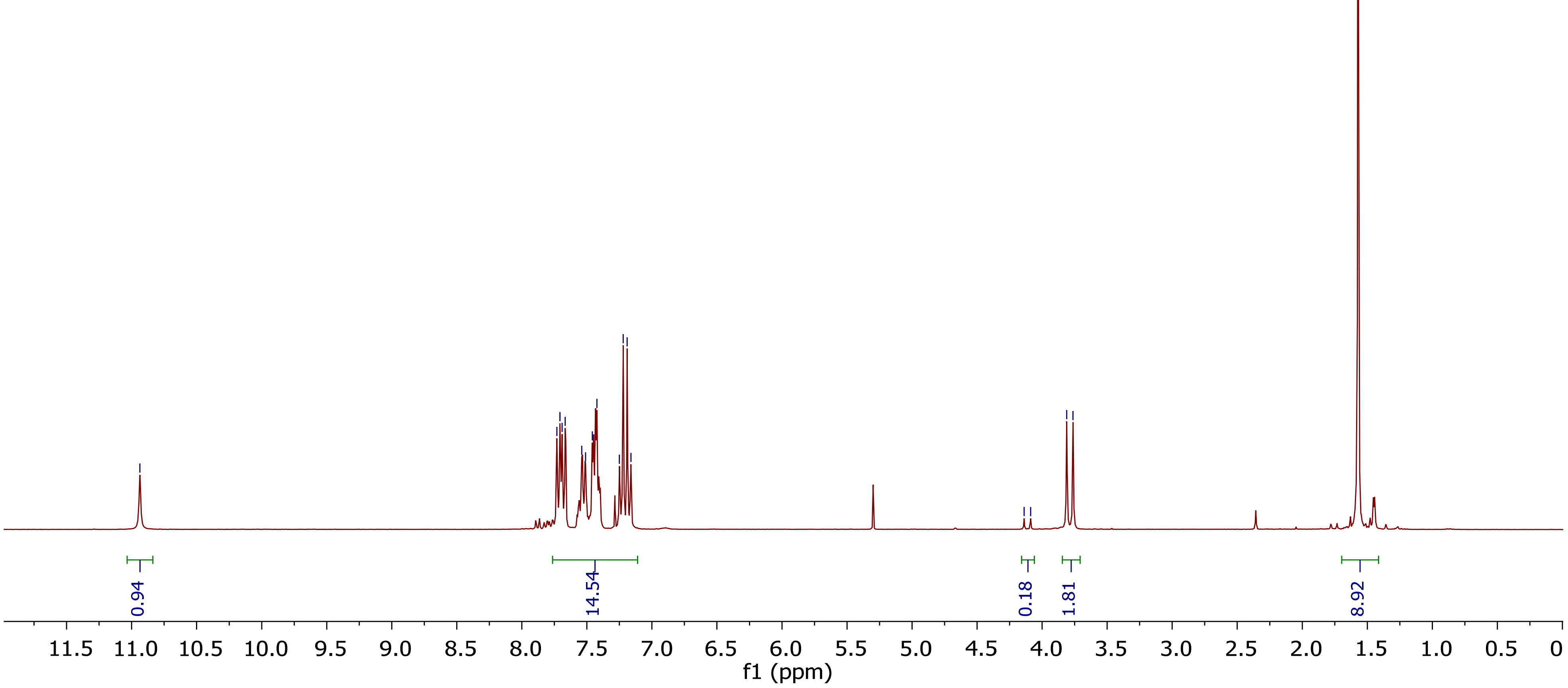

KA564.701.{13C}.2.fid
/ILDT KA564.701

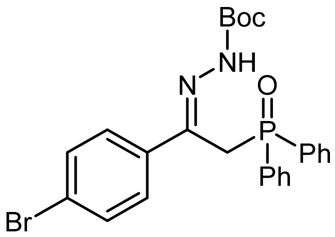

Z/*E* = 9 : 1

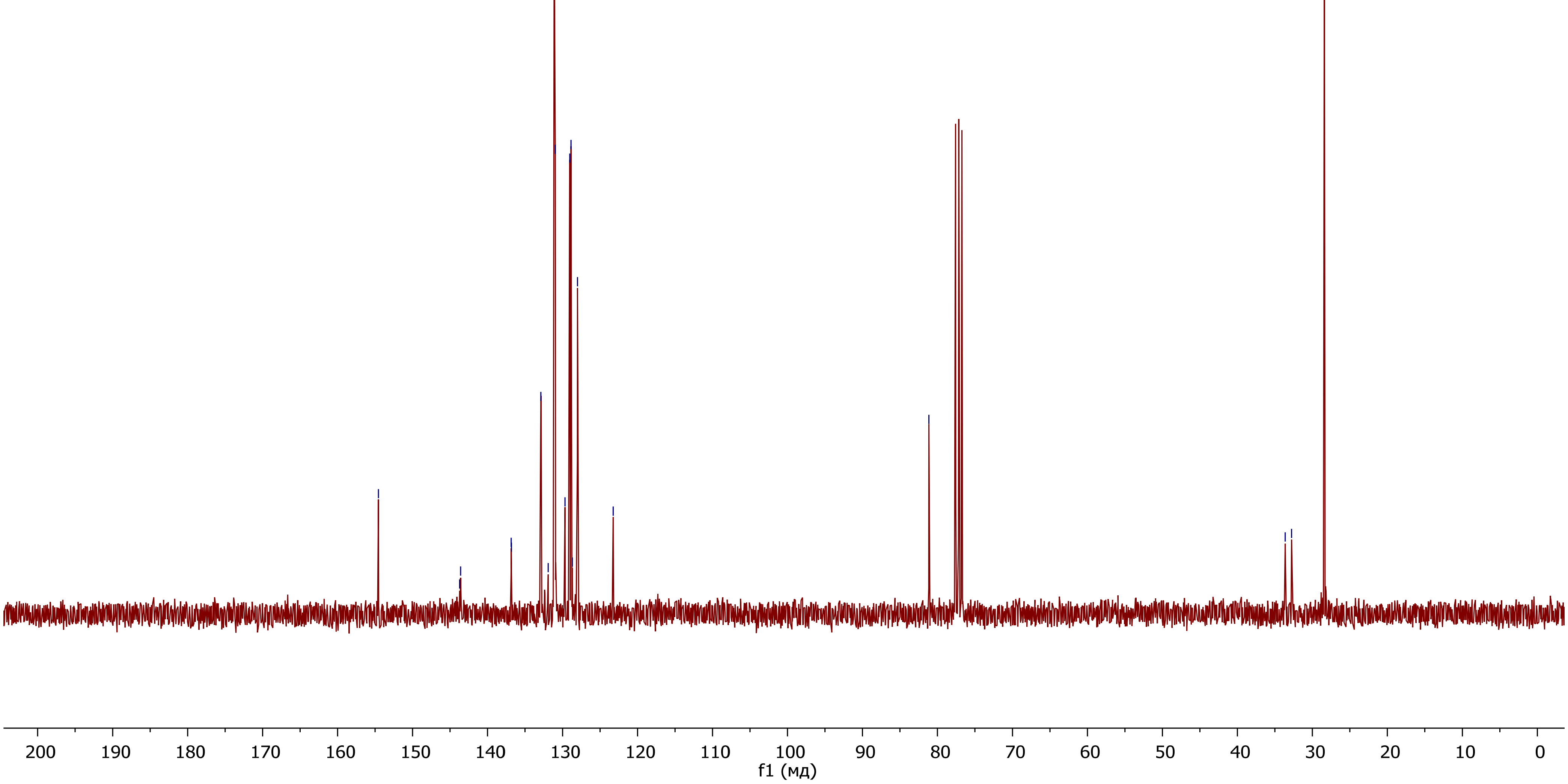

KA564.701.{13C}deptsp135.3.fid
/ILDT KA564.701

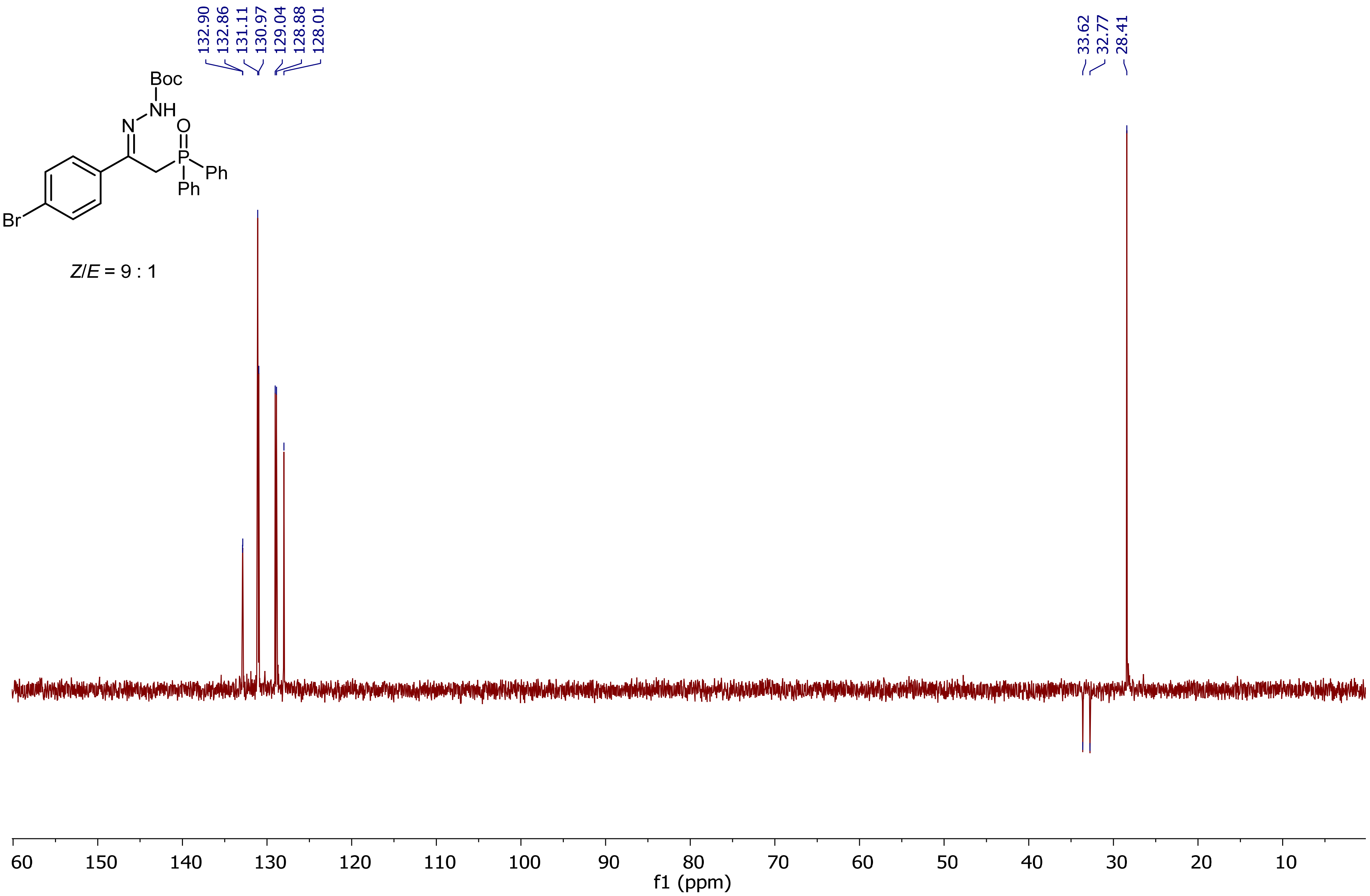

KA564.701.{31P}INVGATED.31.fid
/ILDT KA564.701

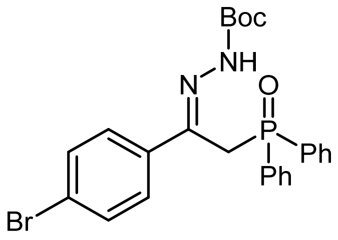

Z/*E* = 9 : 1

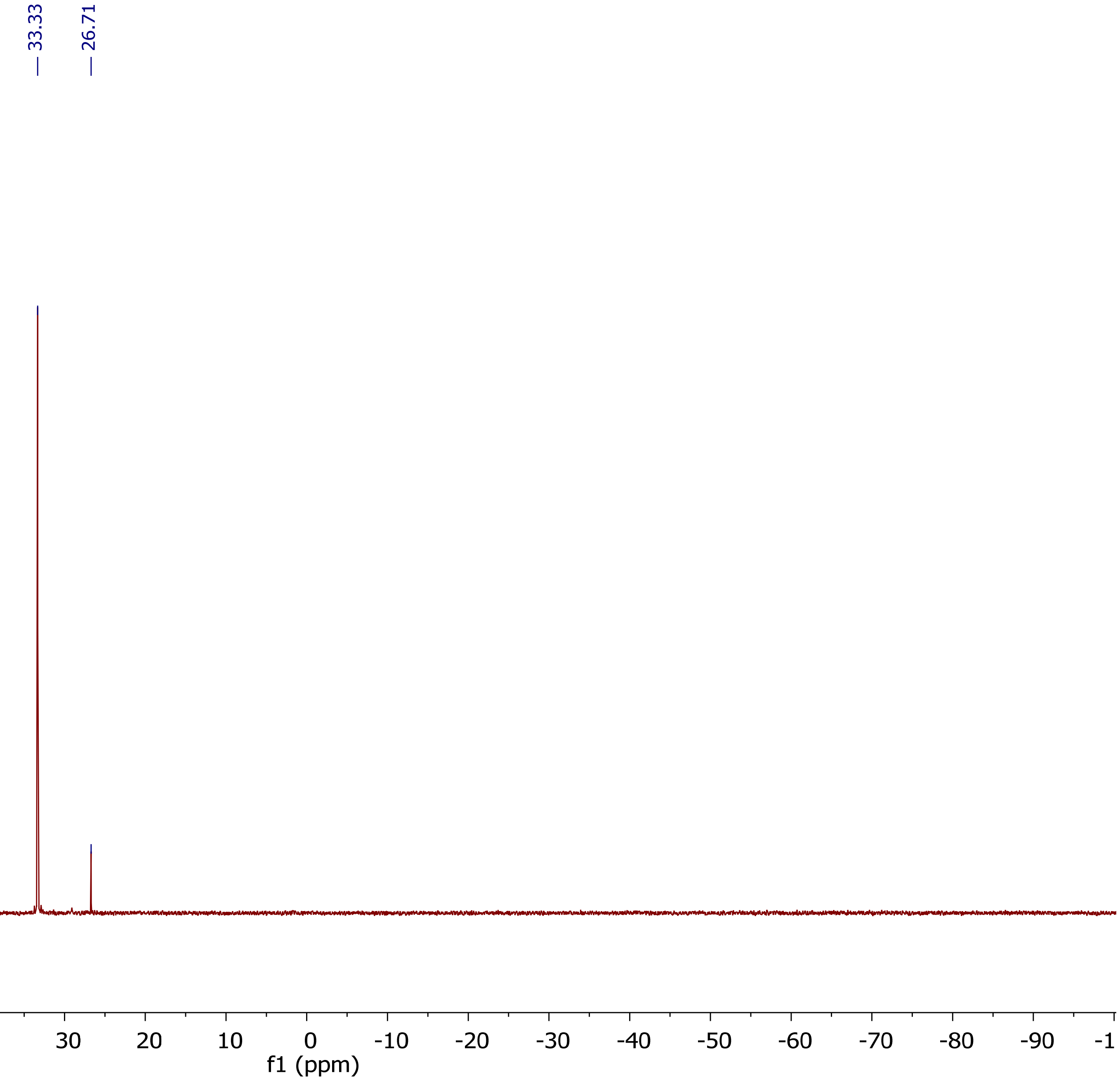

KA565.701.{1H}.1.fid
/ILDT KA565.701

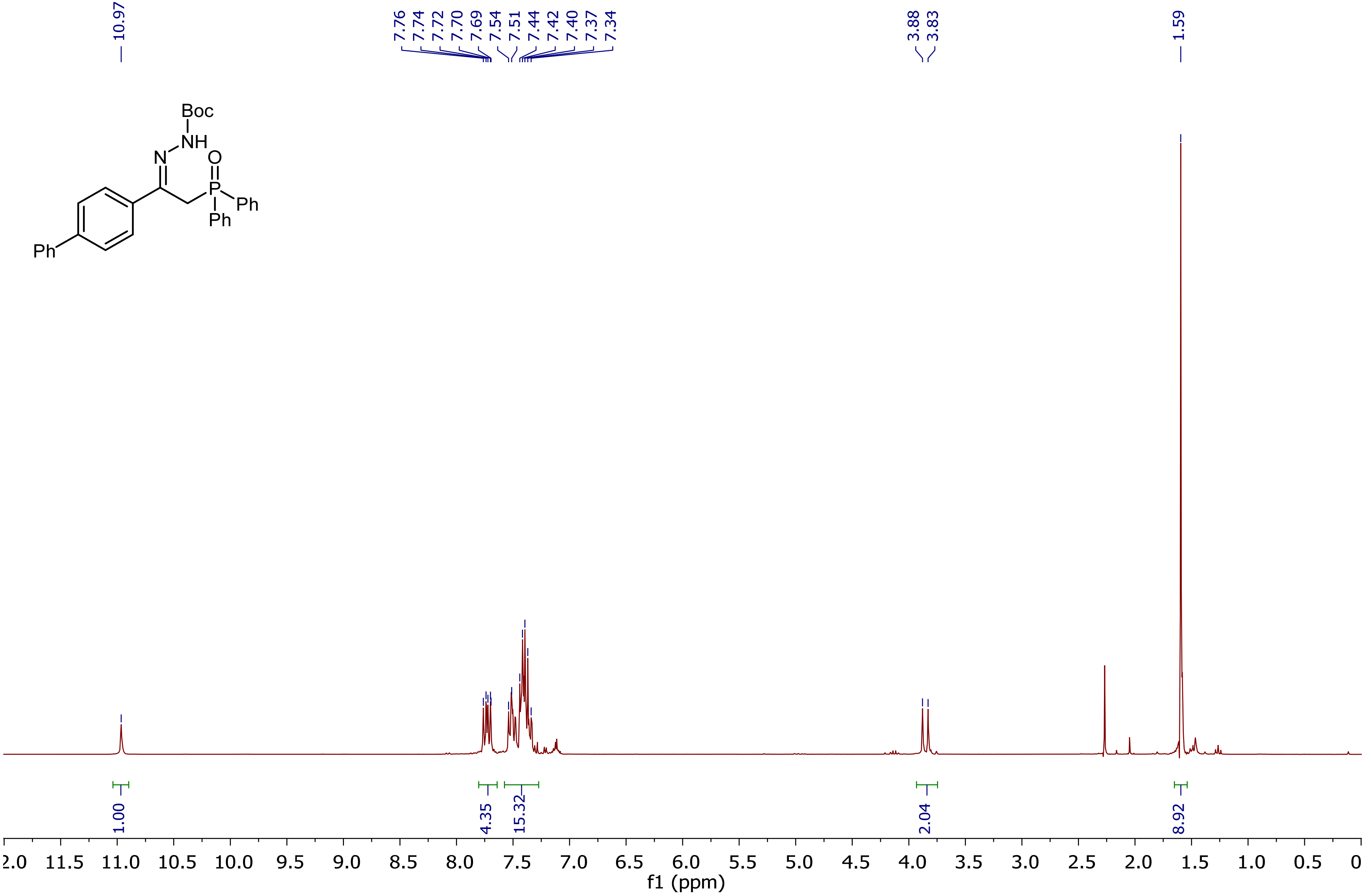

KA565.701.{13C}.2.fid
/ILDT KA565.701

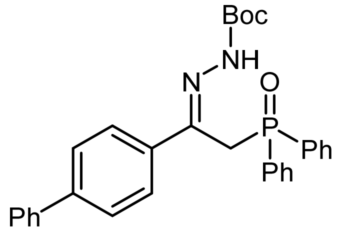

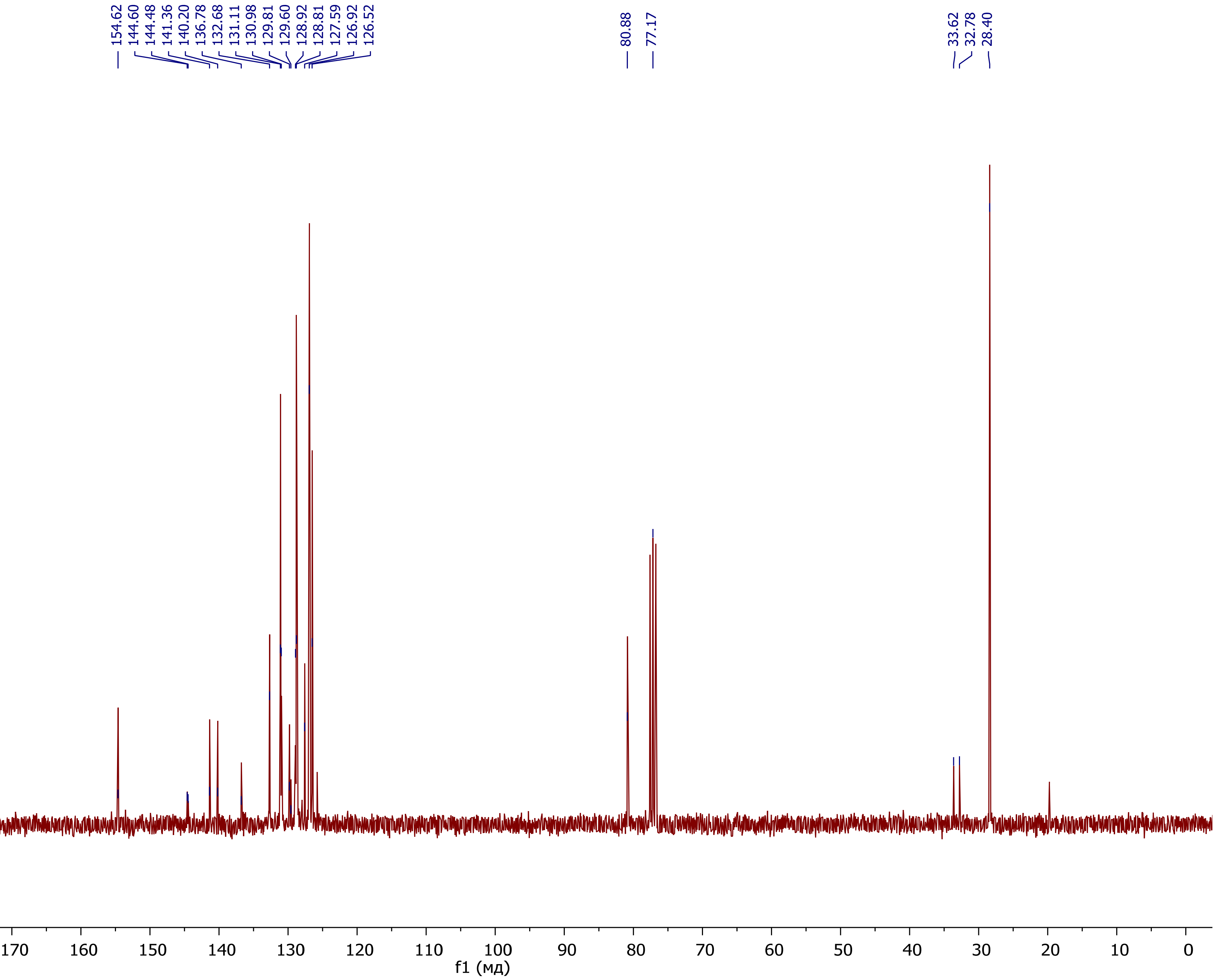

KA565.701.{13C}deptsp135.3.fid
/ILDT KA565.701

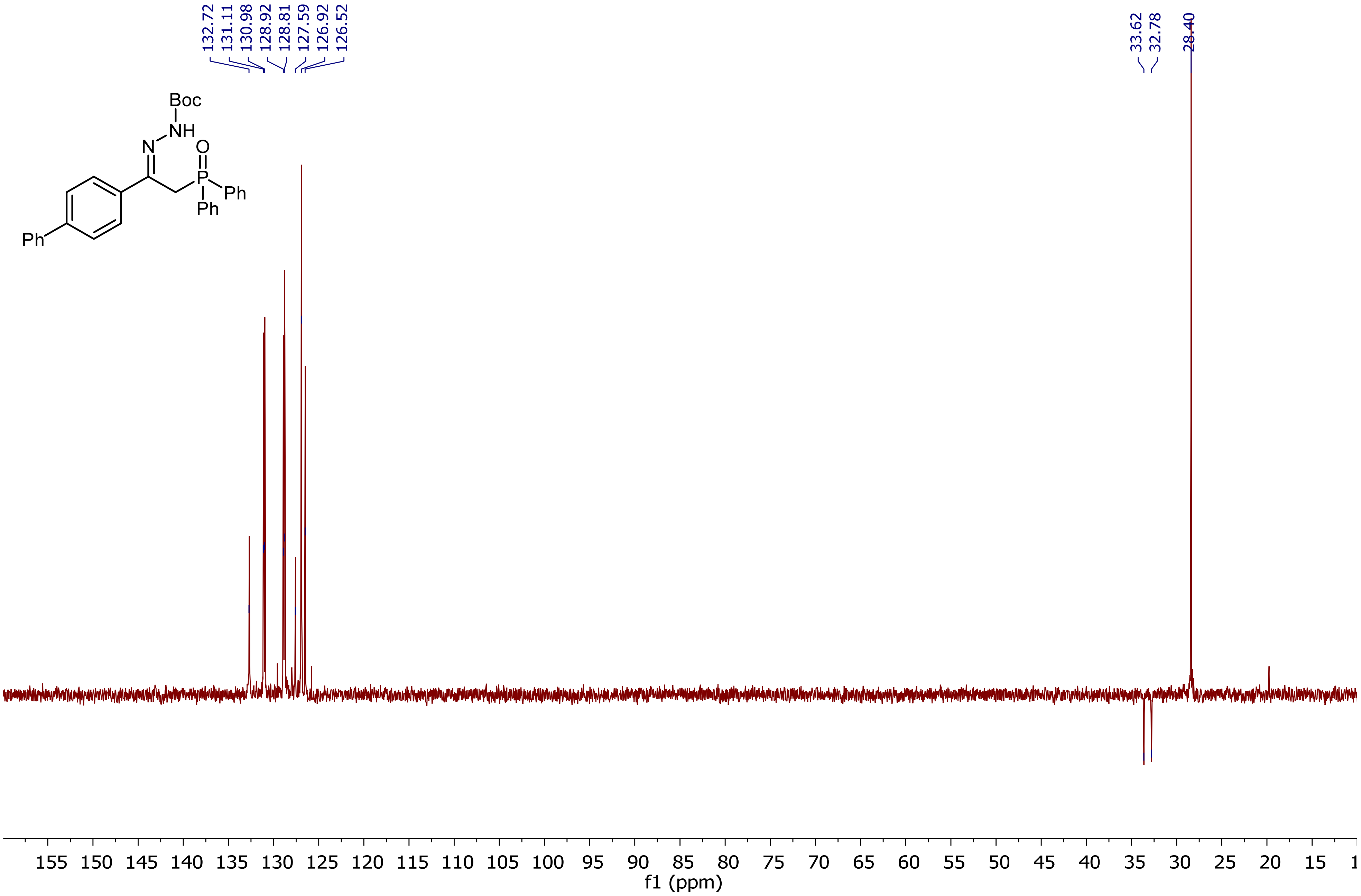

KA565.701.{31P}INVGATED.4.fid
/ILDT KA565.701

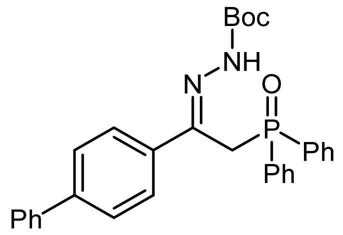

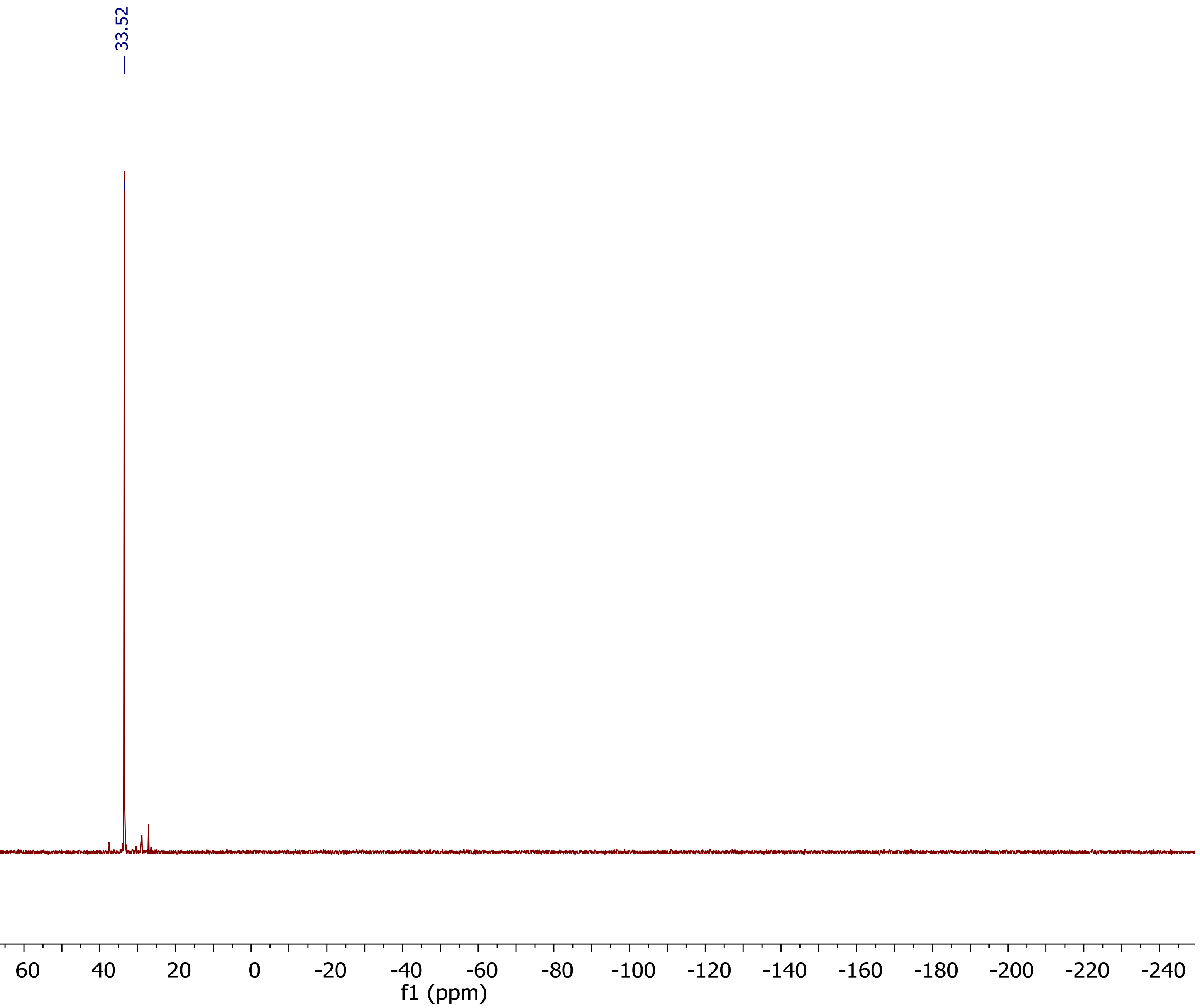

KA629.101.{1H}.1.fid
/ILDT KA629.101

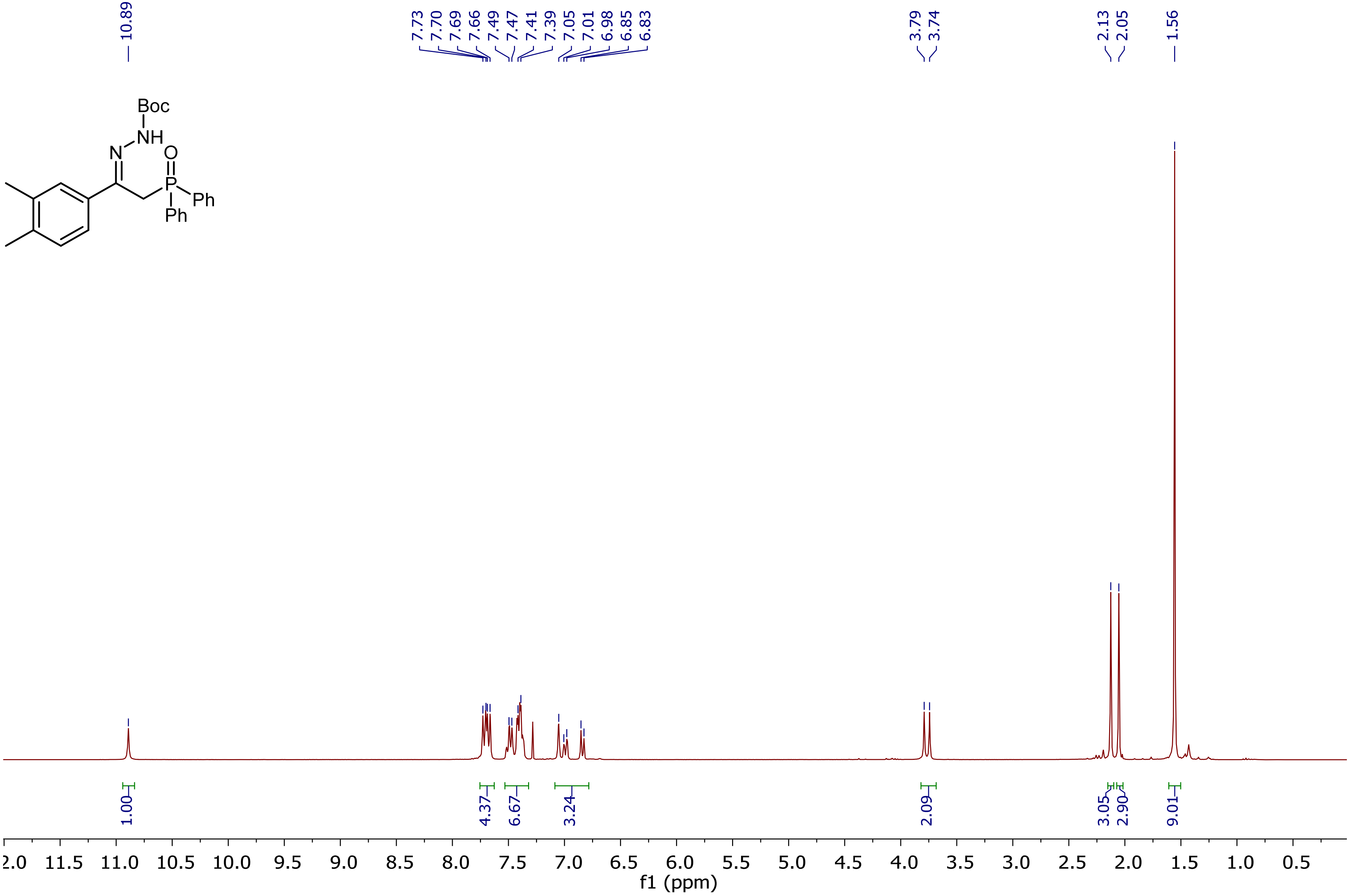

KA629.801.{13C}.2.fid
/ILDT KA629.801

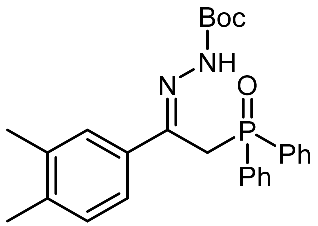

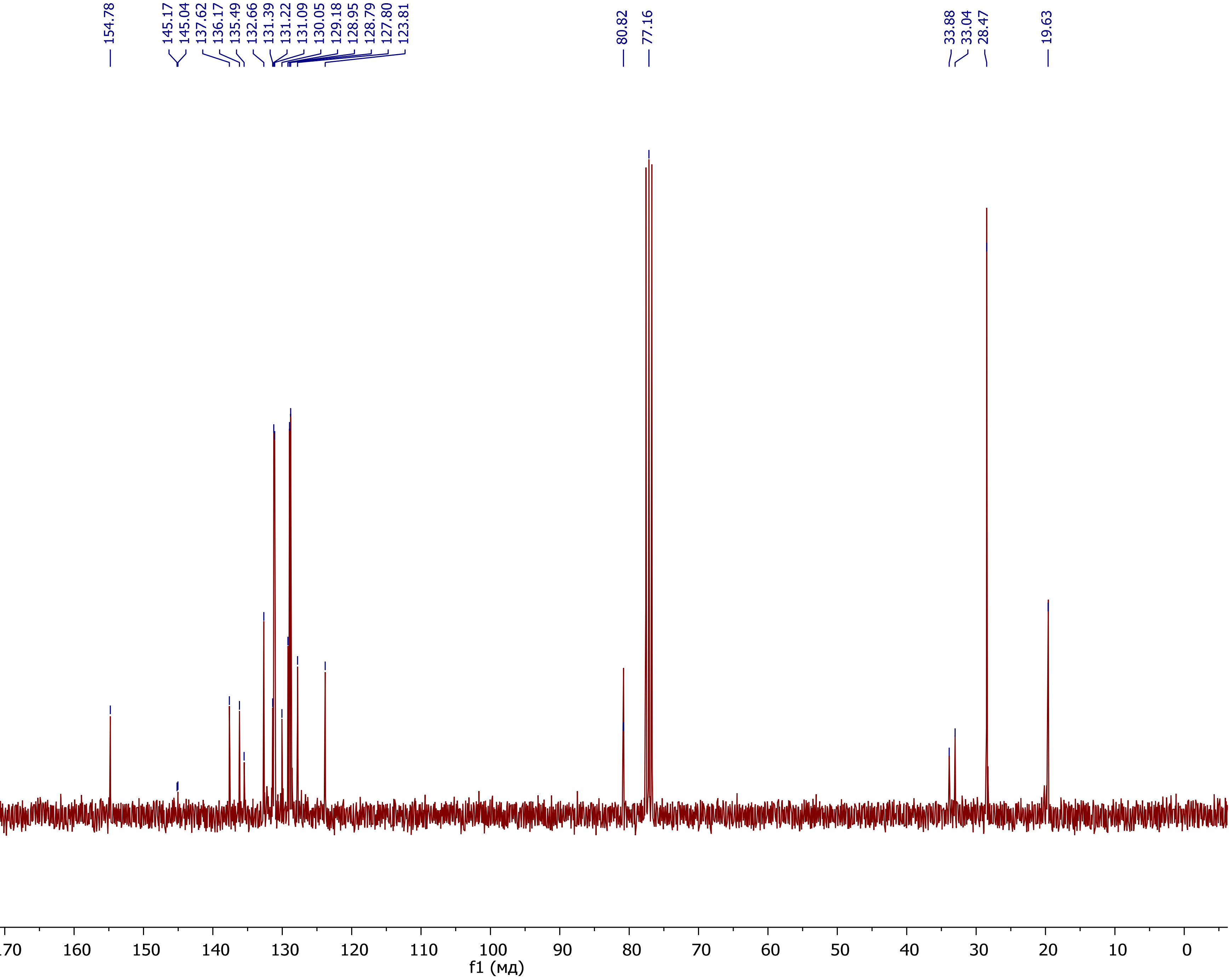

KA629.801.{13C}deptsp135.3.fid
/ILDT KA629.801

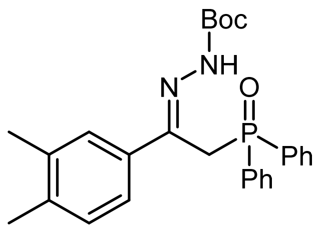

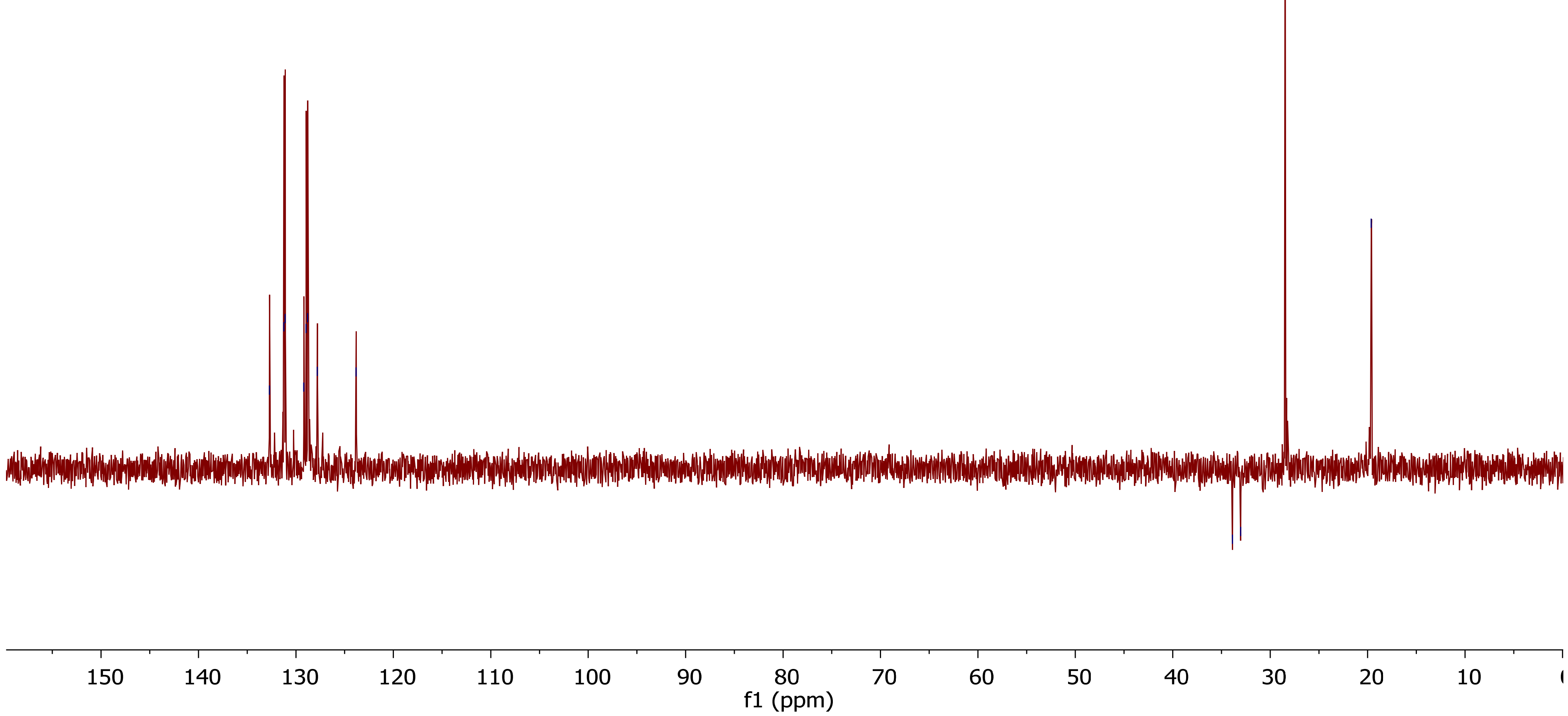

KA629.801.{31P}INVGATED.31.fid
/ILDT KA629.801

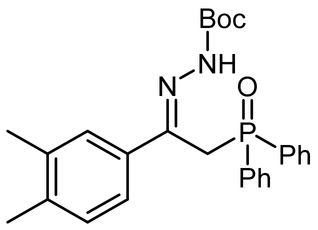

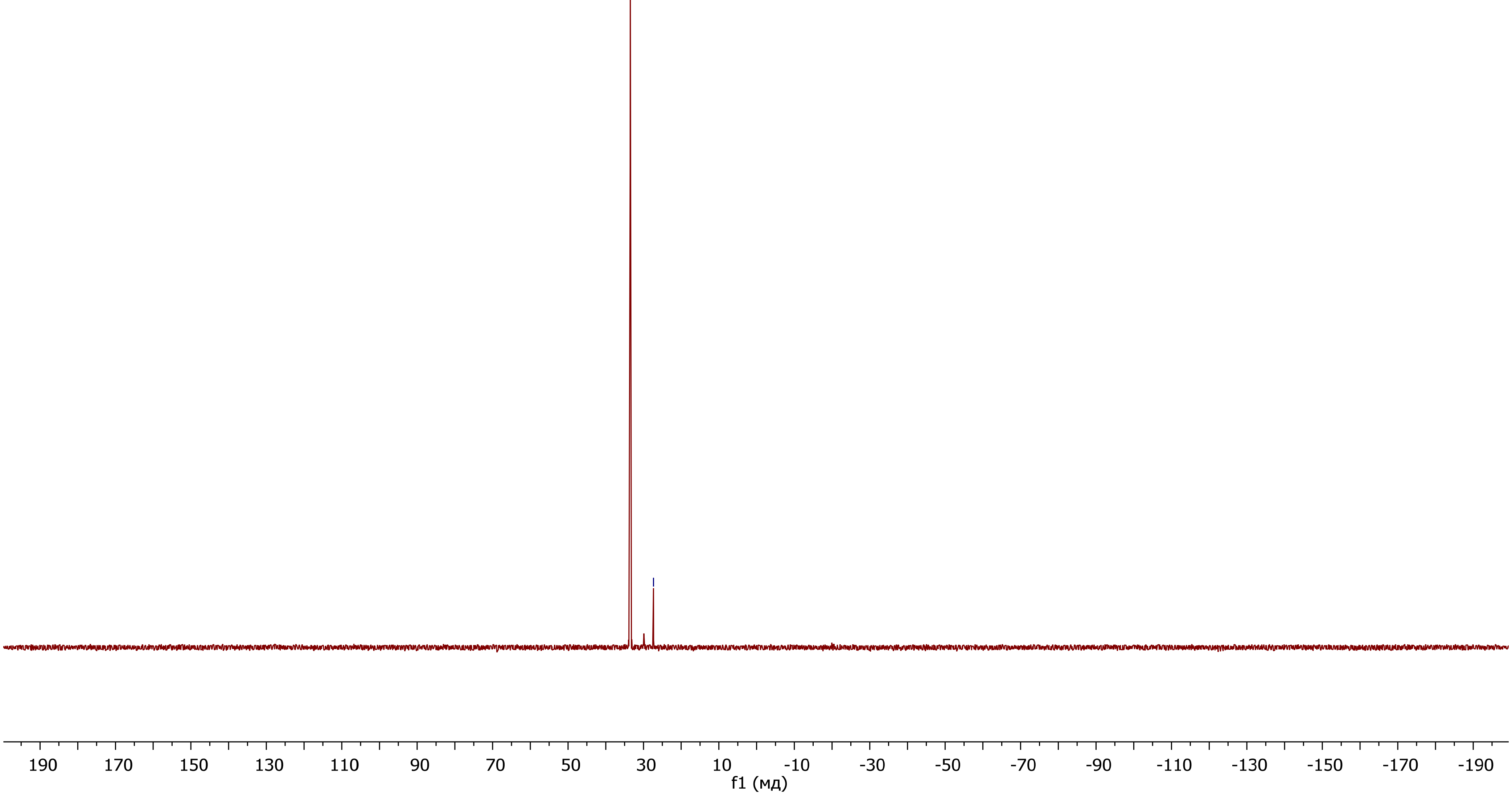

KA628.101.{1H}.1.fid
/ILDT KA628.101

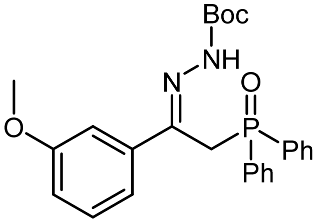

Z/*E* = 12 : 1

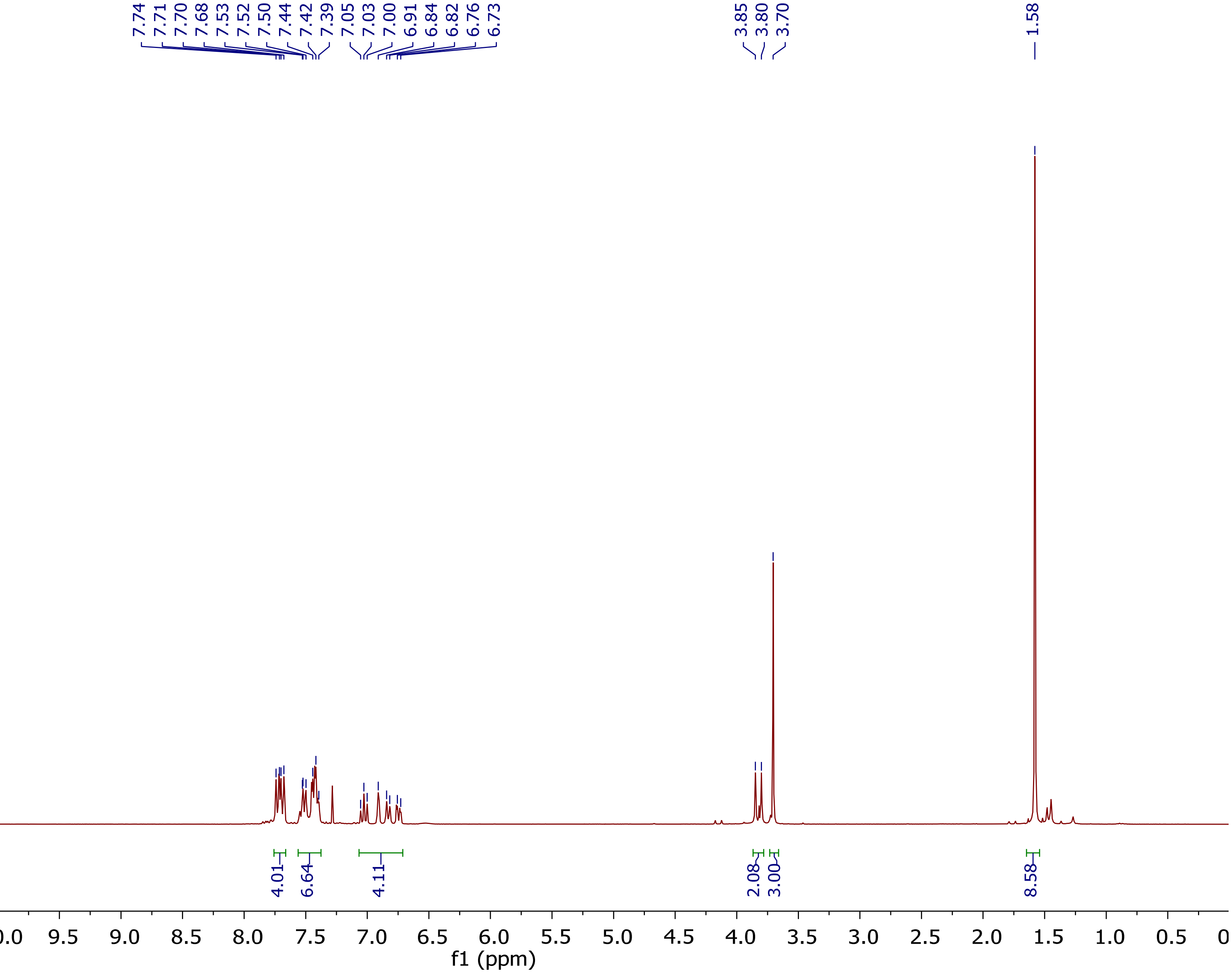

KA628.101.{13C}.2.fid
/ILDT KA628.101

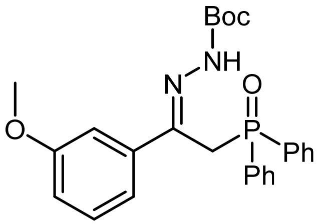

Z/*E* = 12 : 1

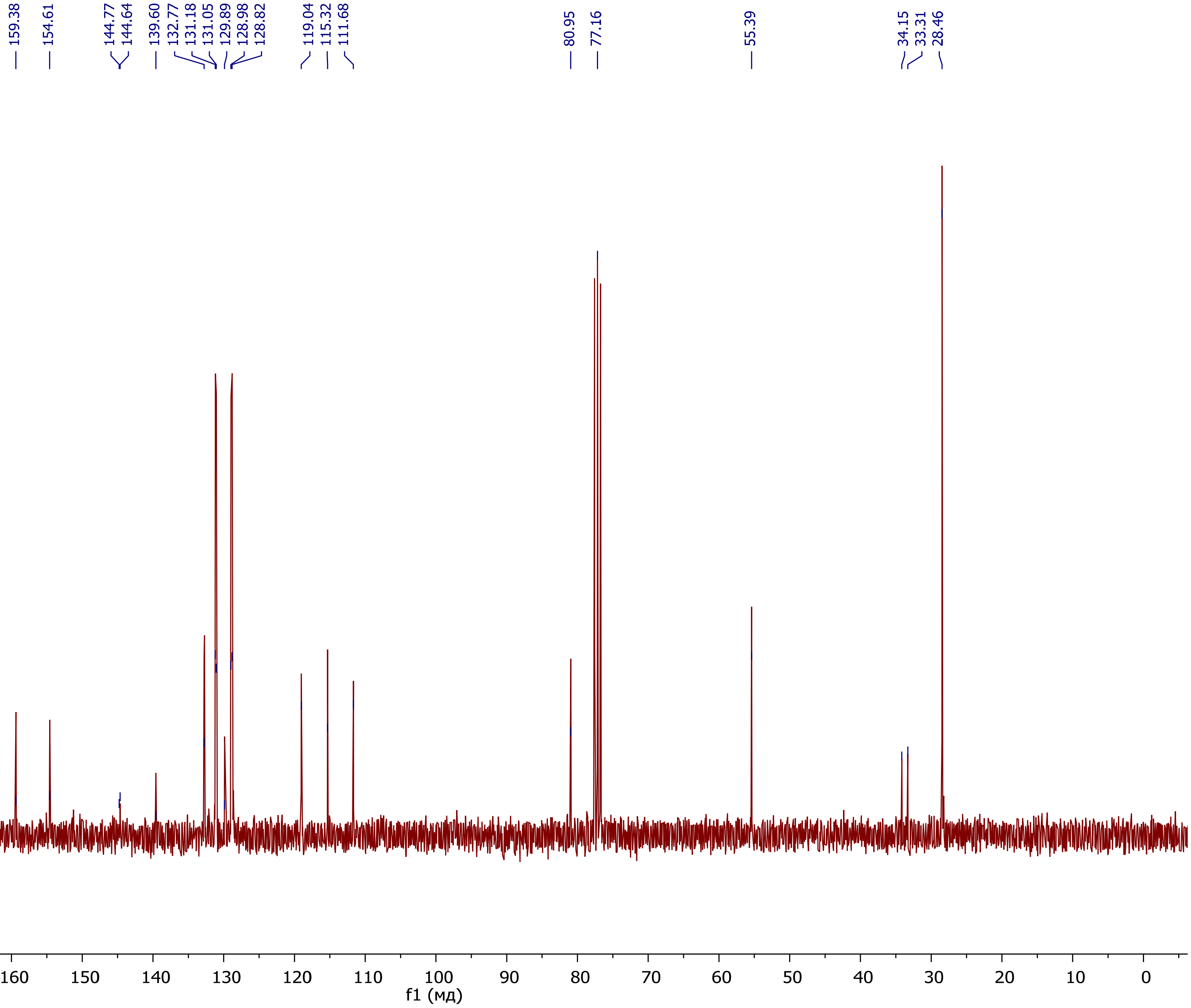

KA628.101.{13C}deptsp135.3.fid
/ILDT KA628.101

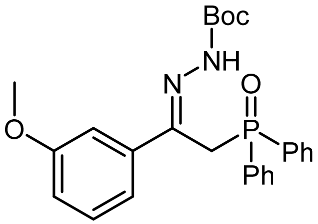

Z/*E* = 12 : 1

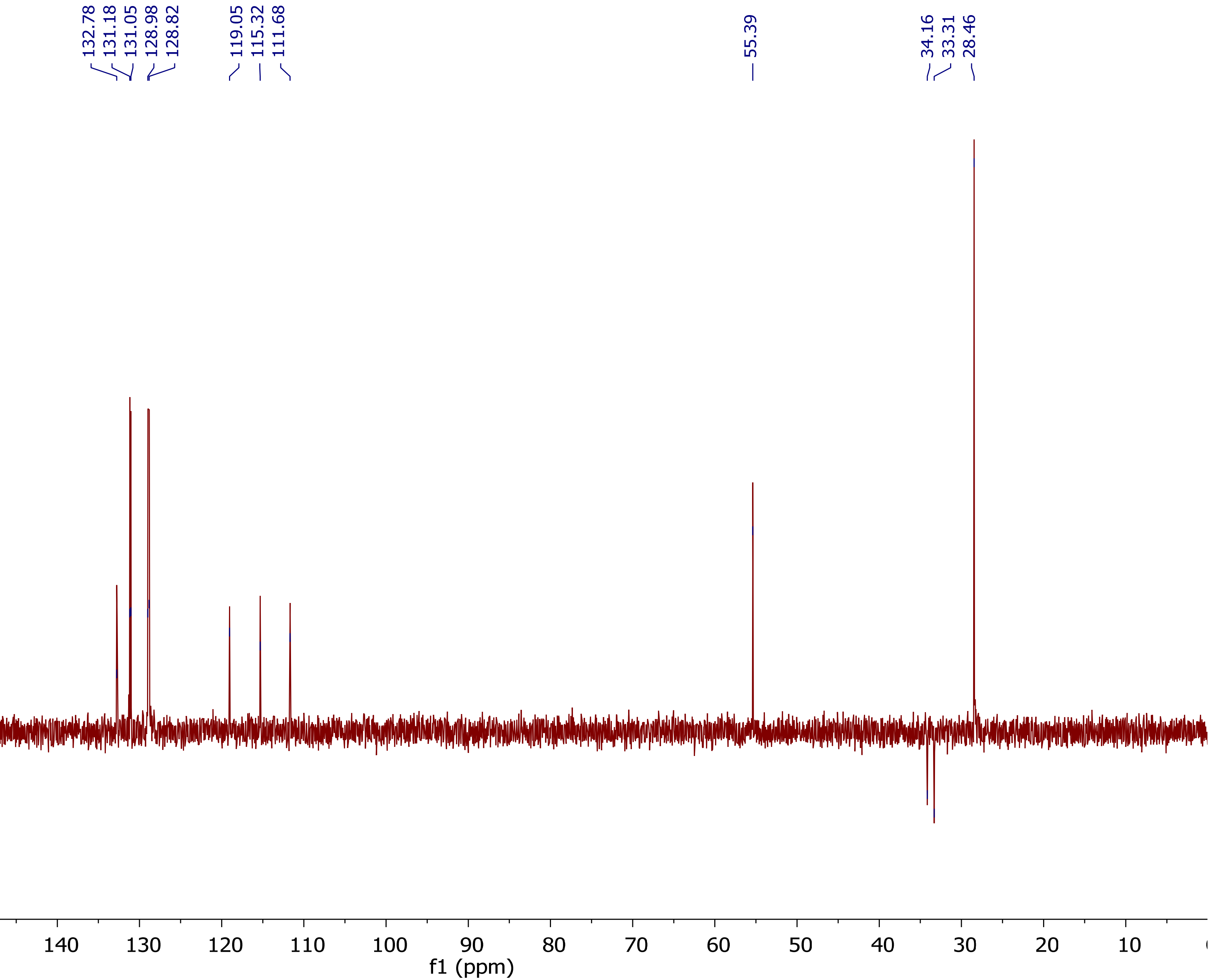

KA628.101.{31P}INVGATED.31.fid
/ILDT KA628.101

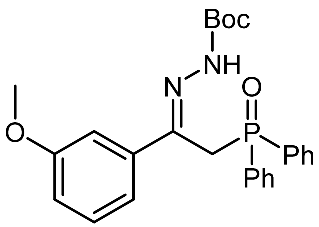

Z/*E* = 12 : 1

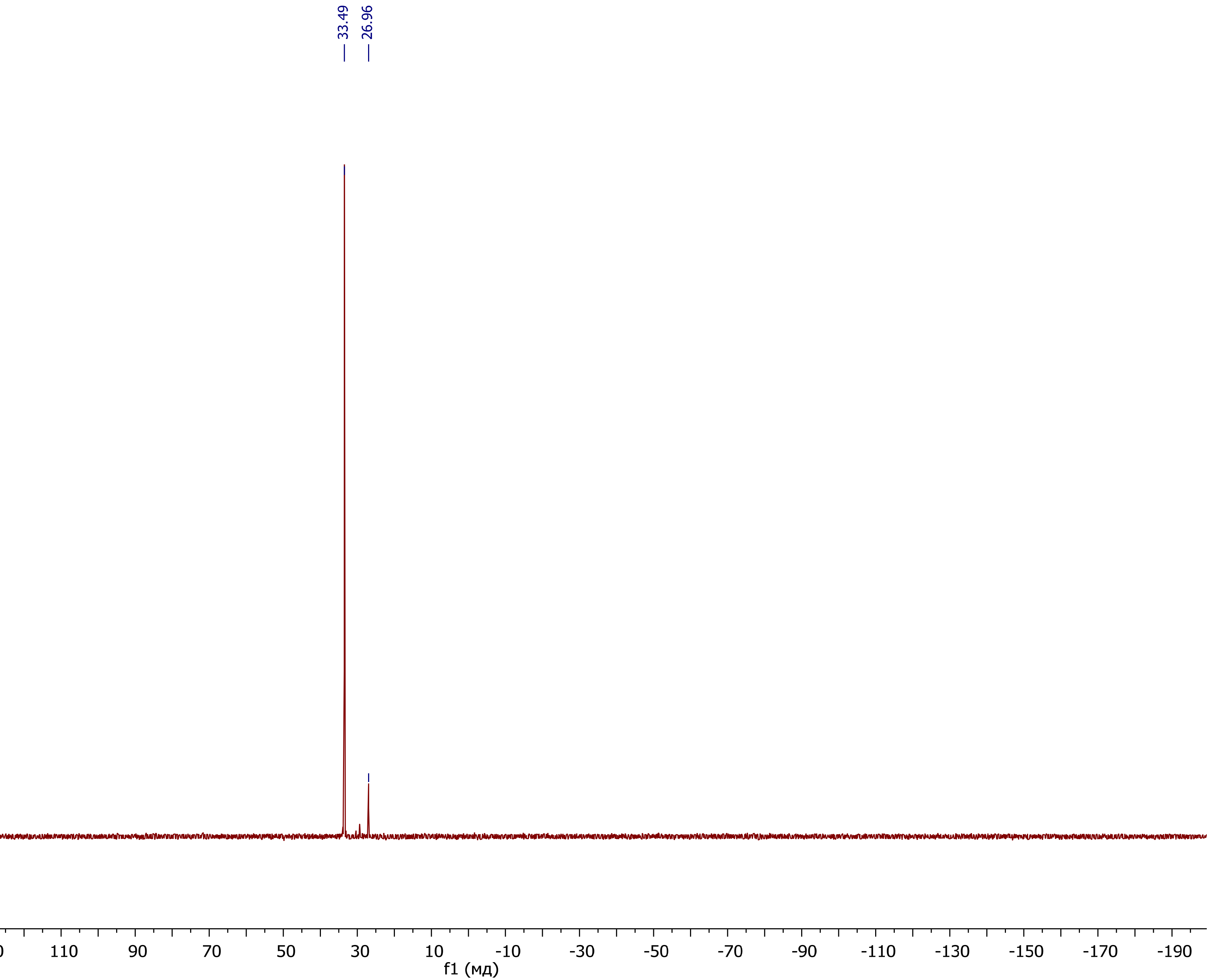

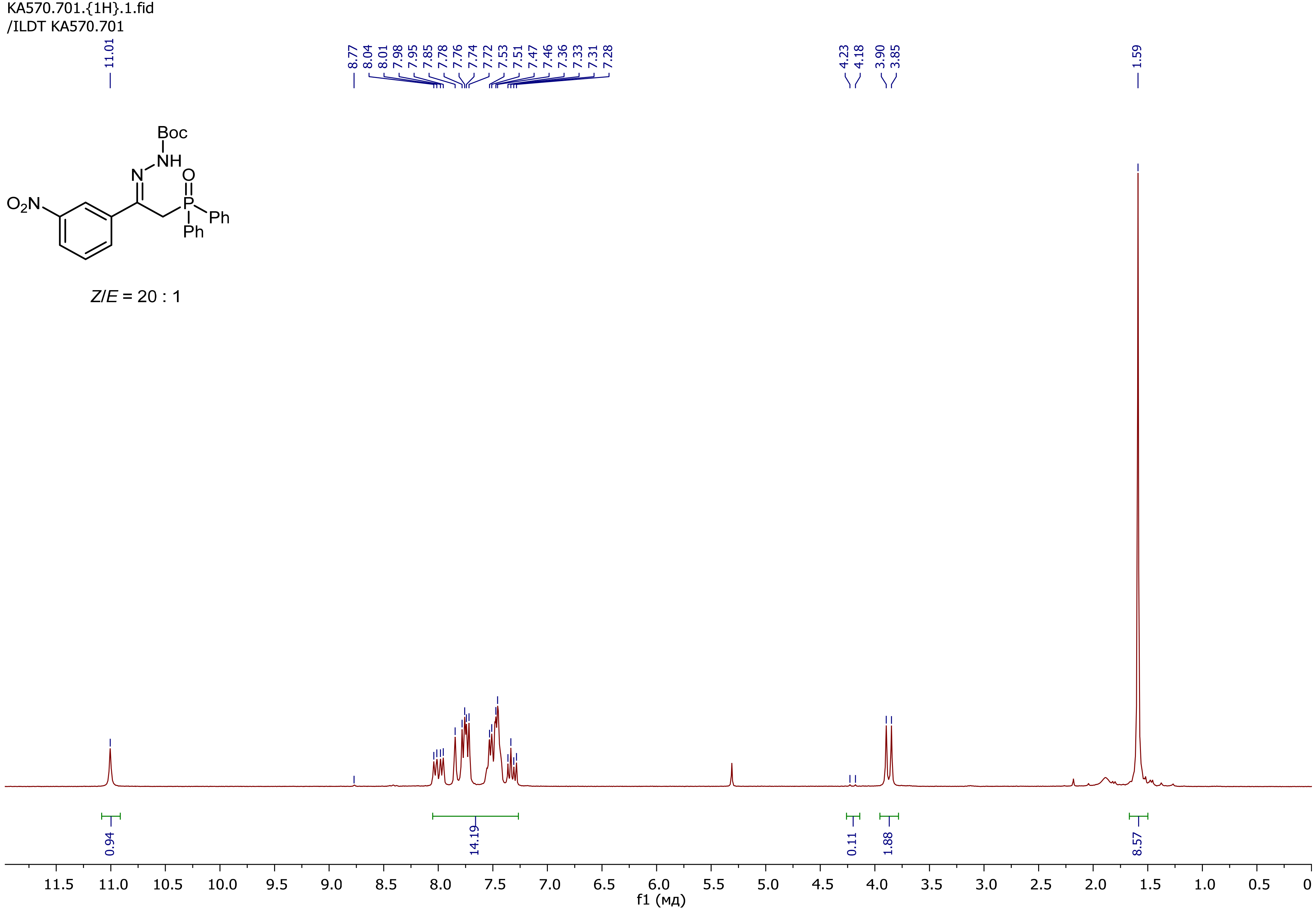
KA570.701.{1H}.1.fid
/ILDT KA570.701
11.01
8.77 8.04 8.01 7.98 7.95 7.85 7.78 7.76 7.74 7.72 7.53 7.51 7.47 7.46 7.36 7.33 7.31 7.28
4.23 4.18 3.90 3.85
1.59
Boc
NH
N
O
O_2N
P
Ph
Ph
Z/E = 20 : 1
0.94
14.19
0.11
1.88
8.57
11.5 11.0 10.5 10.0 9.5 9.0 8.5 8.0 7.5 7.0 6.5 6.0 5.5 5.0 4.5 4.0 3.5 3.0 2.5 2.0 1.5 1.0 0.5 0
f1 (мд)

KA570.701.{13C}.2.fid
/ILDT KA570.701

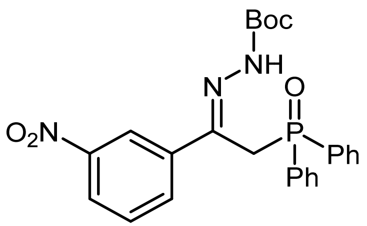

Z/*E* = 20 : 1

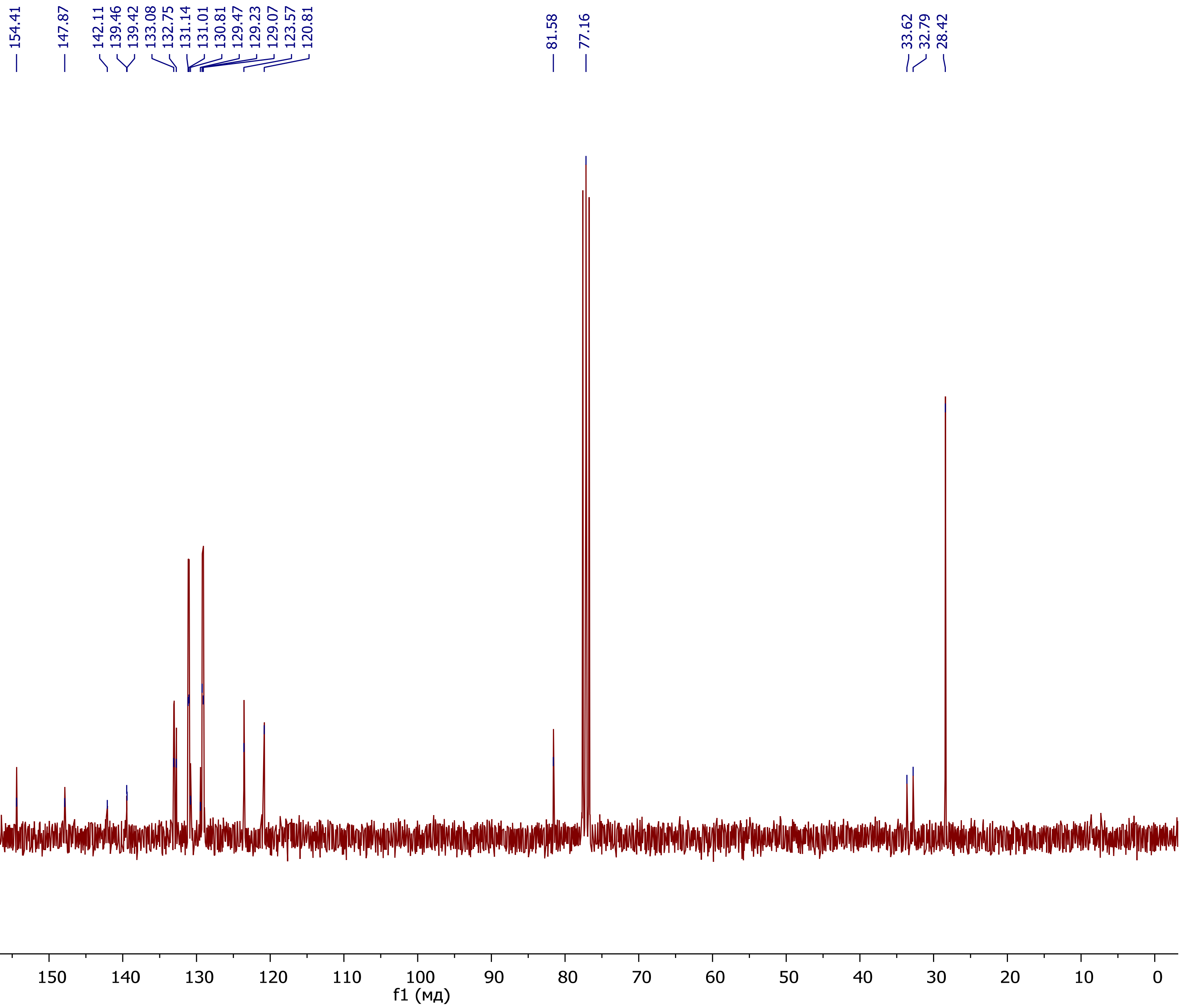

KA570.701.{13C}deptsp135.3.fid
/ILDT KA570.701

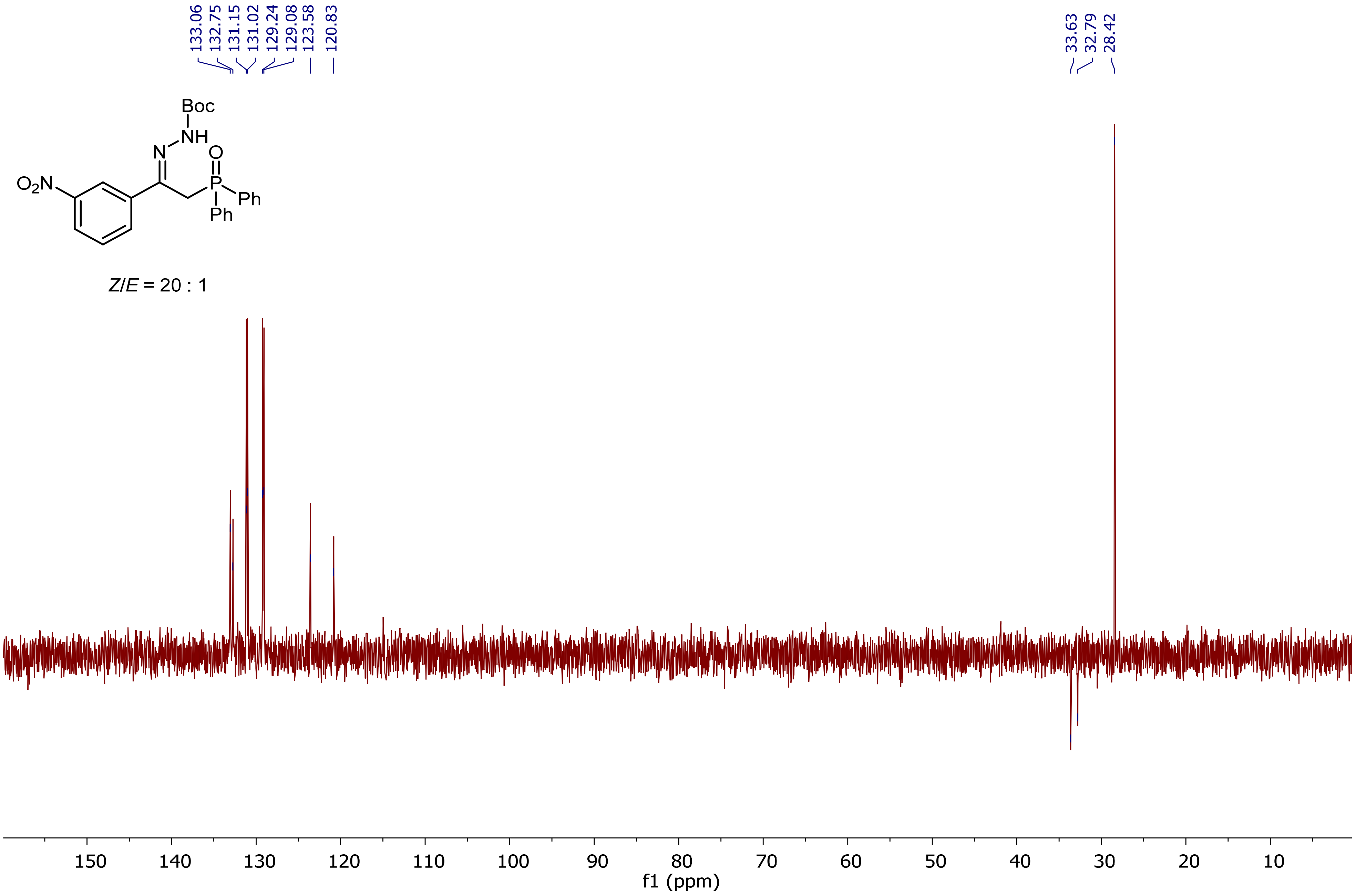

Z/*E* = 20 : 1

KA570.701.{31P}INVGATED.31.fid
/ILDT KA570.701

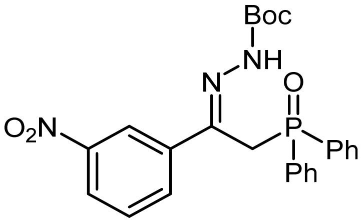

Z/*E* = 20 : 1

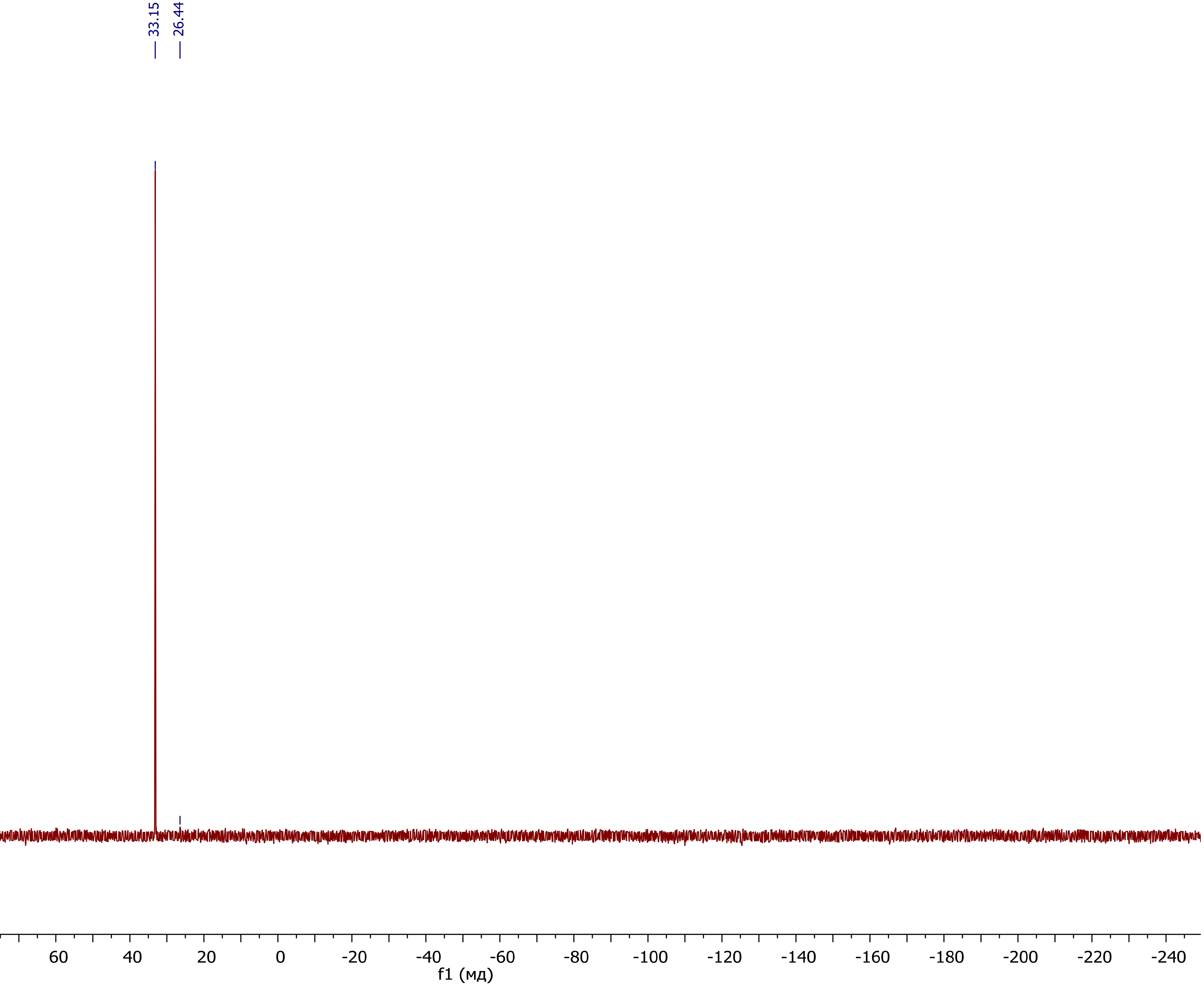

FT-IR

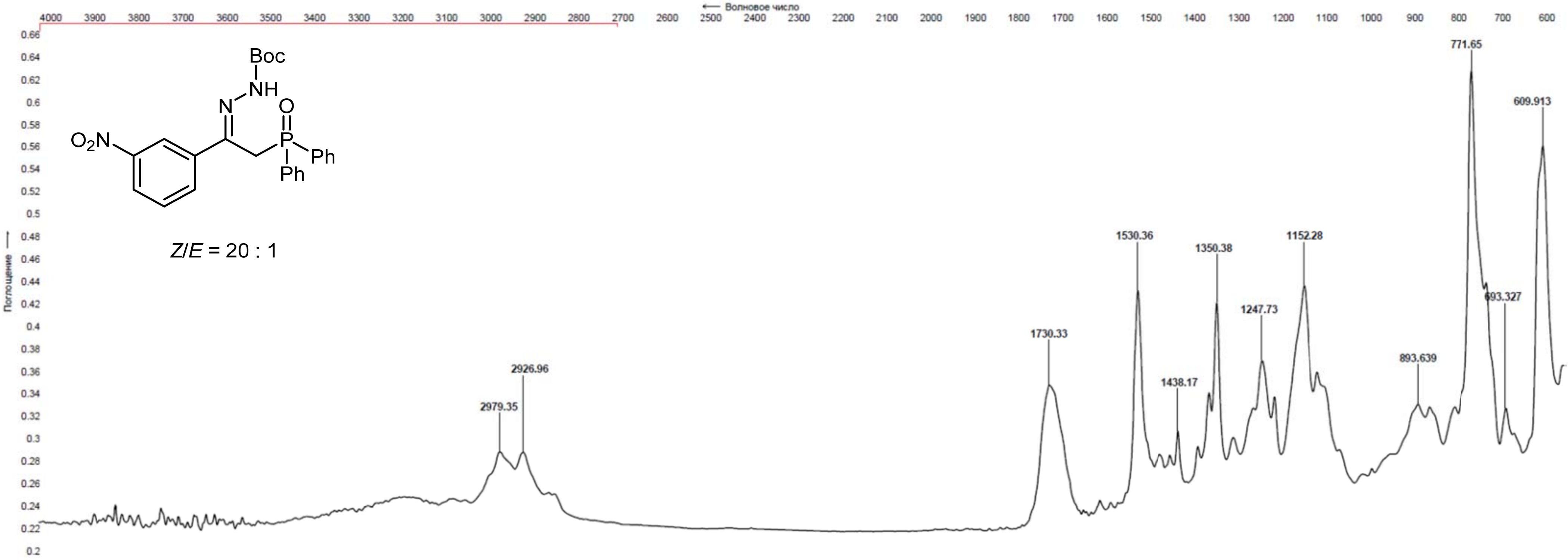
Волновое число
Поглощение
Boc
NH
N
O
P
Ph
Ph
O_2N
Z/E = 20 : 1
2979.35
2926.96
1730.33
1530.36
1438.17
1350.38
1247.73
1152.28
893.639
771.65
693.327
609.913

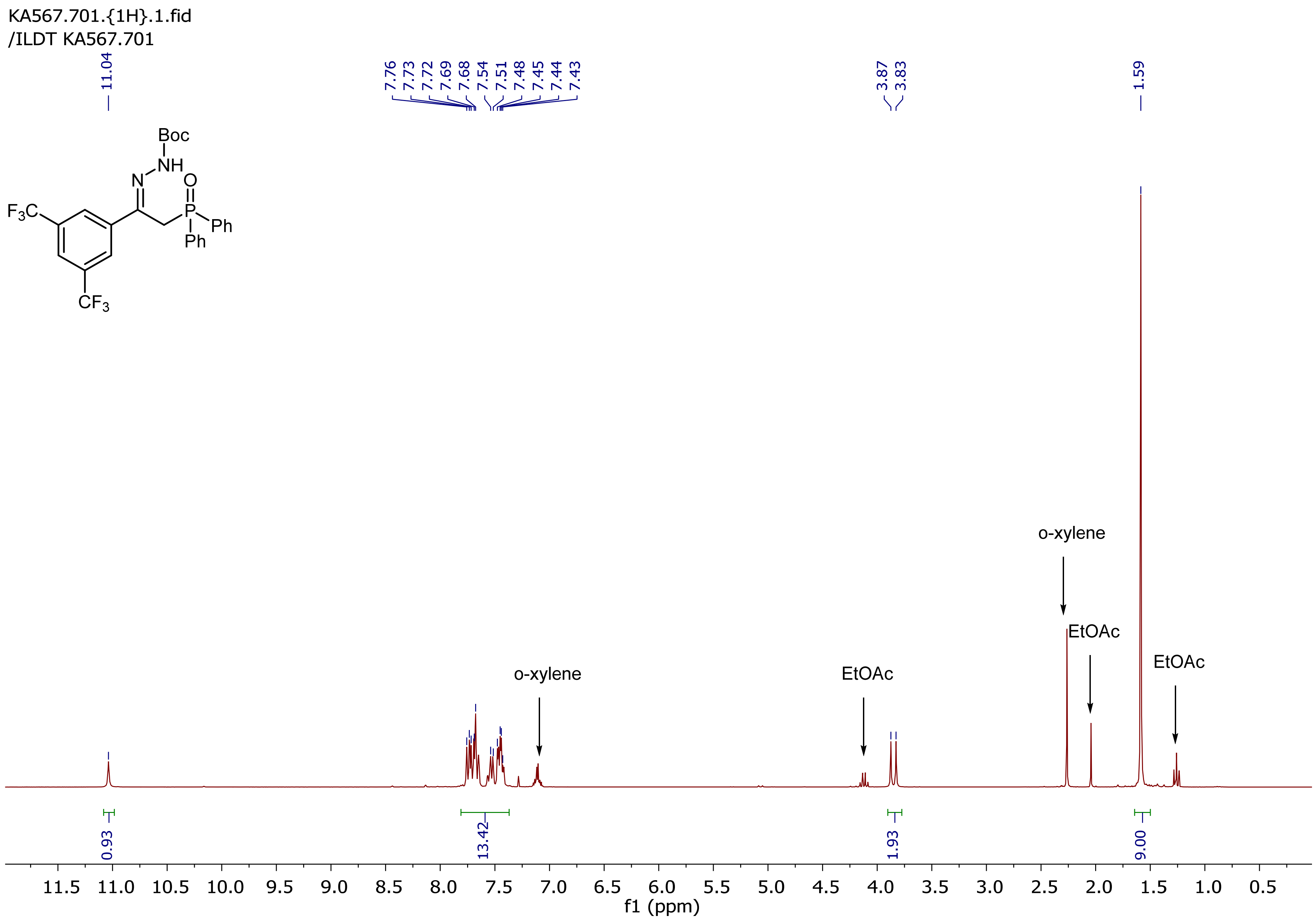

KA567.701.{1H}.1.fid
/ILDT KA567.701
11.04
7.76
7.73
7.72
7.69
7.68
7.54
7.51
7.48
7.45
7.44
7.43
3.87
3.83
1.59
Boc
NH
N
O
P
Ph
Ph
F3C
CF3
o-xylene
EtOAc
o-xylene
EtOAc
EtOAc
0.93
13.42
1.93
9.00
11.5
11.0
10.5
10.0
9.5
9.0
8.5
8.0
7.5
7.0
6.5
6.0
5.5
5.0
4.5
4.0
3.5
3.0
2.5
2.0
1.5
1.0
0.5
f1 (ppm)

KA567.701.{13C}.2.fid
/ILDT KA567.701

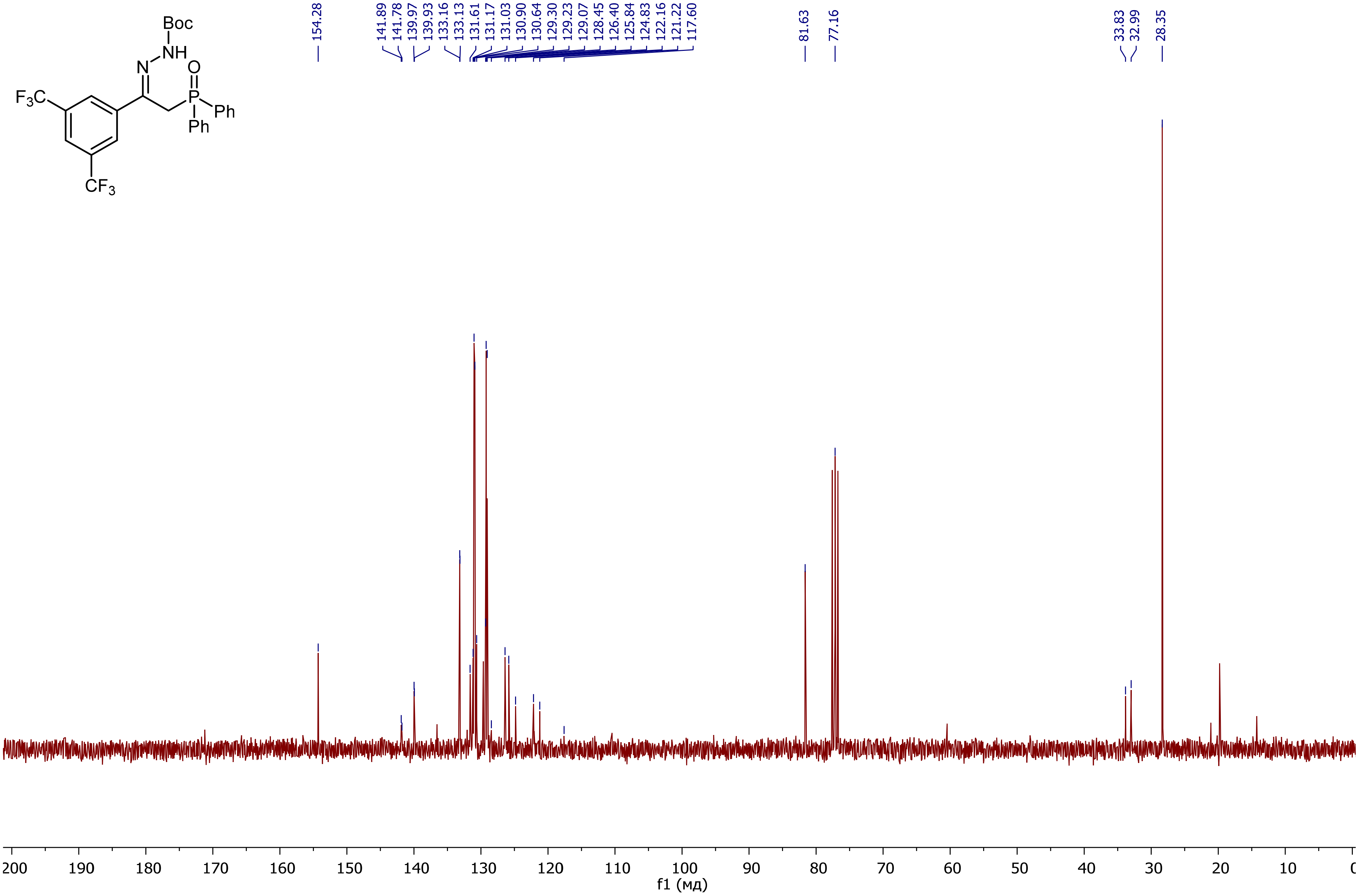

KA567.701.{19F}.19.fid
/ILDT KA567.701

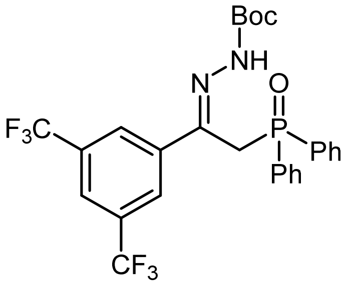

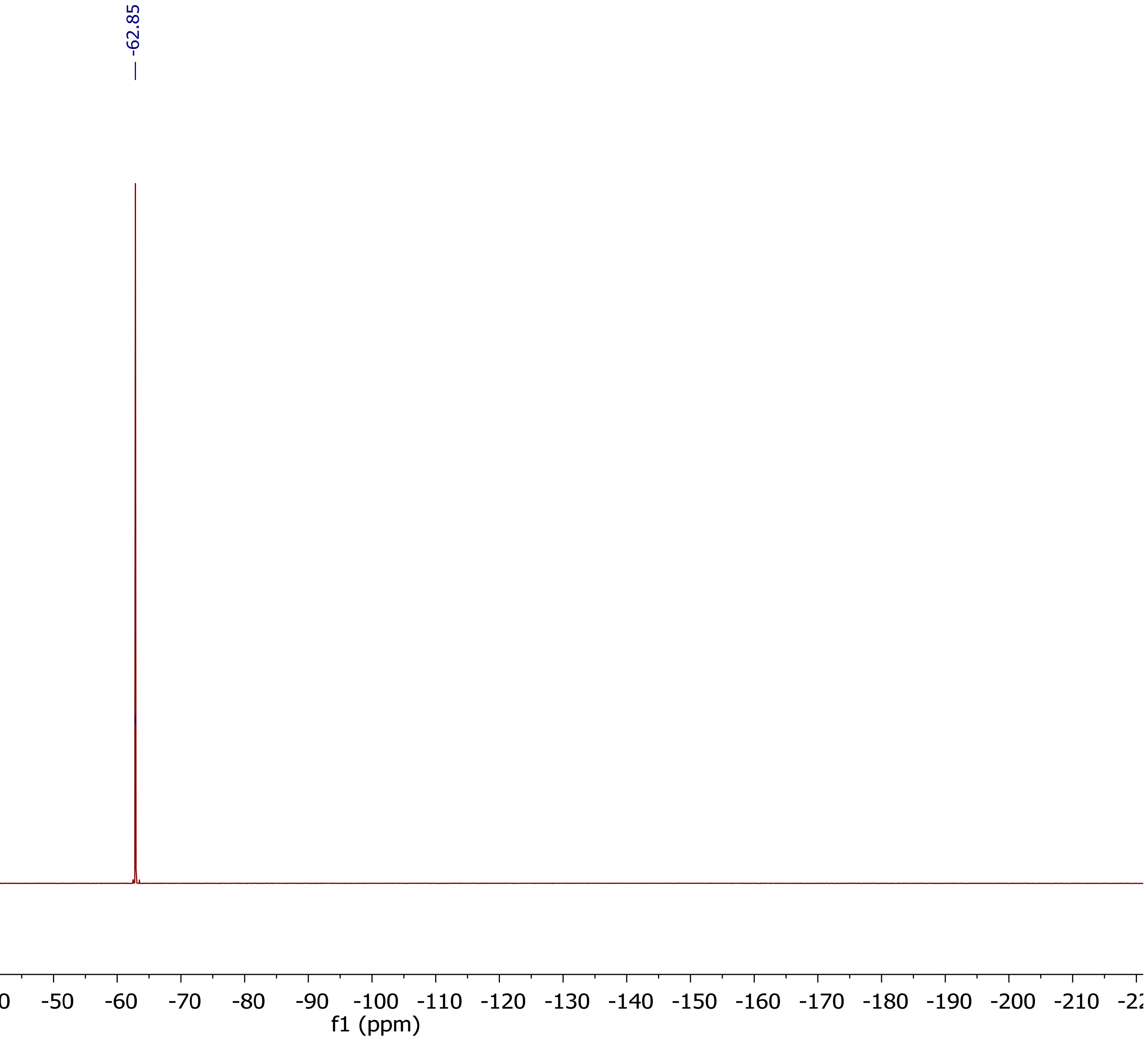

KA567.701.{31P}INVGATED.31.fid
/ILDT KA567.701

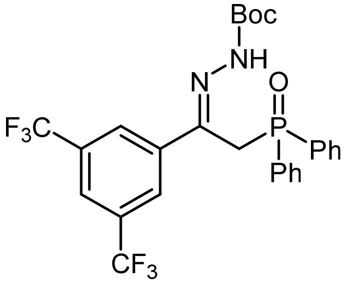

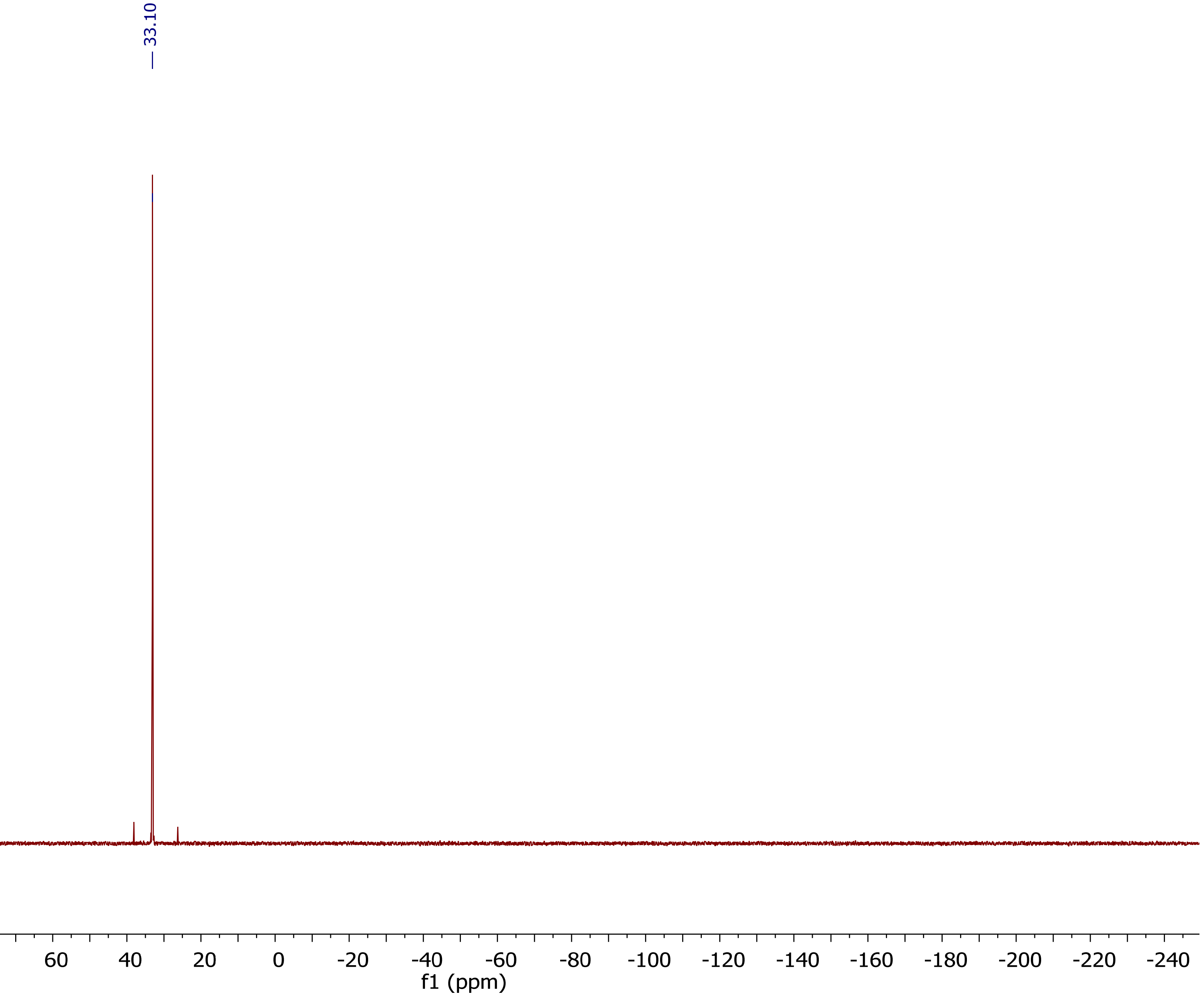

KA648.301-1.{1H}.1.fid
/ILDT KA648.301-1

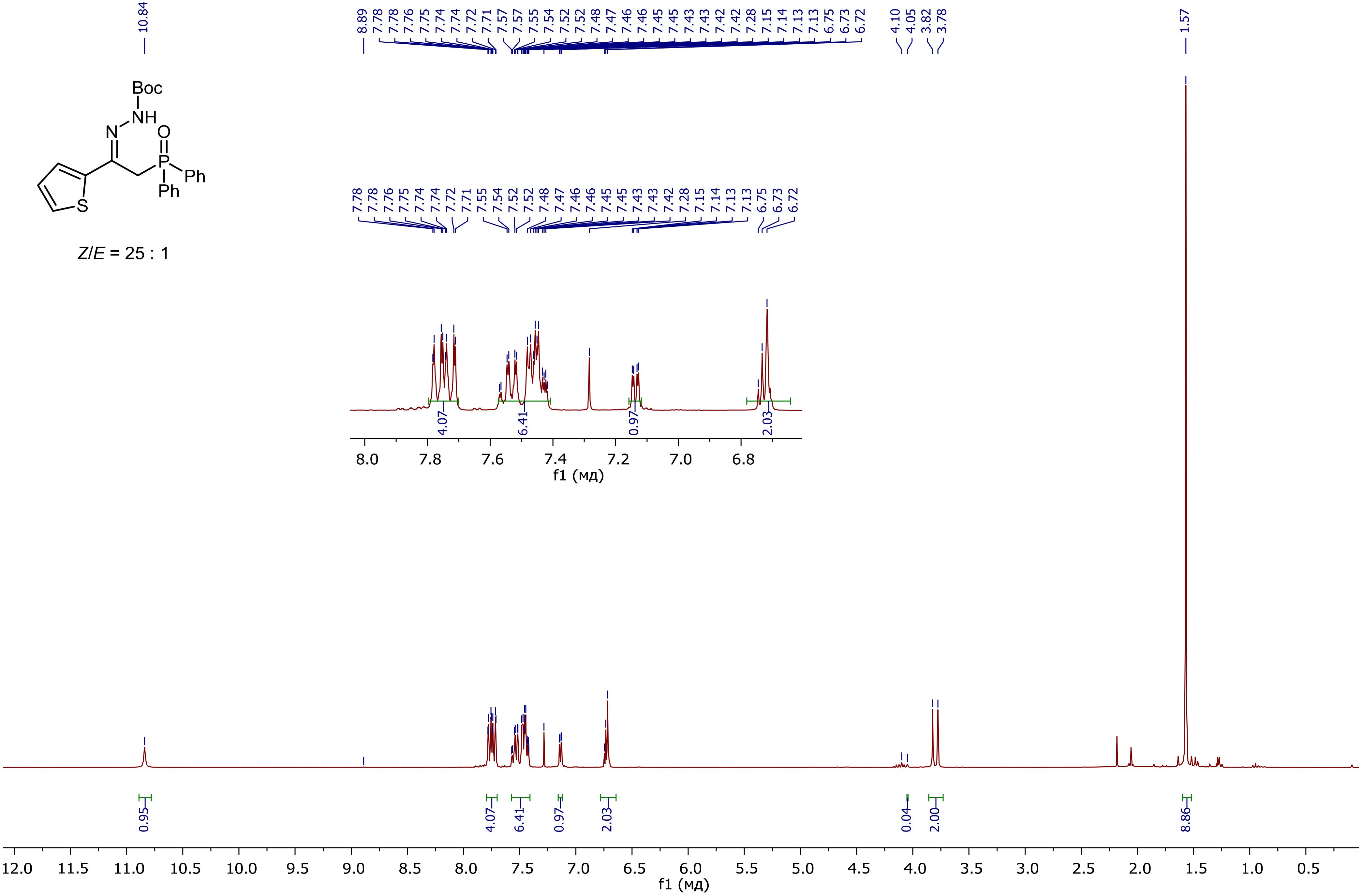

Z/*E* = 25 : 1

KA648.301-1.{13C}.2.fid
/ILDT KA648.301-1

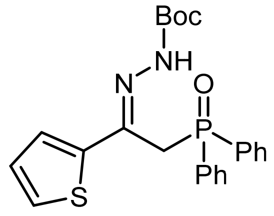

Z/*E* = 25 : 1

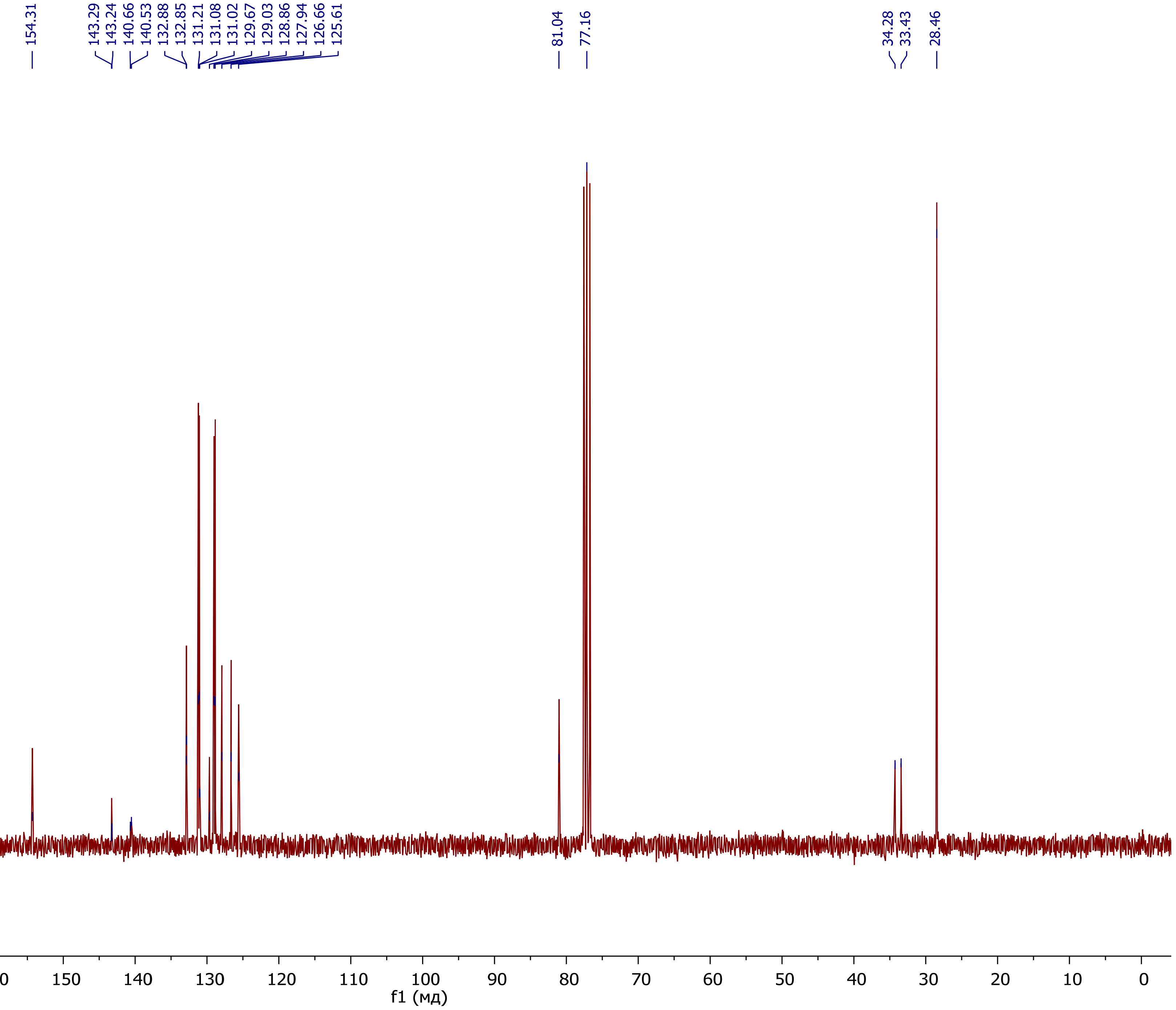

KA648.301-1.{13C}deptsp135.3.fid
/ILDT KA648.301-1

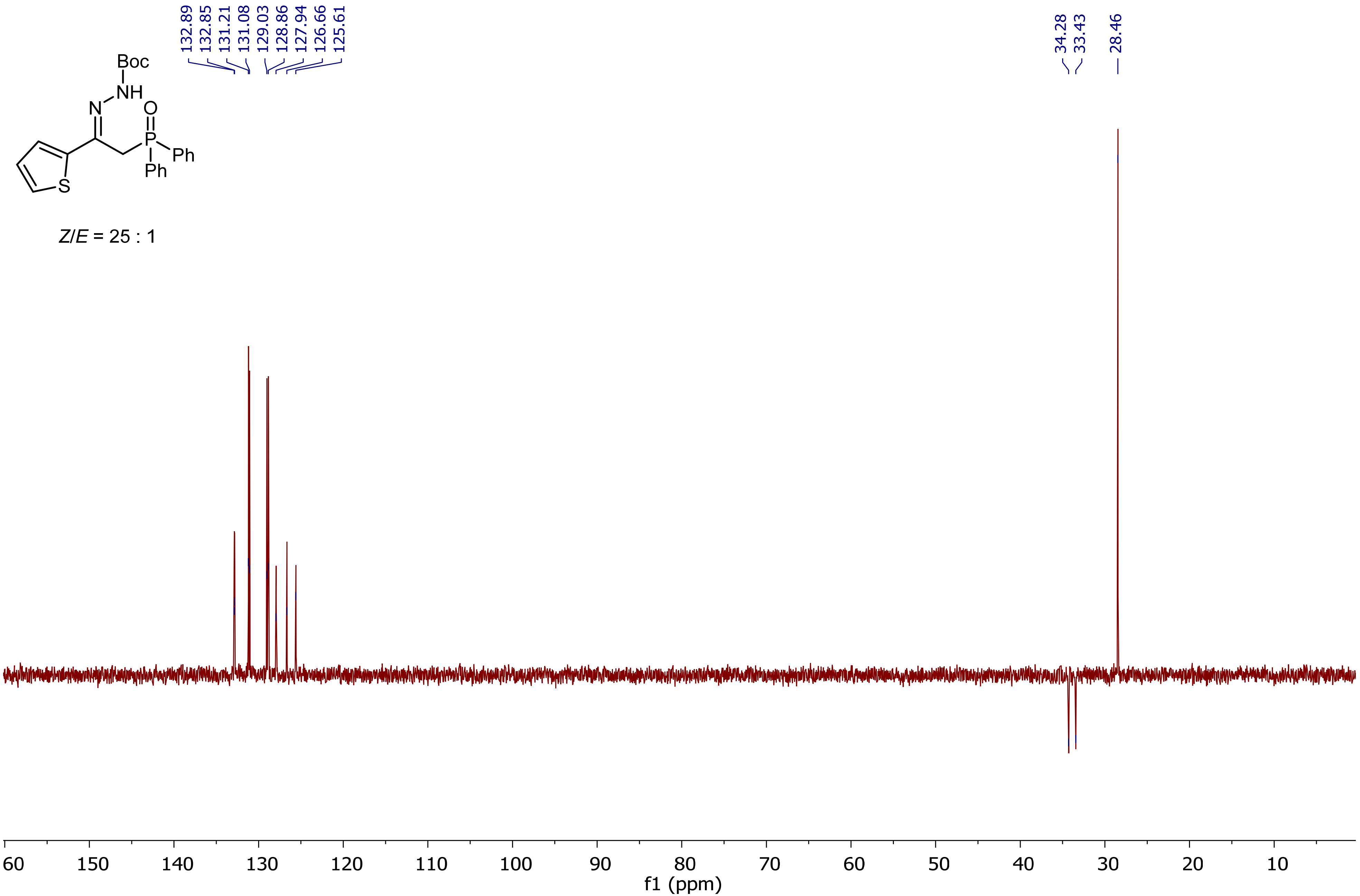

KA648.301-1.{31P}INVGATED.31.fid
/ILDT KA648.301-1

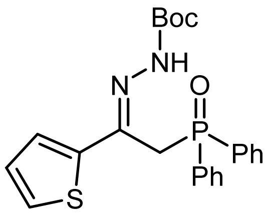

Z/*E* = 25 : 1

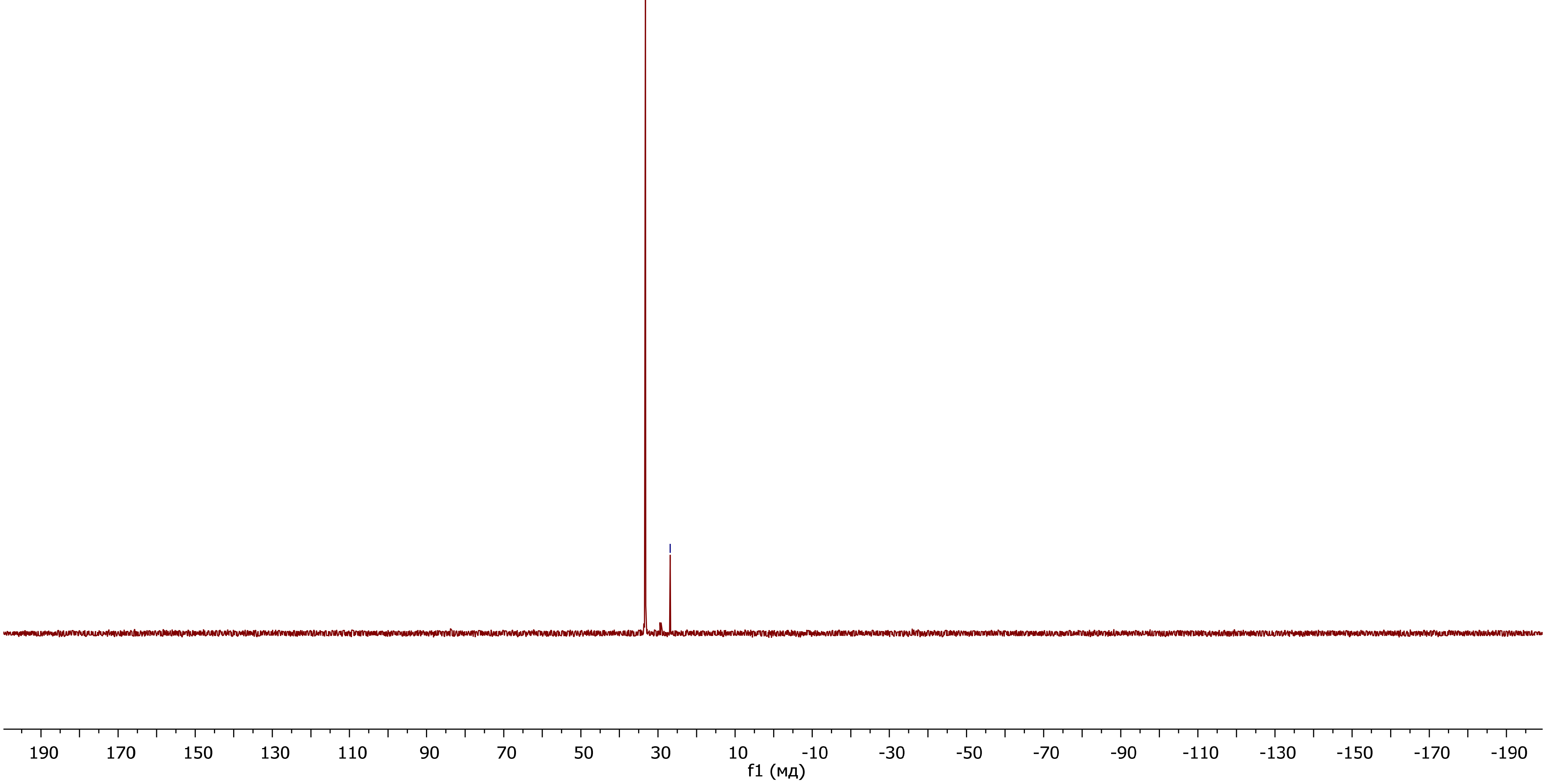

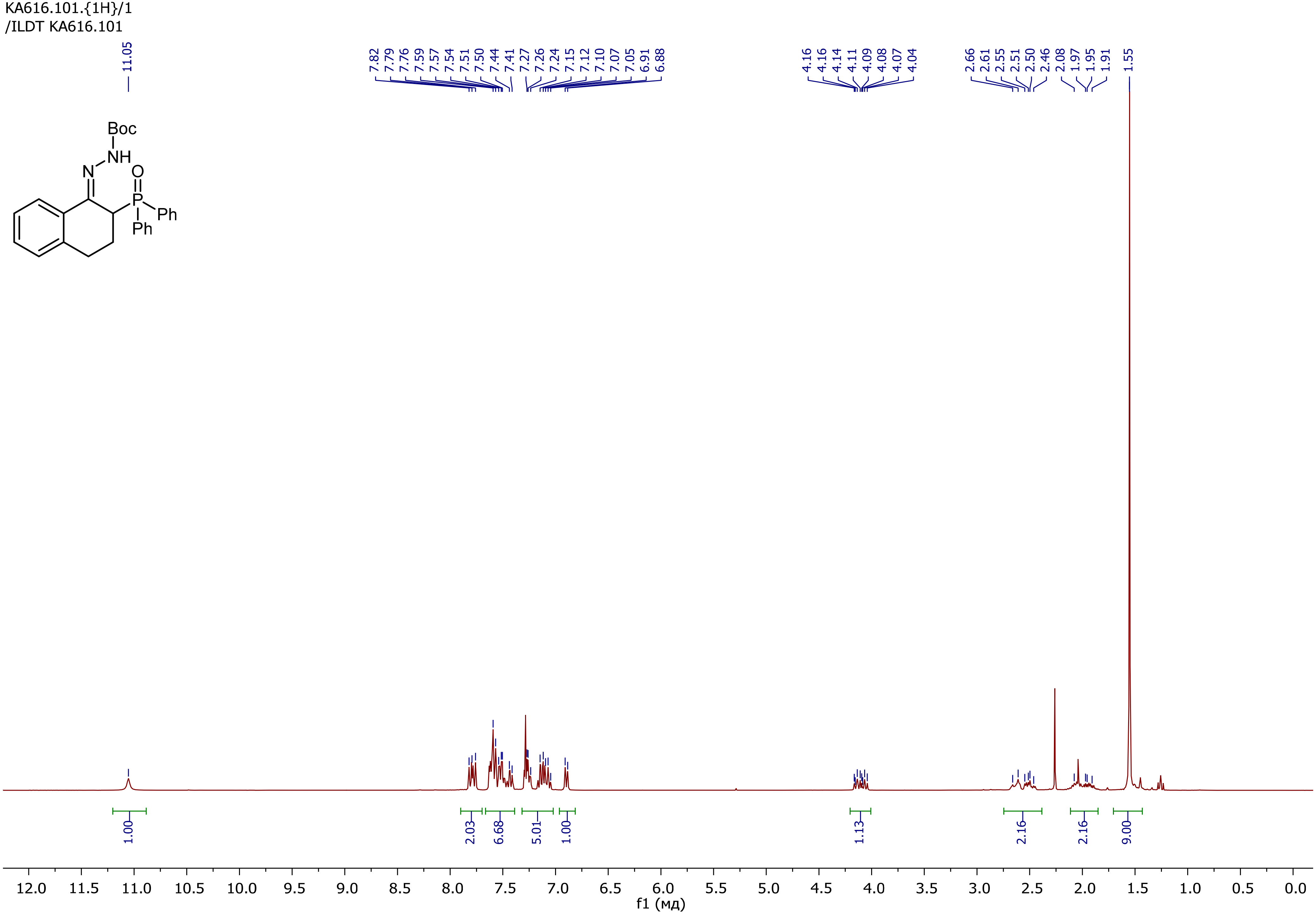
KA616.101.{1H}/1
/ILDT KA616.101
11.05
7.82 7.79 7.76 7.59 7.57 7.54 7.51 7.50 7.44 7.41 7.27 7.26 7.24 7.15 7.12 7.10 7.07 7.05 6.91 6.88
4.16 4.16 4.14 4.11 4.09 4.08 4.07 4.04
2.66 2.61 2.55 2.51 2.50 2.46 2.08 1.97 1.95 1.91
1.55
Boc
NH
N
O
P
Ph
Ph
1.00
2.03
6.68
5.01
1.00
1.13
2.16
2.16
9.00
12.0 11.5 11.0 10.5 10.0 9.5 9.0 8.5 8.0 7.5 7.0 6.5 6.0 5.5 5.0 4.5 4.0 3.5 3.0 2.5 2.0 1.5 1.0 0.5 0.0
f1 (мд)

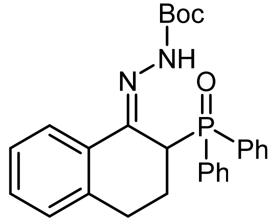
Boc
NH
N
O
P
Ph
Ph

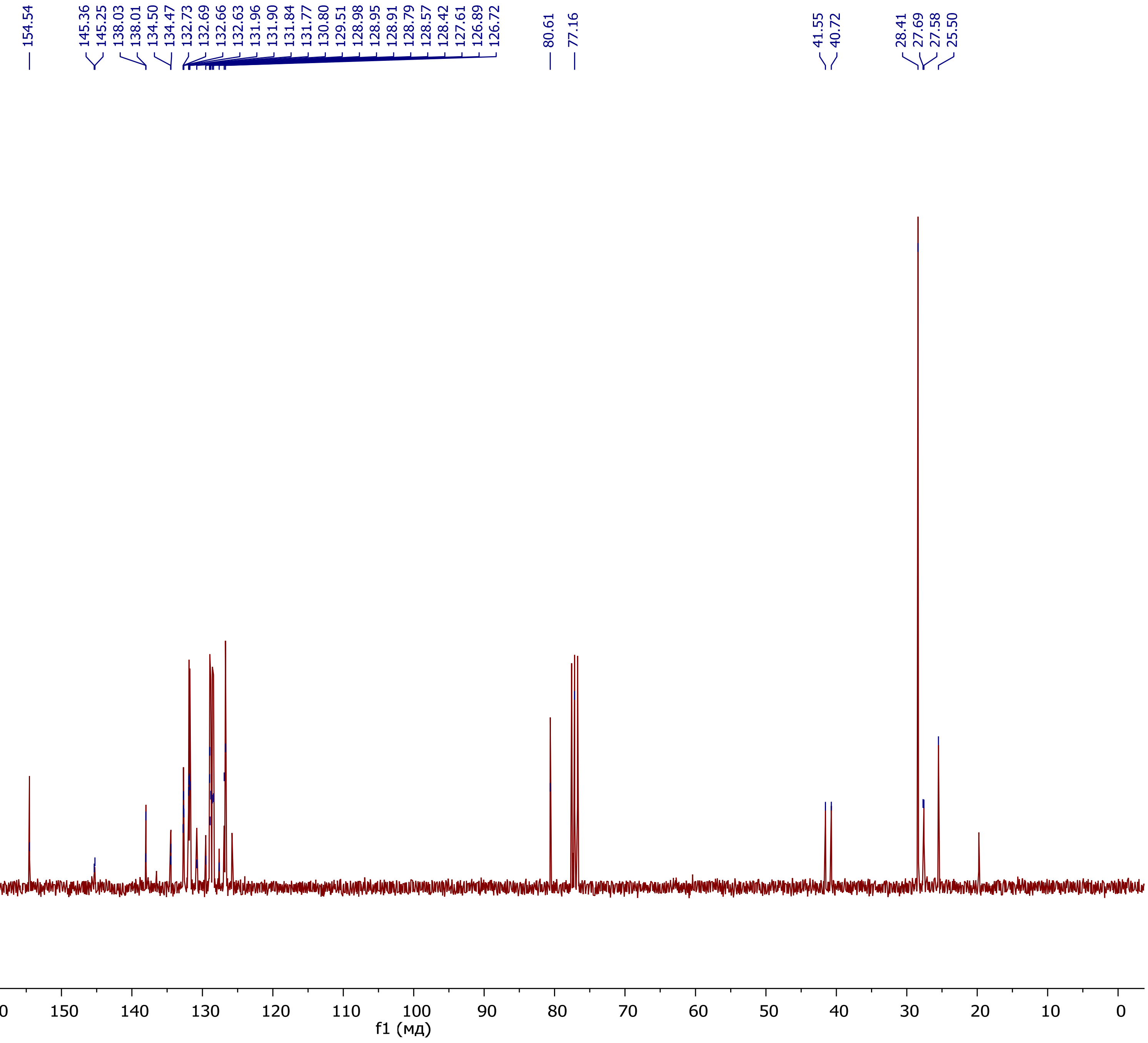
KA616.101.{13C}.2.fid
/ILDT KA616.101
154.54
145.36
145.25
138.03
138.01
134.50
134.47
132.73
132.69
132.66
132.63
131.96
131.90
131.84
131.77
130.80
129.51
128.98
128.95
128.91
128.79
128.57
128.42
127.61
126.89
126.72
80.61
77.16
41.55
40.72
28.41
27.69
27.58
25.50
200
190
180
170
160
150
140
130
120
110
100
90
80
70
60
50
40
30
20
10
0
f1 (мд)

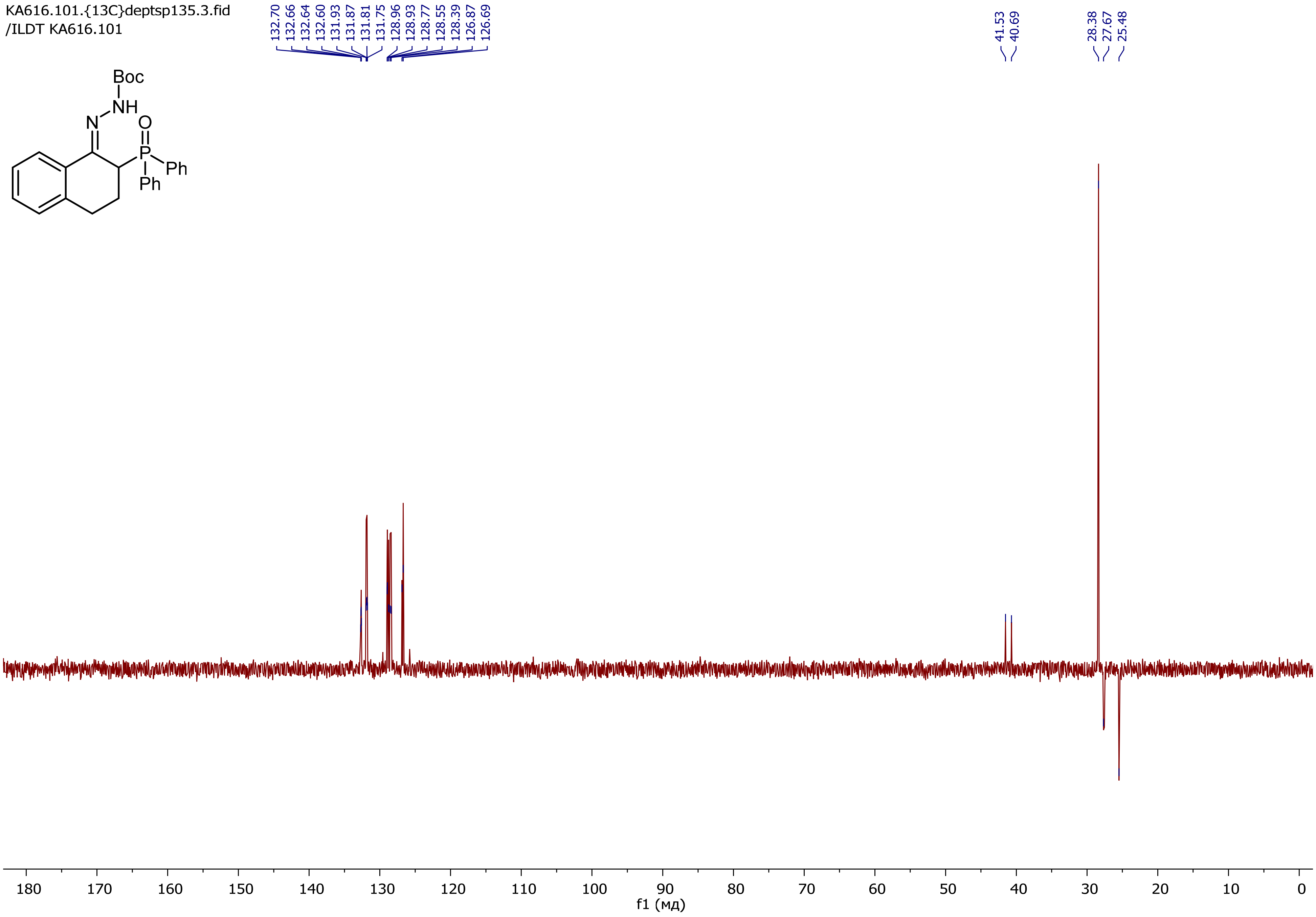
KA616.101.{13C}deptsp135.3.fid
/ILDT KA616.101
Boc
NH
N
O
P
Ph
Ph
132.70
132.66
132.64
132.60
131.93
131.87
131.81
131.75
128.96
128.93
128.77
128.55
128.39
126.87
126.69
41.53
40.69
28.38
27.67
25.48
180
170
160
150
140
130
120
110
100
90
80
70
60
50
40
30
20
10
0
f1 (мд)

KA616.101.{31P}INVGATED/31
/ILDT KA616.101

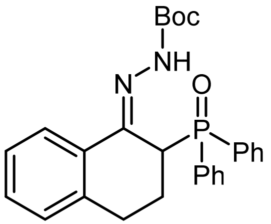

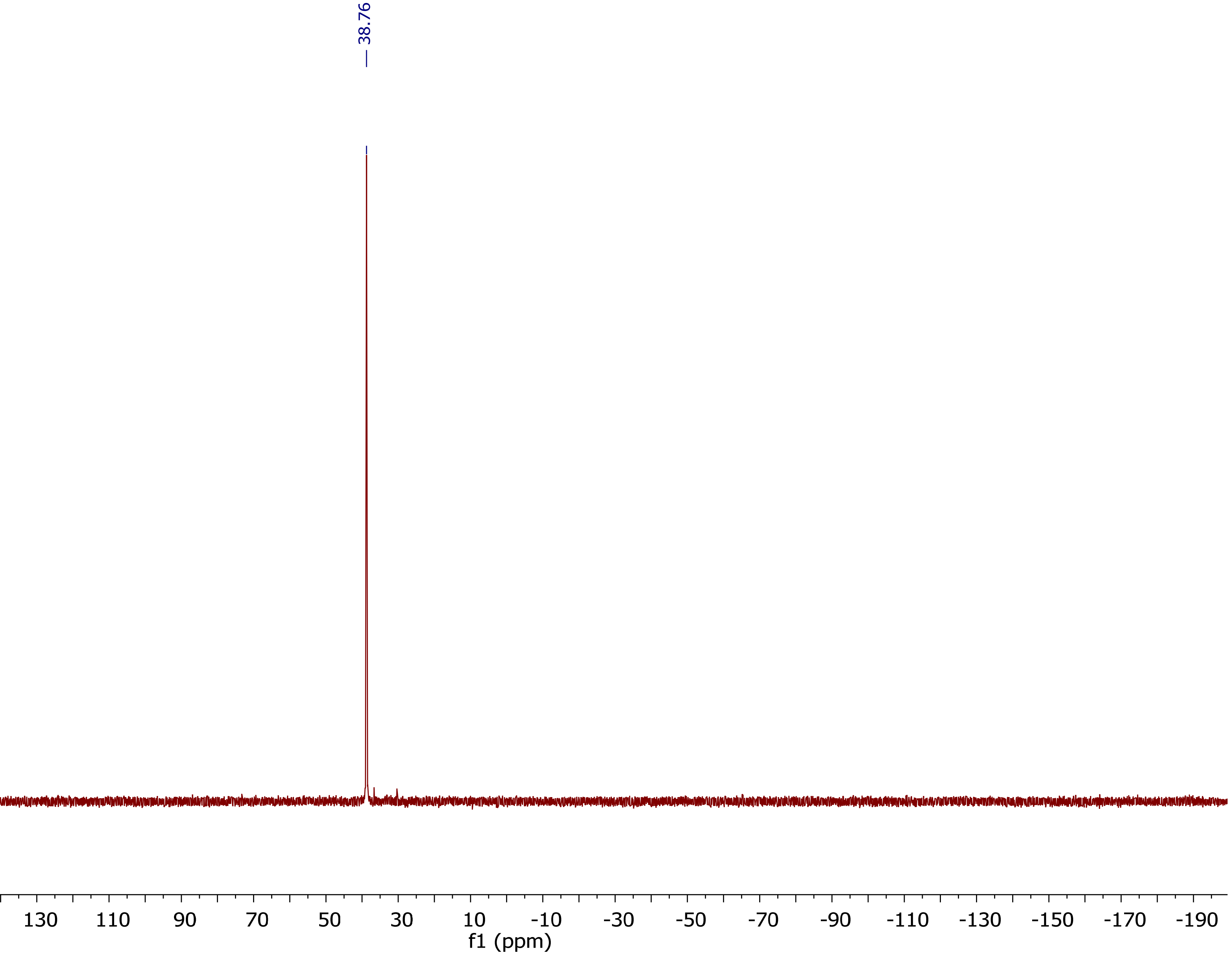

KA572.101.{1H}.1.fid
/ILDT KA572.101

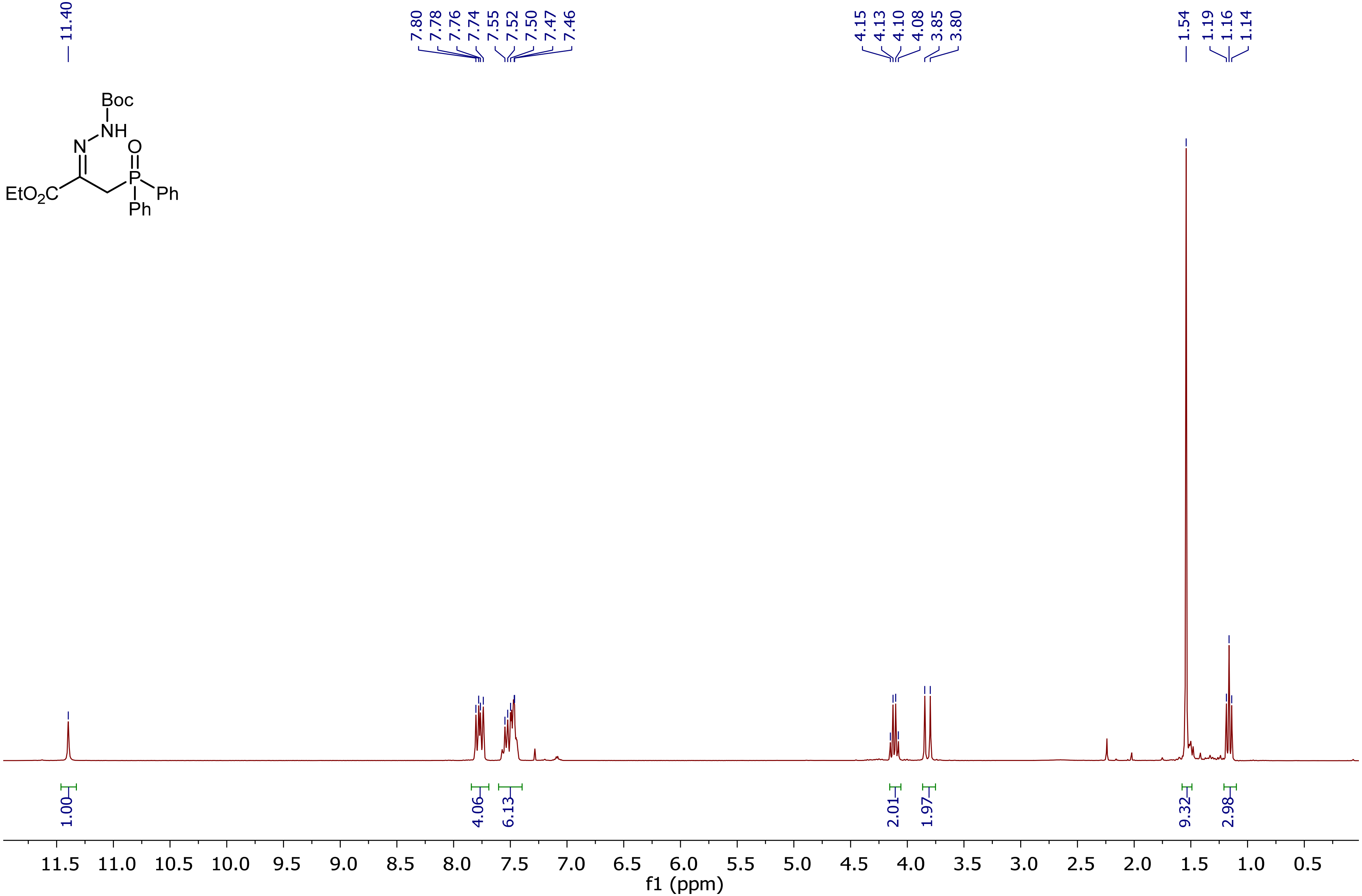

KA572.101.{13C}.2.fid
/ILDT KA572.101

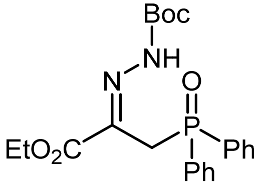

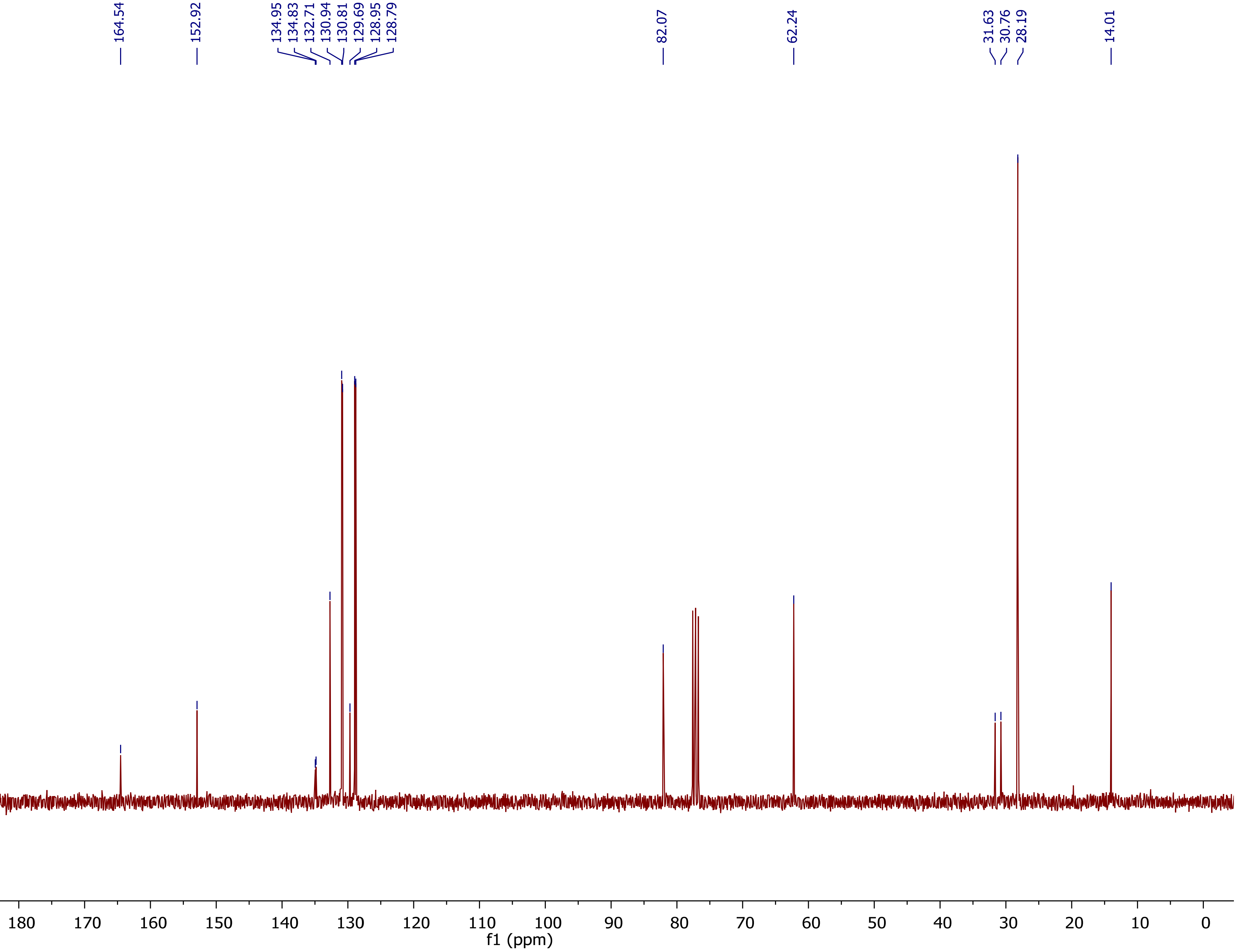

KA572.101.{13C}deptsp135.3.fid
/ILDT KA572.101

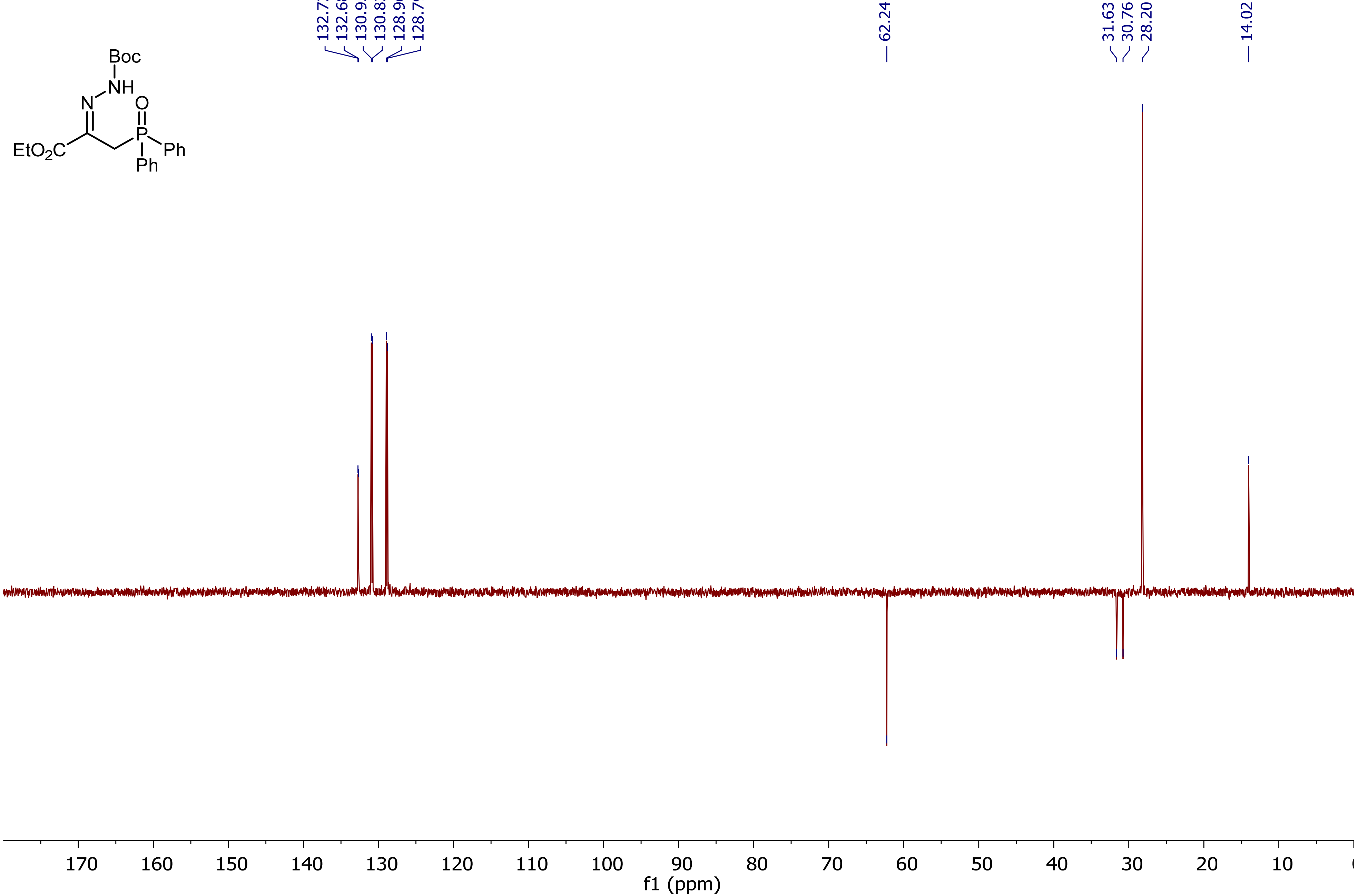

KA572.101.{31P}INVGATED.4.fid
/ILDT KA572.101

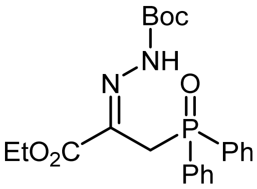

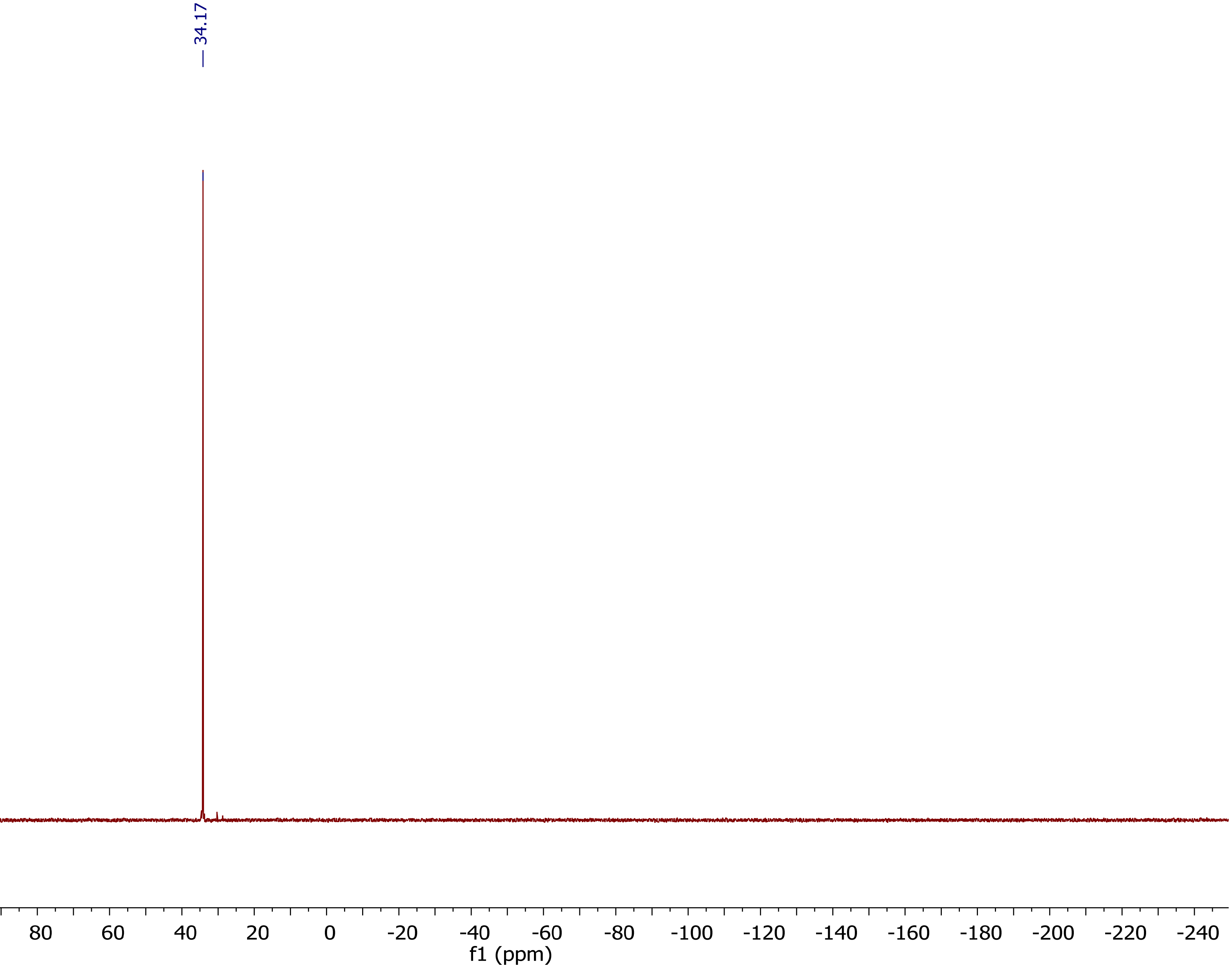

FT-IR

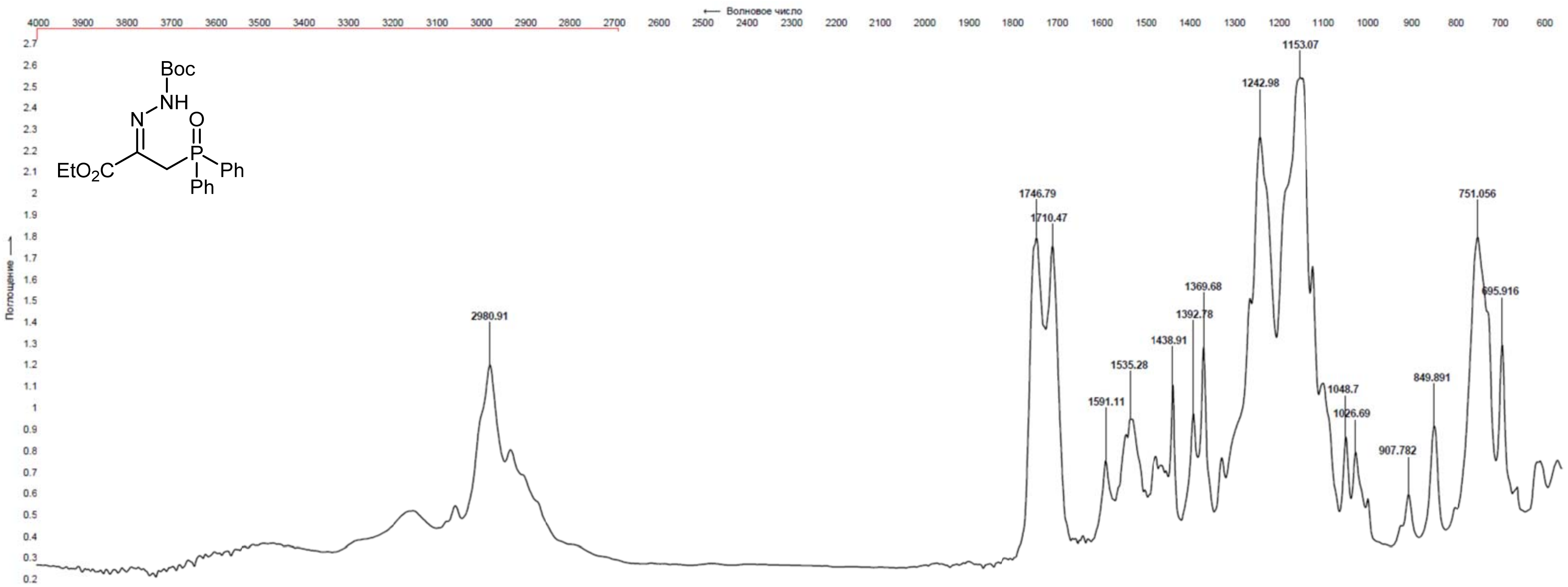
Волновое число
Поглощение
Boc
NH
N
O
P
EtO2C
Ph
Ph
2980.91
1746.79
1710.47
1591.11
1535.28
1438.91
1392.78
1369.68
1242.98
1153.07
1048.7
1026.69
907.782
849.891
751.056
695.916

KA573.101.{1H}.1.fid
/ILDT KA573.101

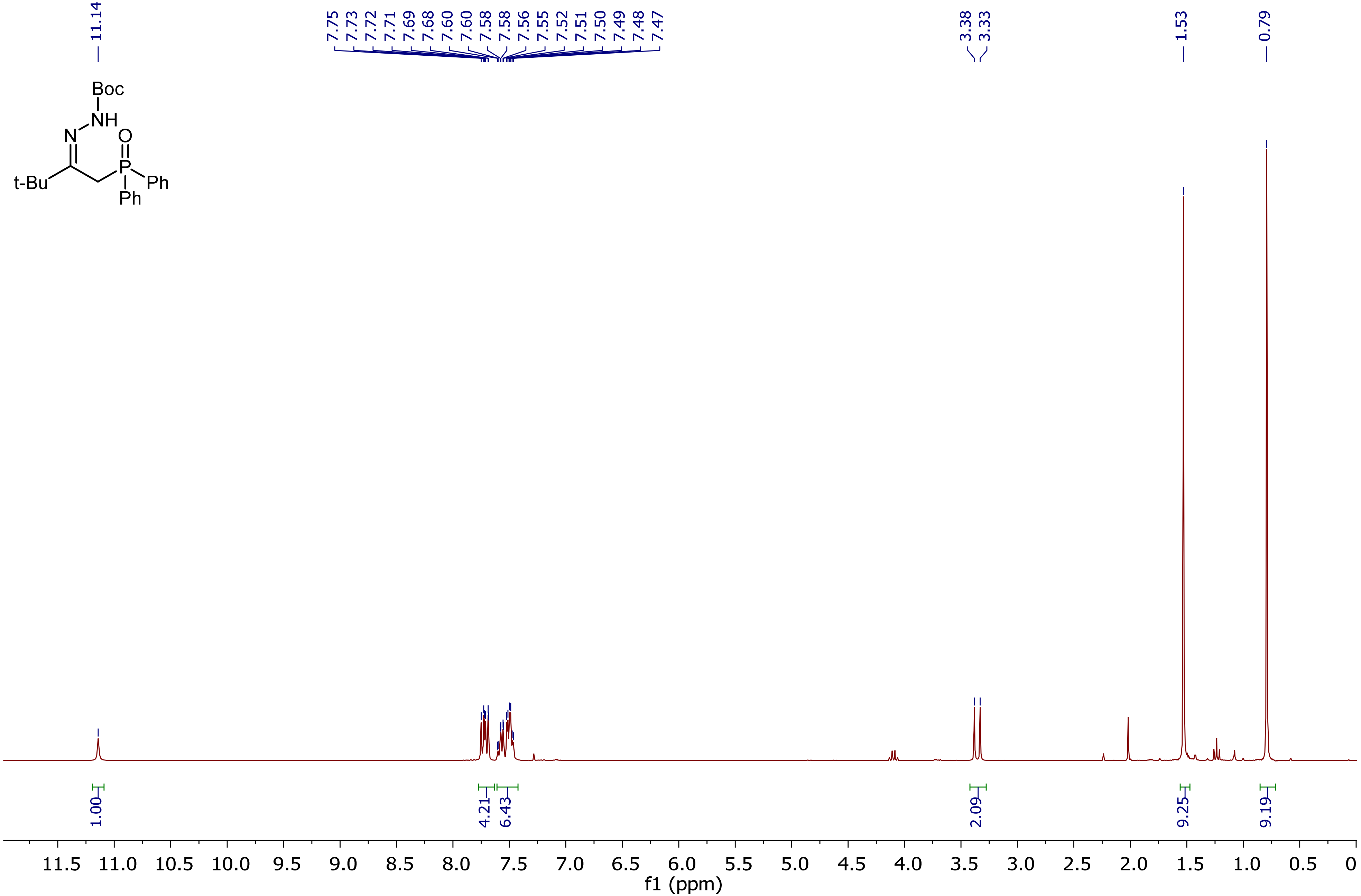

KA573.101.{13C}.2.fid
/ILDT KA573.101

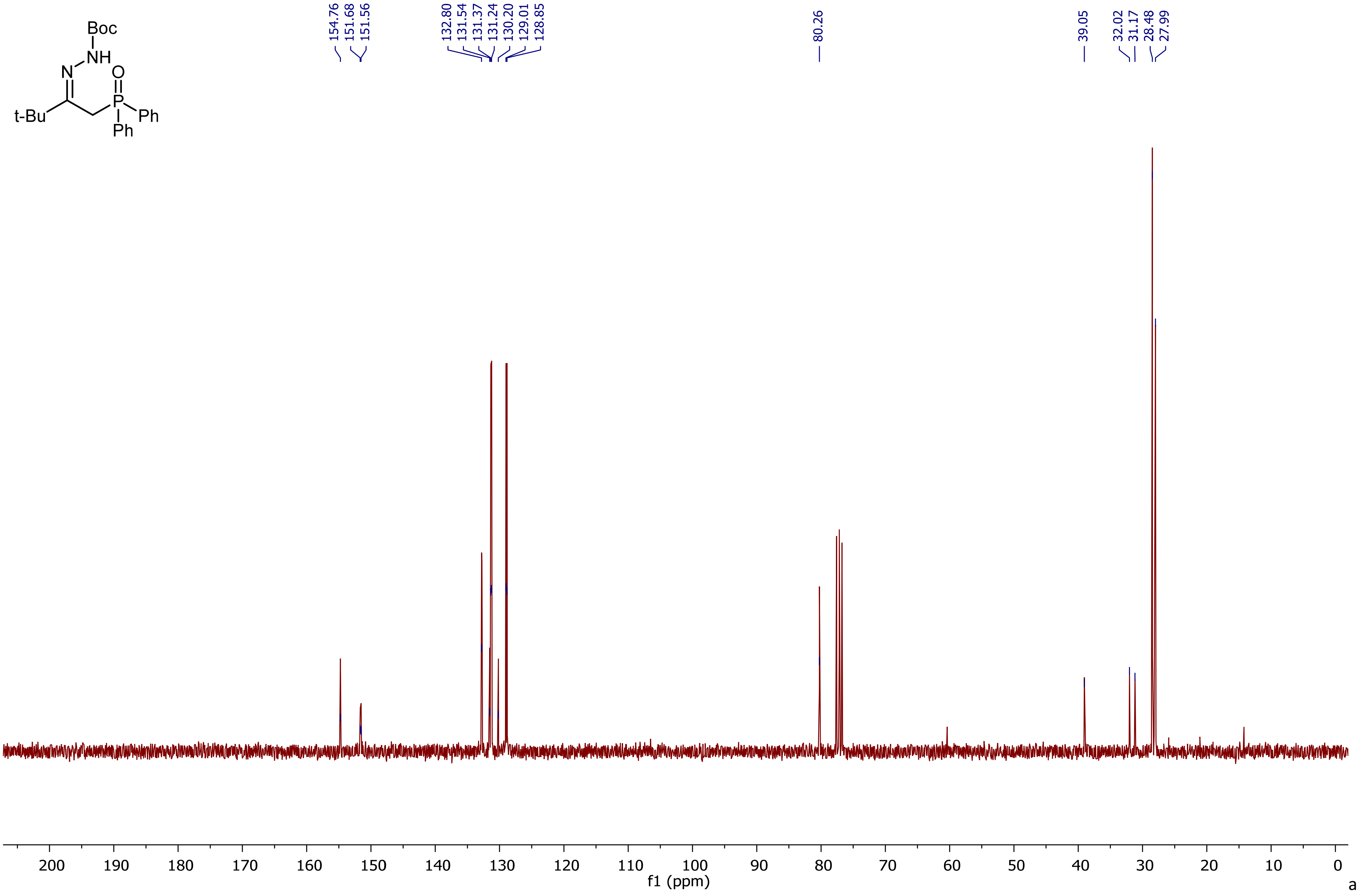

KA573.101.{13C}deptsp135.3.fid
/ILDT KA573.101

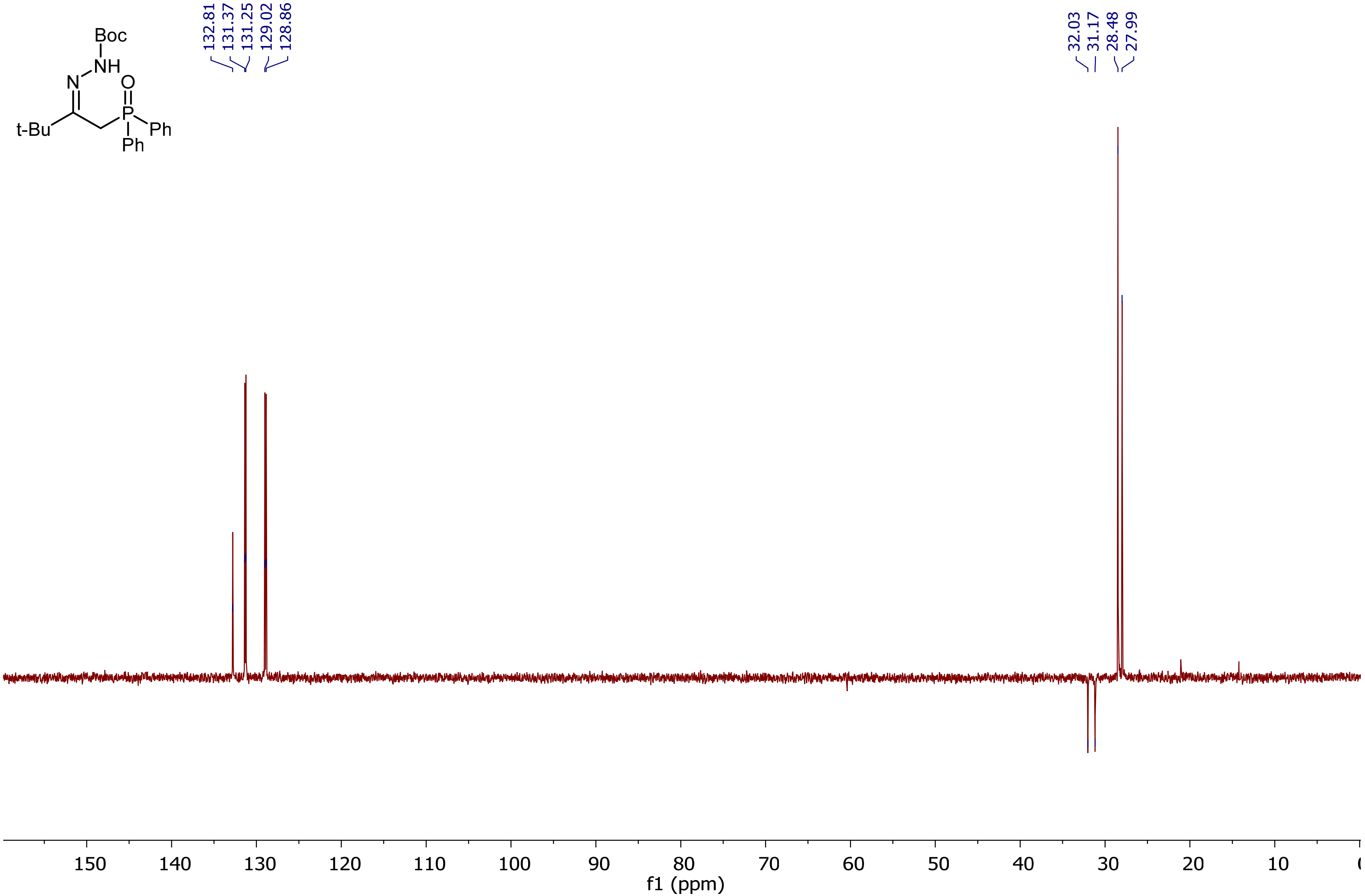

KA573.101.{31P}INVGATED.31.fid
/ILDT KA573.101

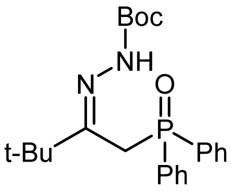

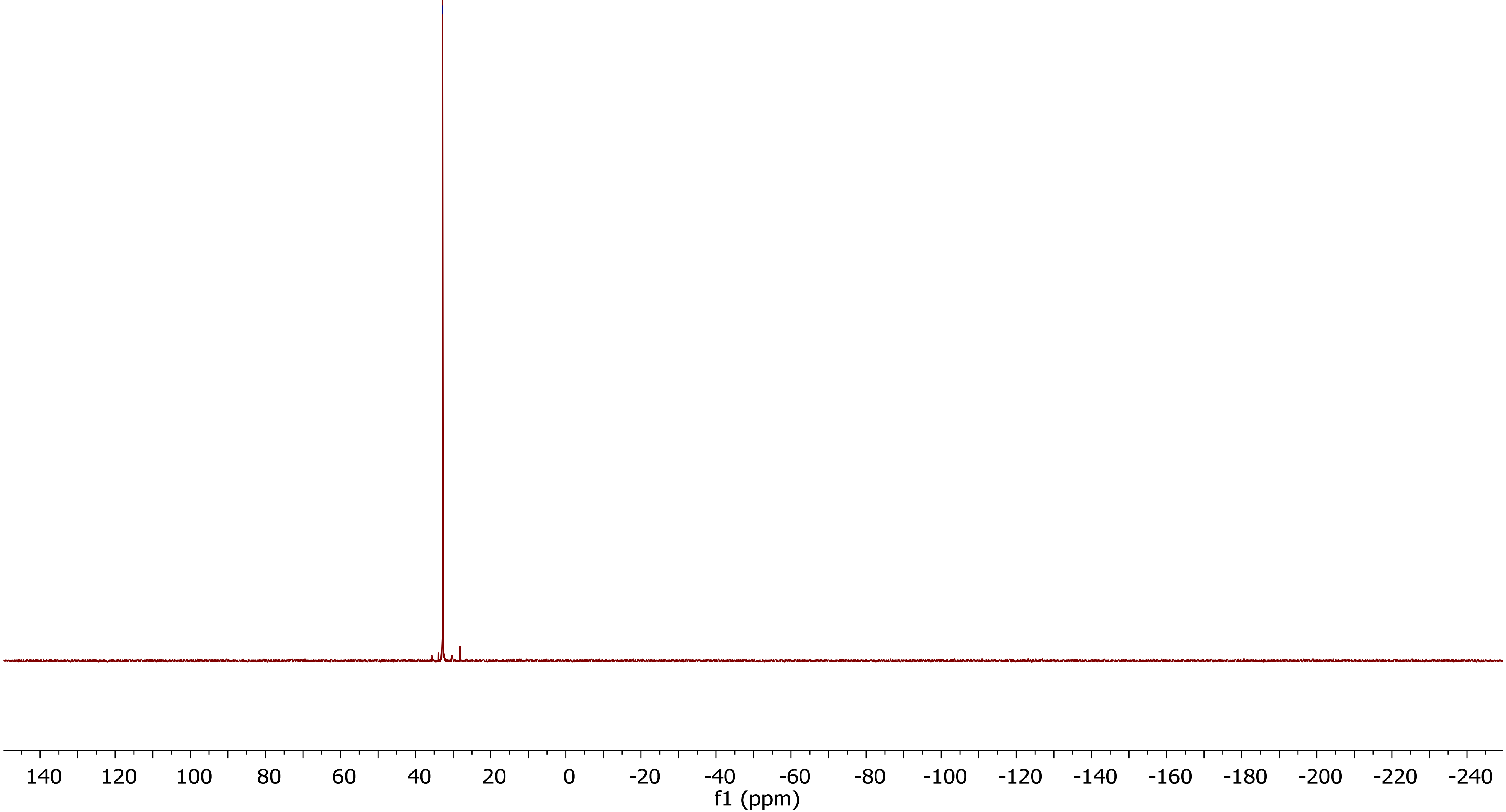

KA574.101.{1H}.1.fid
/ILDT KA574.101

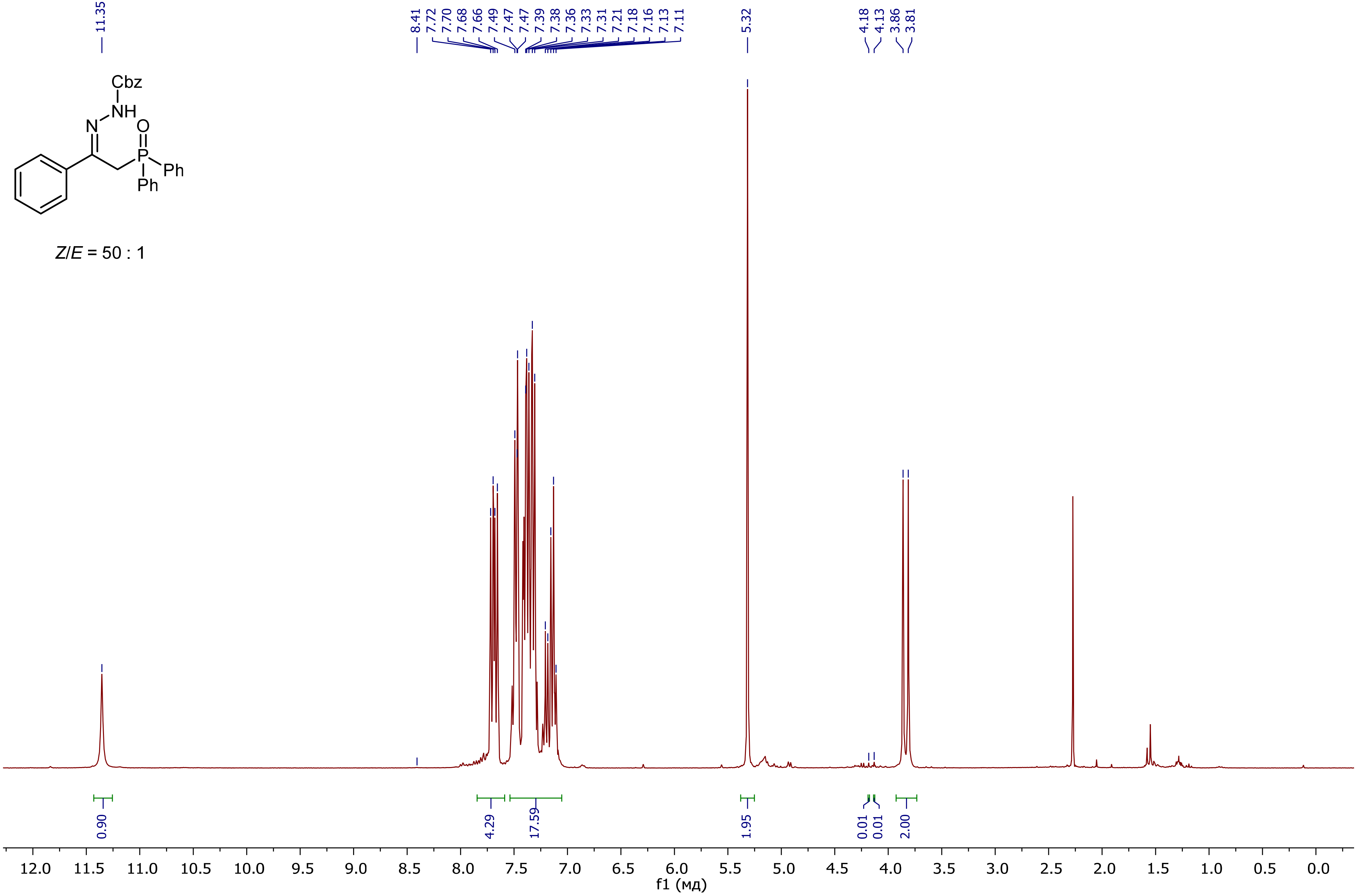

KA574.101.{13C}.2.fid
/ILDT KA574.101

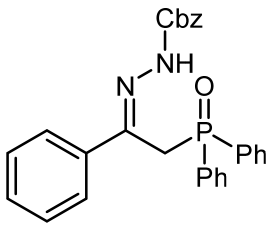

Z/*E* = 50 : 1

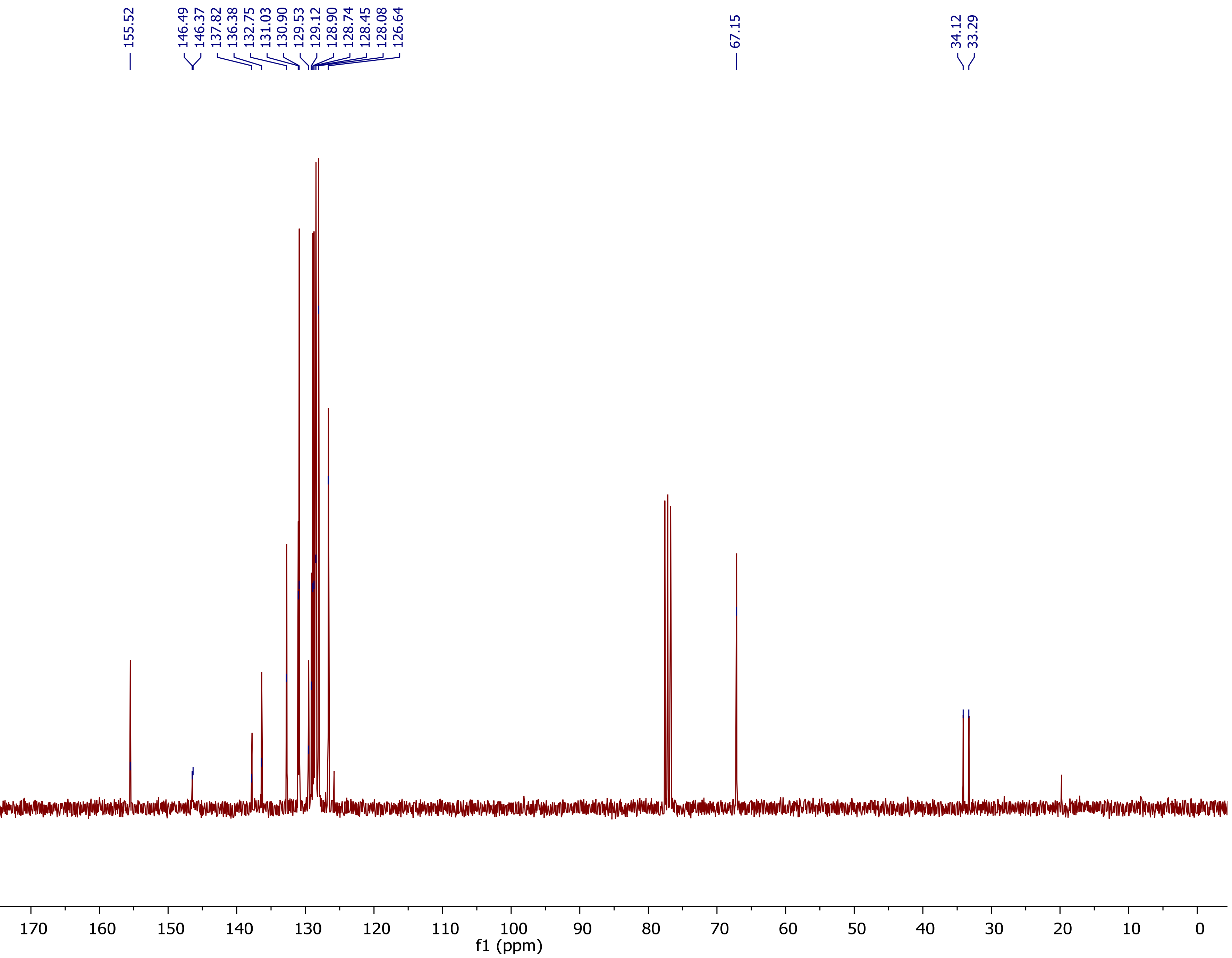

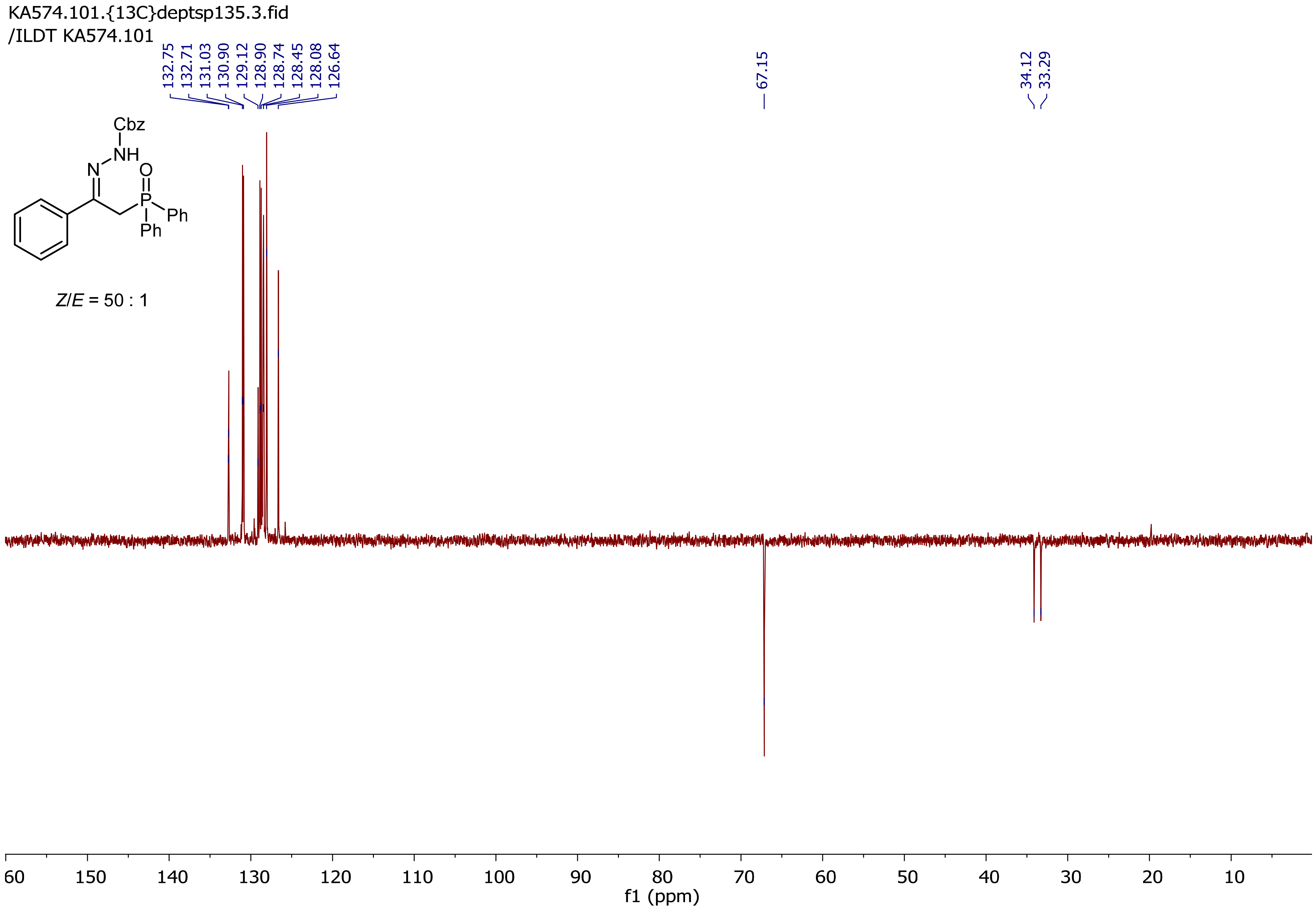
KA574.101.{13C}deptsp135.3.fid
/ILDT KA574.101
132.75
132.71
131.03
130.90
129.12
128.90
128.74
128.45
128.08
126.64
67.15
34.12
33.29
Cbz
NH
N
O
P
Ph
Ph
Z/E = 50 : 1
60
150
140
130
120
110
100
90
80
70
60
50
40
30
20
10
f1 (ppm)

KA574.101.{31P}INVGATED.4.fid
/ILDT KA574.101

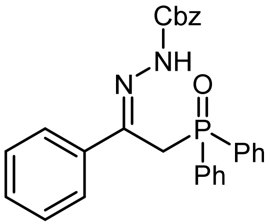

Z/*E* = 50 : 1

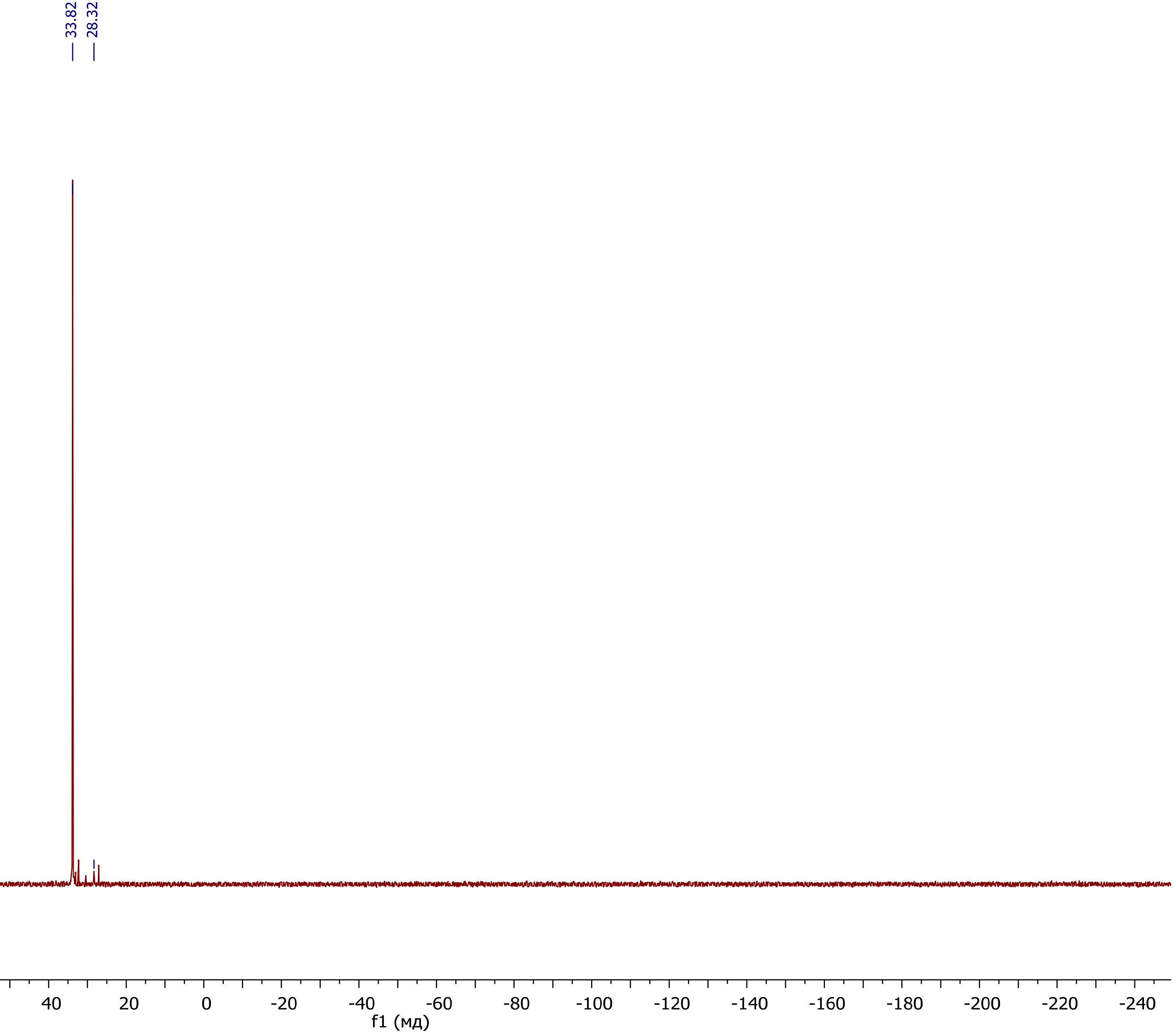

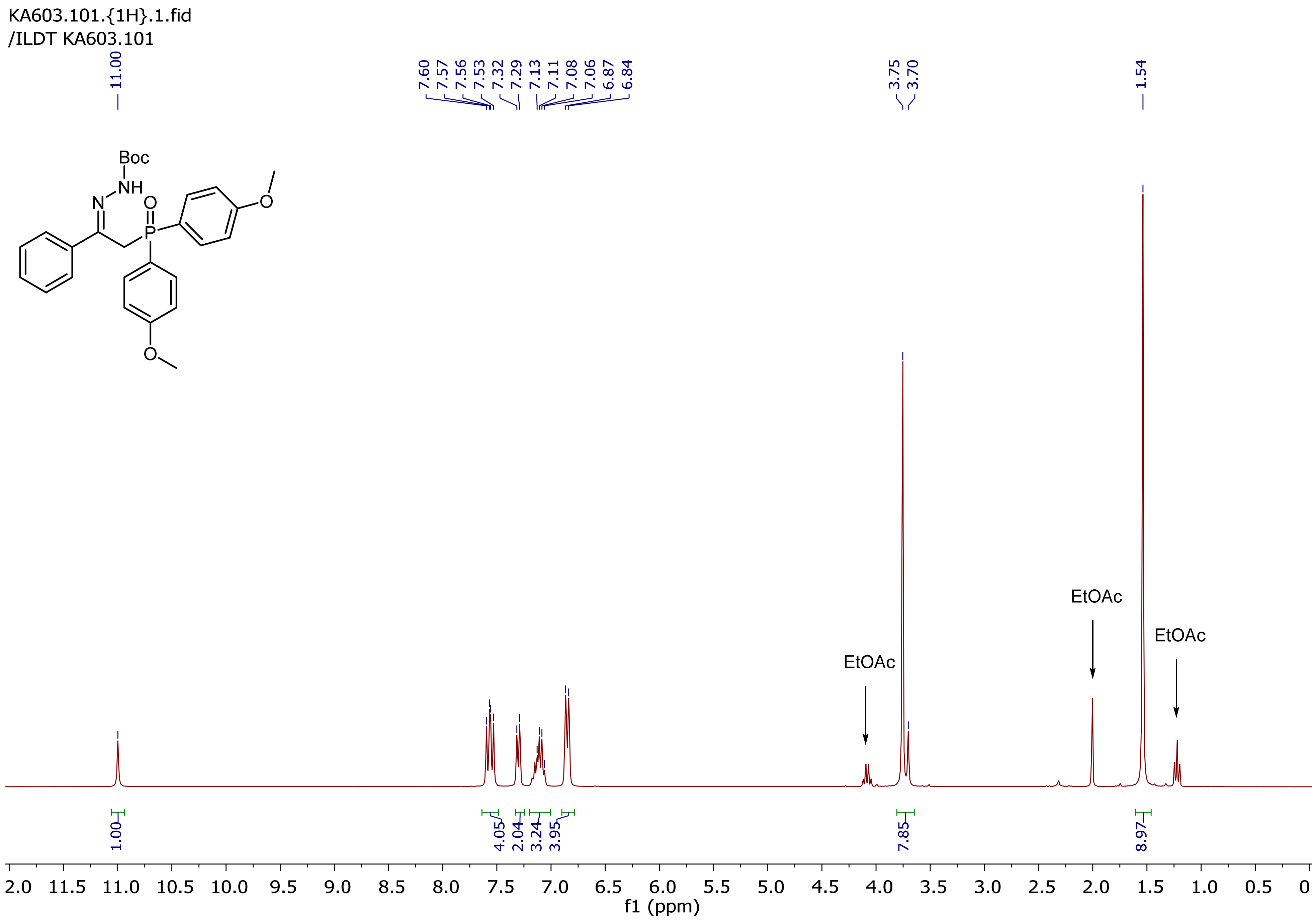

KA603.101.{1H}.1.fid
/ILDT KA603.101
11.00
7.60
7.57
7.56
7.53
7.32
7.29
7.13
7.11
7.08
7.06
6.87
6.84
3.75
3.70
1.54
Boc
NH
N
O
P
O
O
EtOAc
EtOAc
EtOAc
1.00
4.05
2.04
3.24
3.95
7.85
8.97
f1 (ppm)

KA603.101.{13C}.2.fid
/ILDT KA603.101

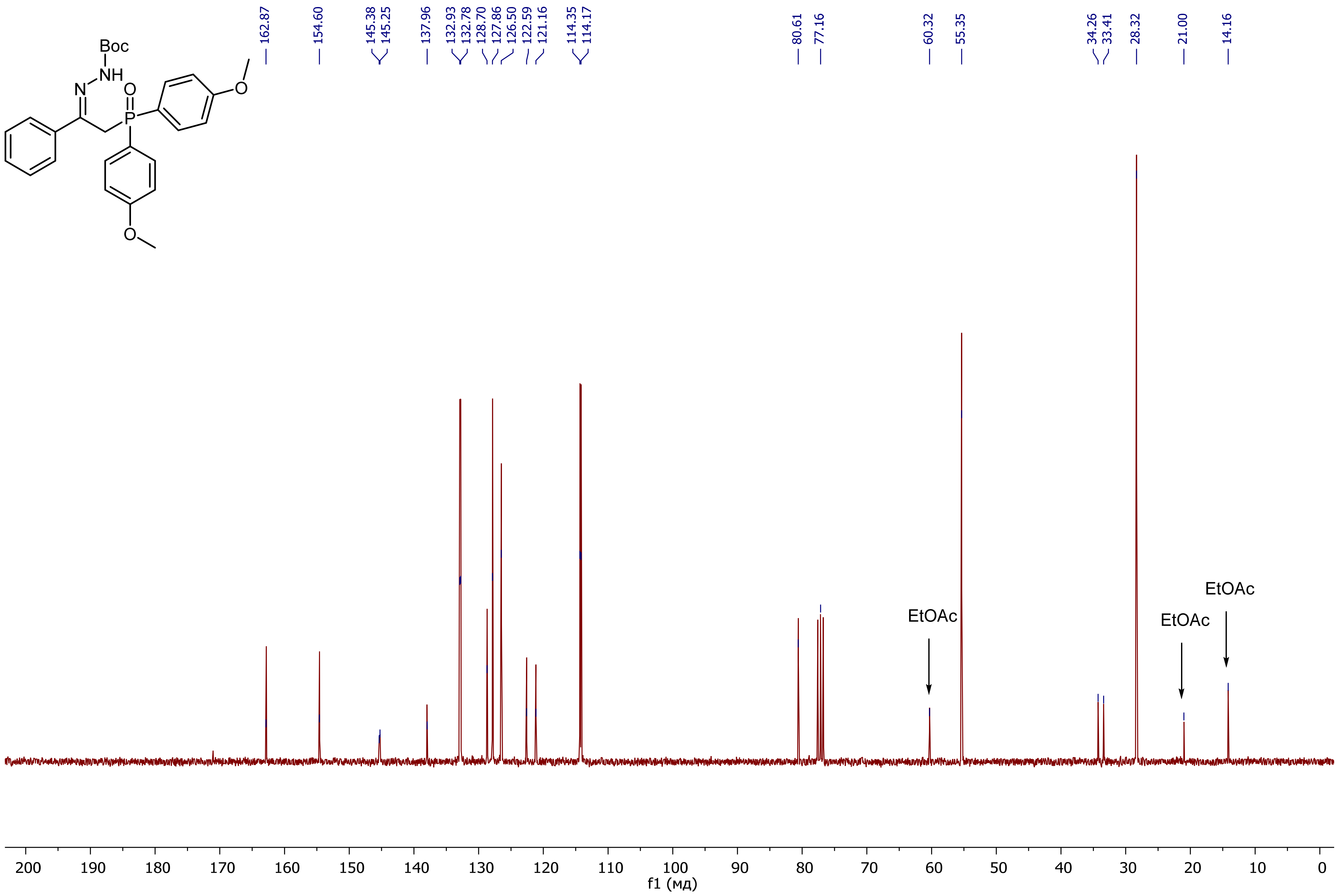

KA603.101.{13C}deptsp135.3.fid
/ILDT KA603.101

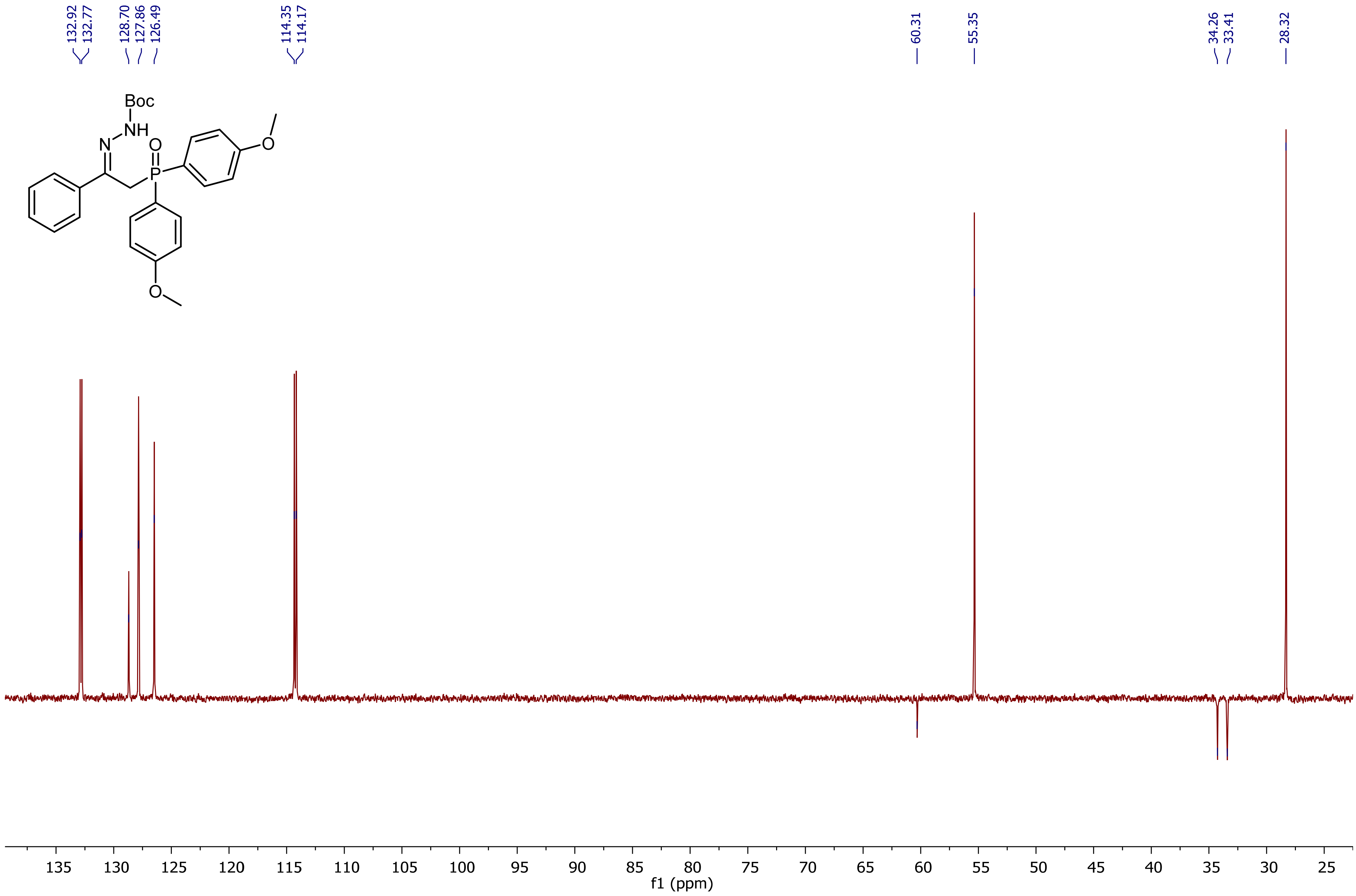

KA603.101.{31P}INVGATED.31.fid
/ILDT KA603.101

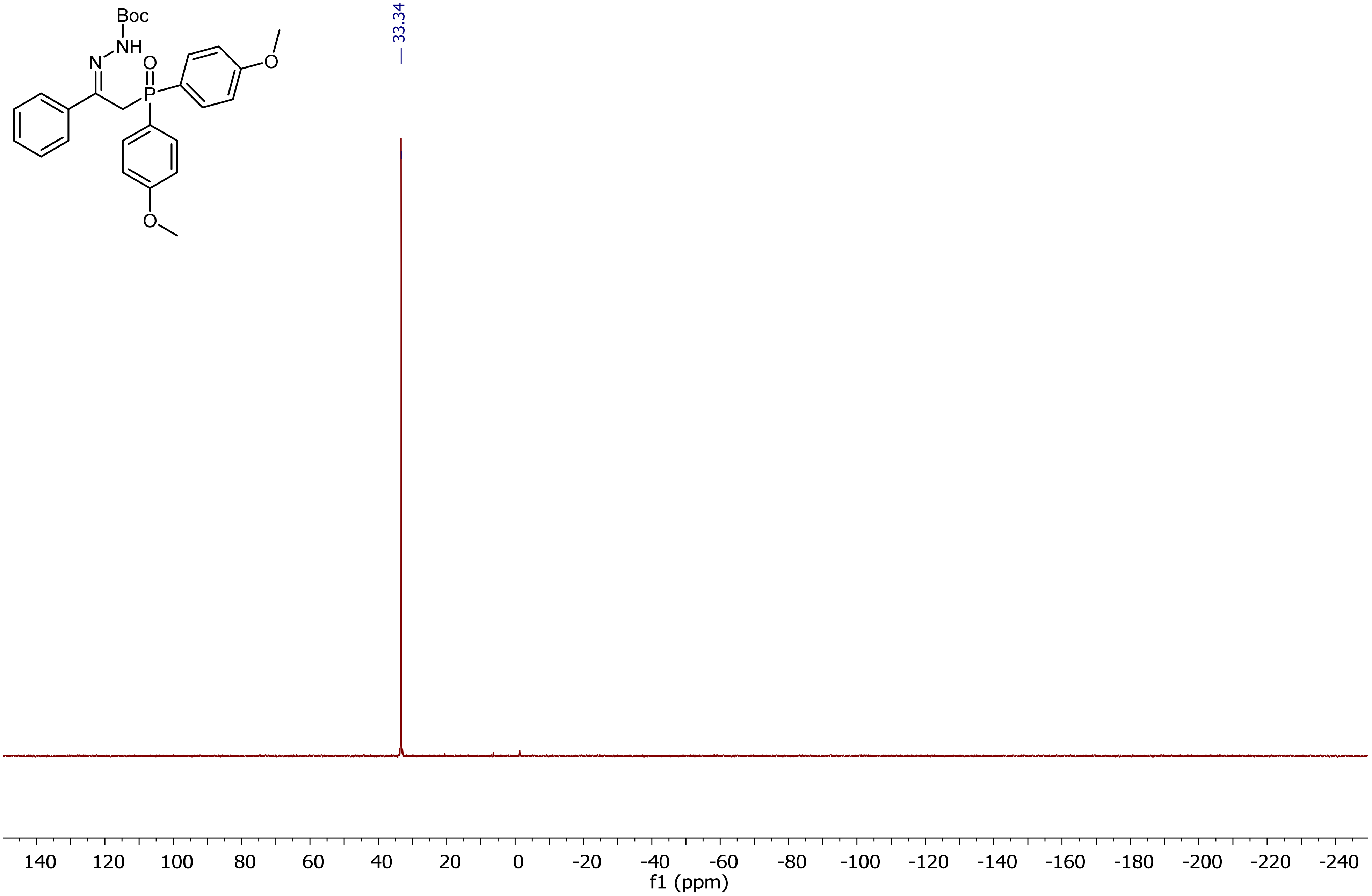

FT-IR

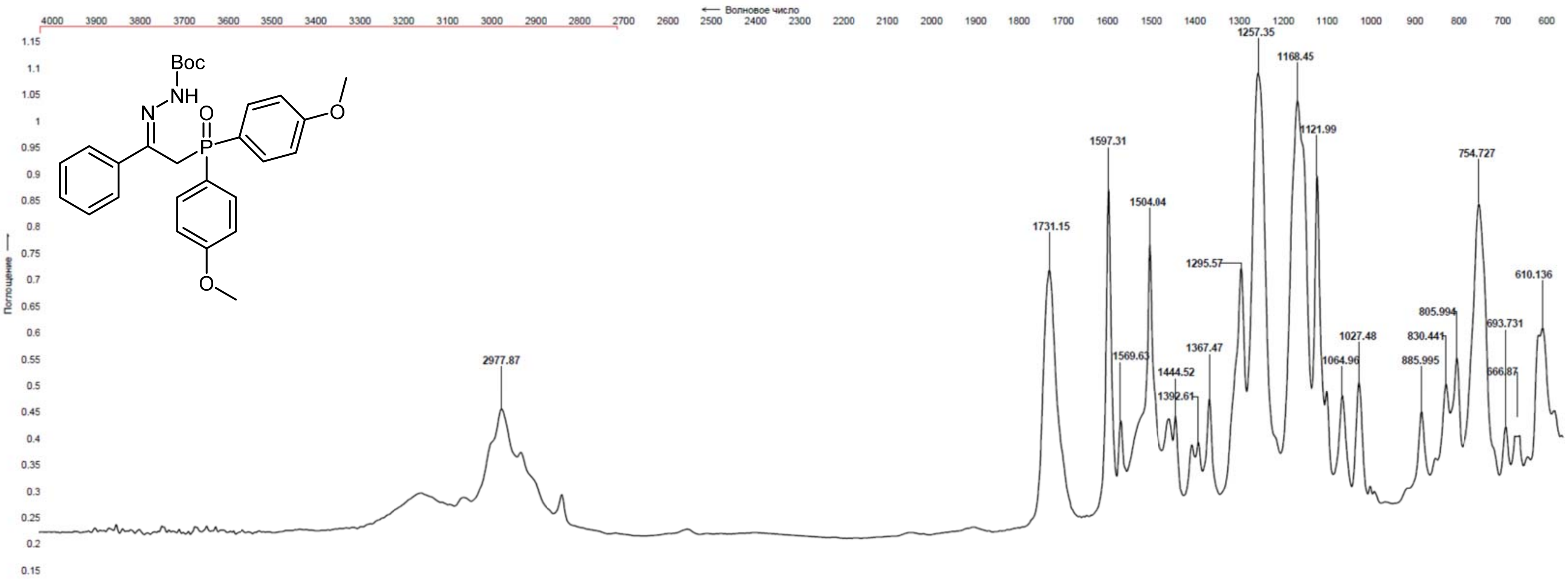
Boc
NH
N
O
P
O
O
Волновое число
Поглощение
2977.87
1731.15
1597.31
1569.63
1504.04
1444.52
1392.61
1367.47
1295.57
1257.35
1168.45
1121.99
1064.96
1027.48
885.995
830.441
805.994
754.727
693.731
666.87
610.136

KA604.101.{1H}.1.fid
/ILDT KA604.101

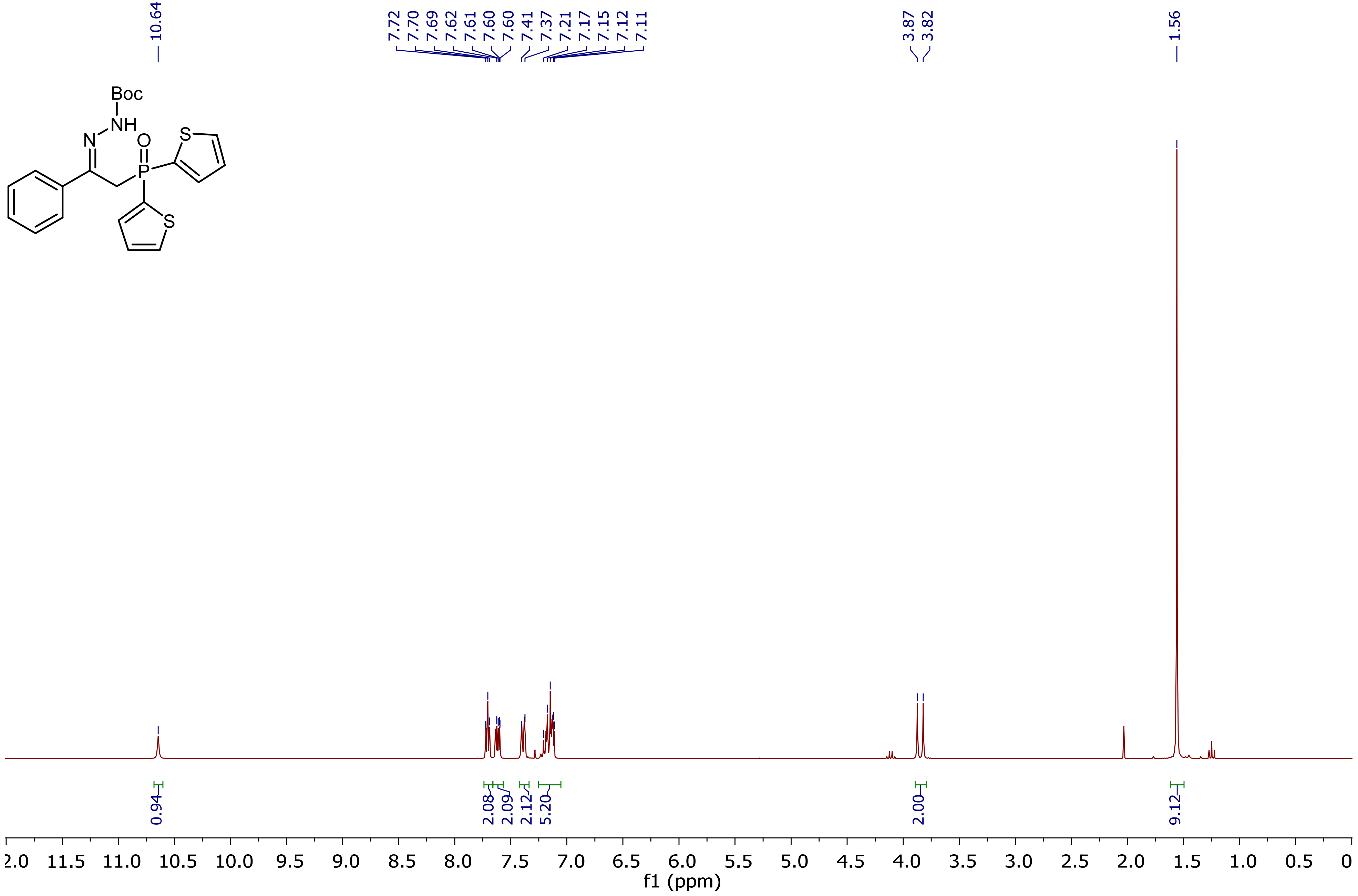

KA604.101.{13C}.2.fid
/ILDT KA604.101

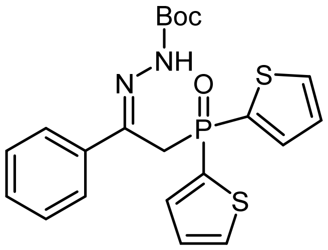

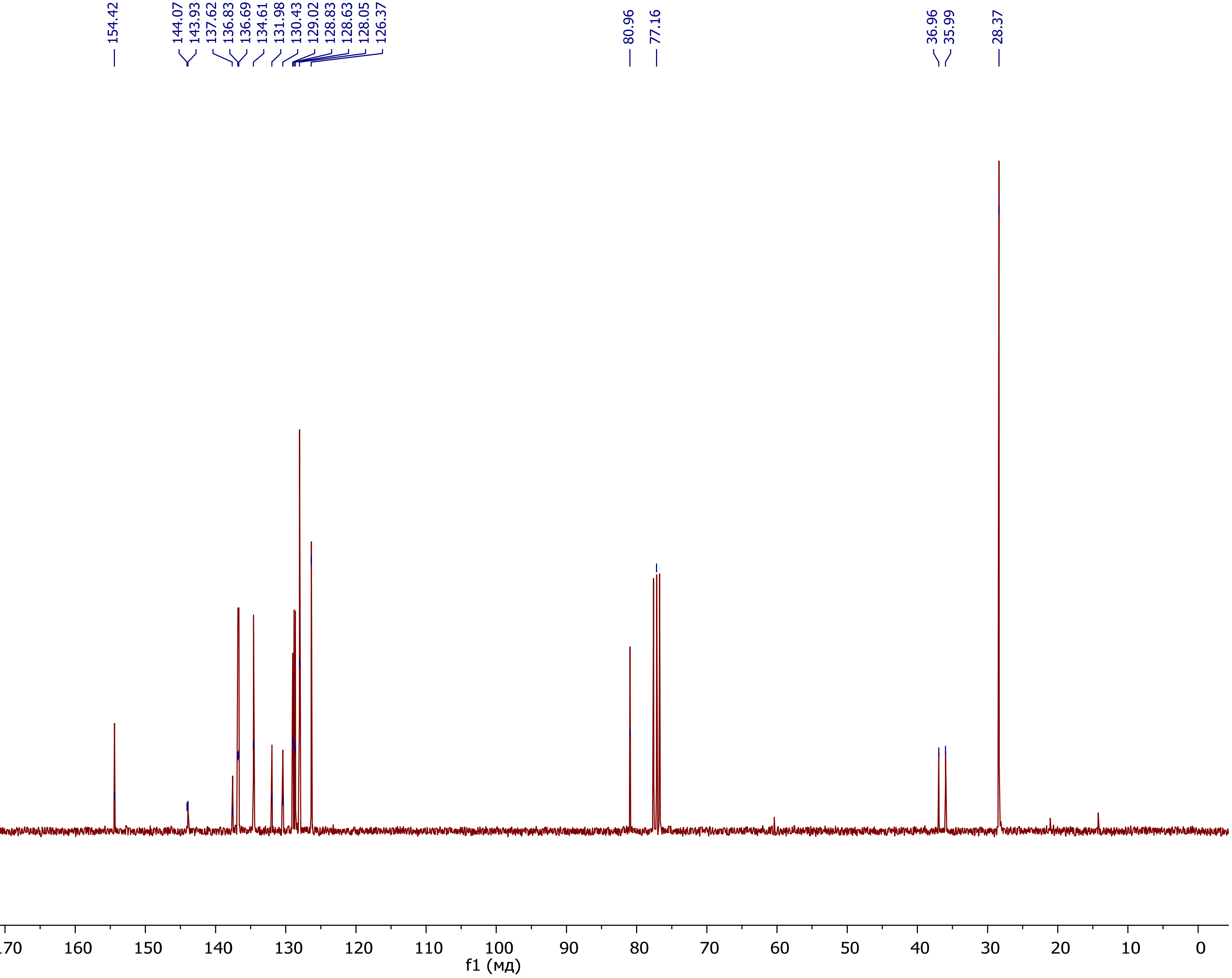

KA604.101.{13C}deptsp135.3.fid
/ILDT KA604.101

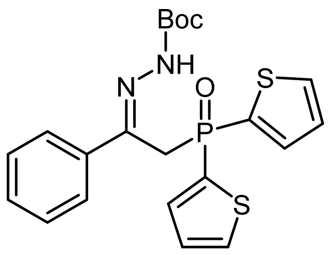

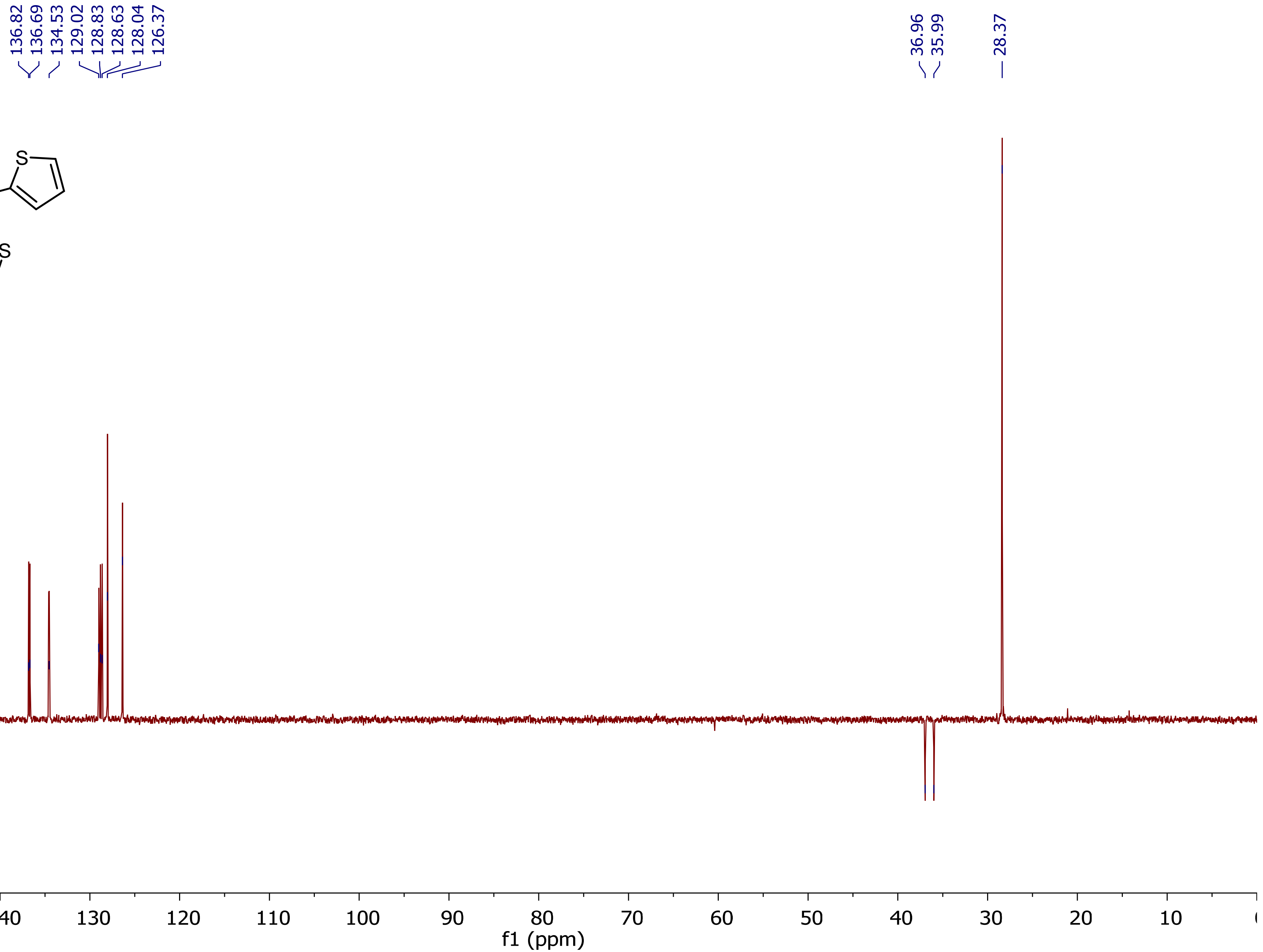

KA604.101.{31P}INVGATED.31.fid
/ILDT KA604.101

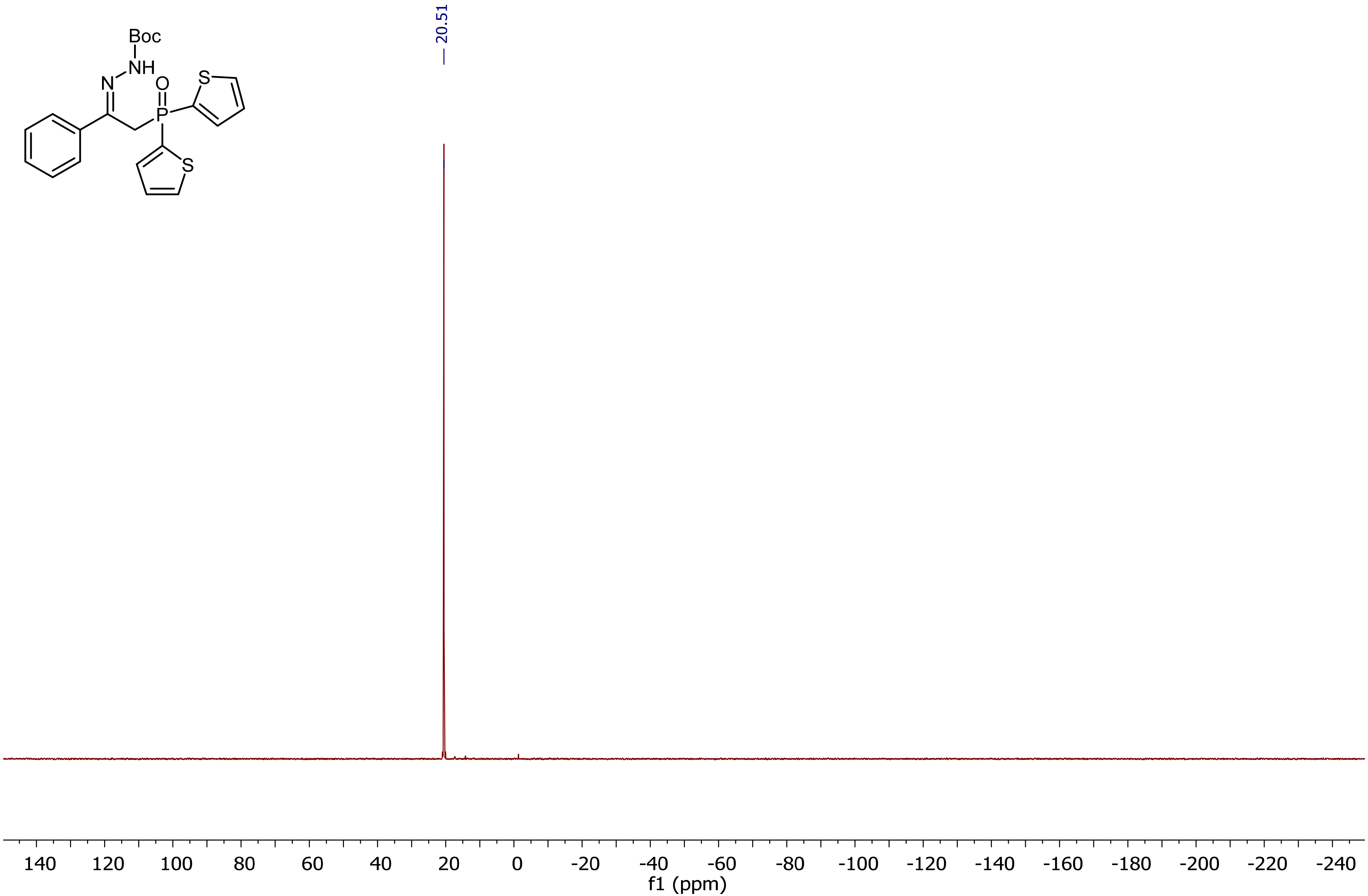

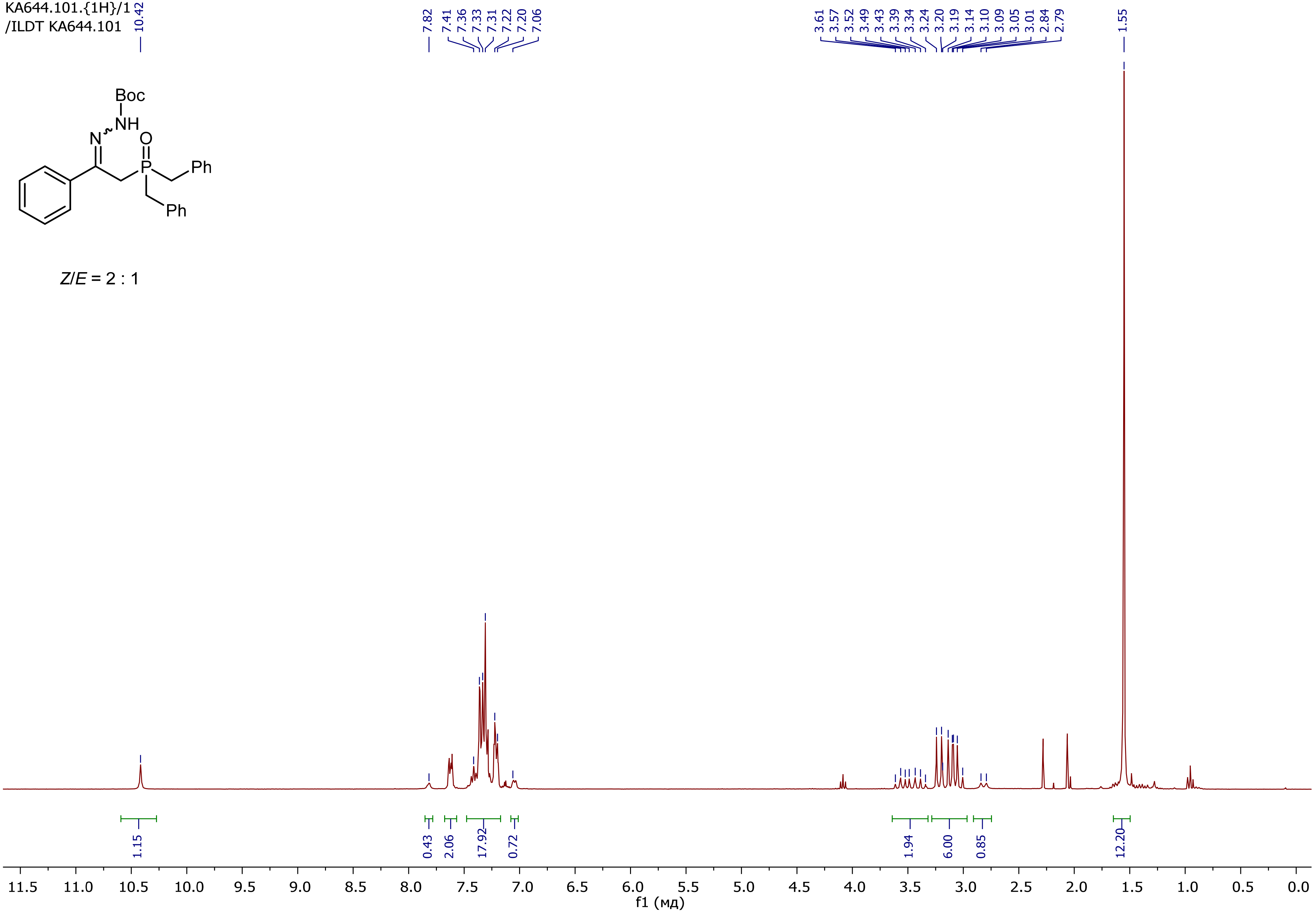
KA644.101.{1H}/1
/ILDT KA644.101
Boc
NH
N
O
P
Ph
Ph
Z/E = 2 : 1
10.42
7.82
7.41
7.36
7.33
7.31
7.22
7.20
7.06
3.61
3.57
3.52
3.49
3.43
3.39
3.34
3.24
3.20
3.19
3.14
3.10
3.09
3.05
3.01
2.84
2.79
1.55
1.15
0.43
2.06
17.92
0.72
1.94
6.00
0.85
12.20
11.5 11.0 10.5 10.0 9.5 9.0 8.5 8.0 7.5 7.0 6.5 6.0 5.5 5.0 4.5 4.0 3.5 3.0 2.5 2.0 1.5 1.0 0.5 0.0
f1 (мд)

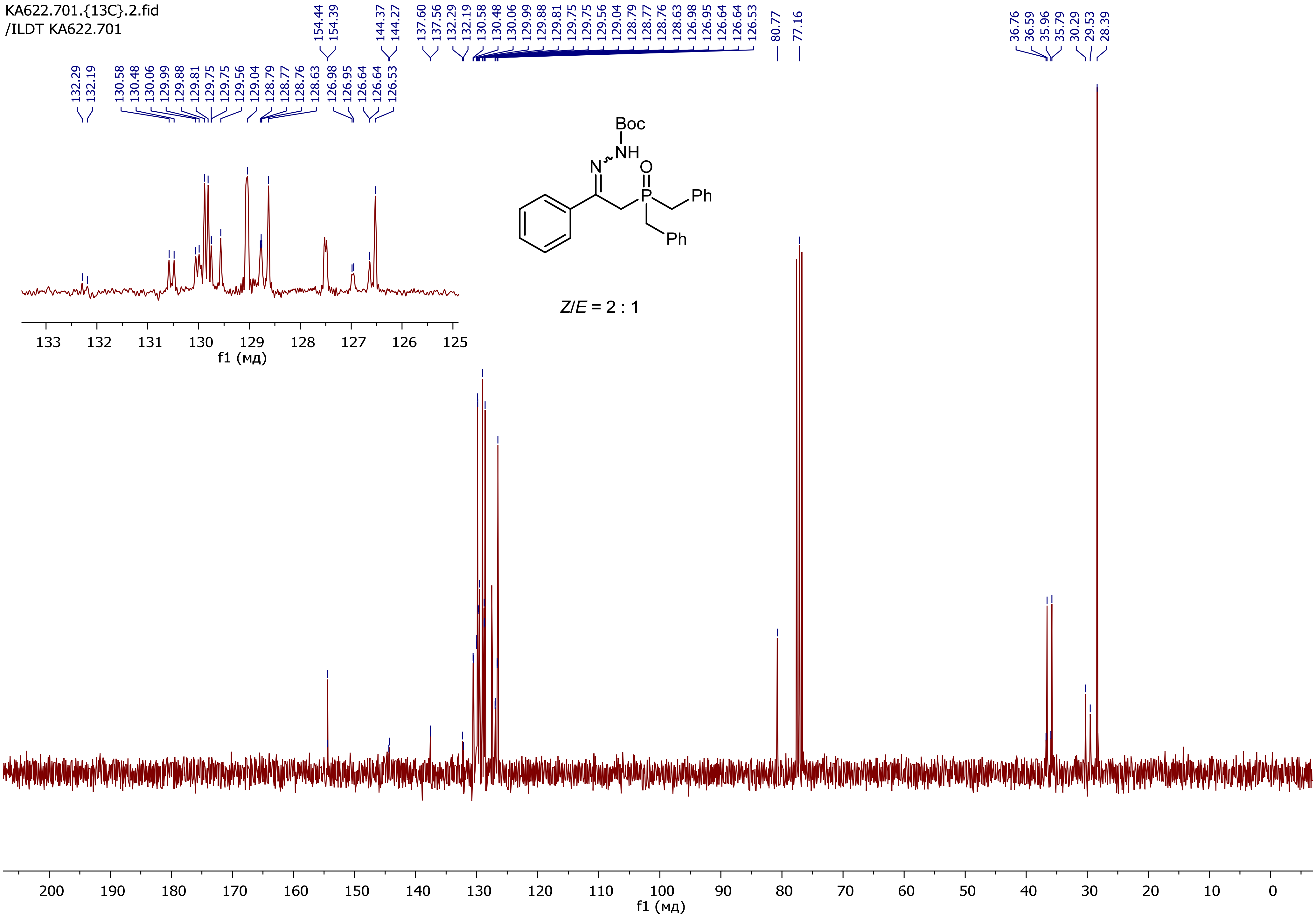
KA622.701.{13C}.2.fid
/ILDT KA622.701
154.44
154.39
144.37
144.27
137.60
137.56
132.29
132.19
130.58
130.48
130.06
129.99
129.88
129.81
129.75
129.75
129.56
129.04
128.79
128.77
128.76
128.63
126.98
126.95
126.64
126.64
126.53
80.77
77.16
36.76
36.59
35.96
35.79
30.29
29.53
28.39
Boc
NH
N
O
P
Ph
Ph
Z/E = 2 : 1
f1 (мд)
f1 (мд)

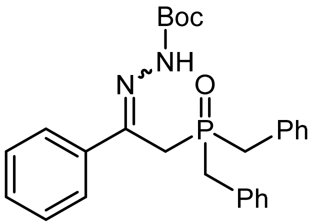
Boc
NH
N
O
P
Ph
Ph

Z/*E* = 2 : 1

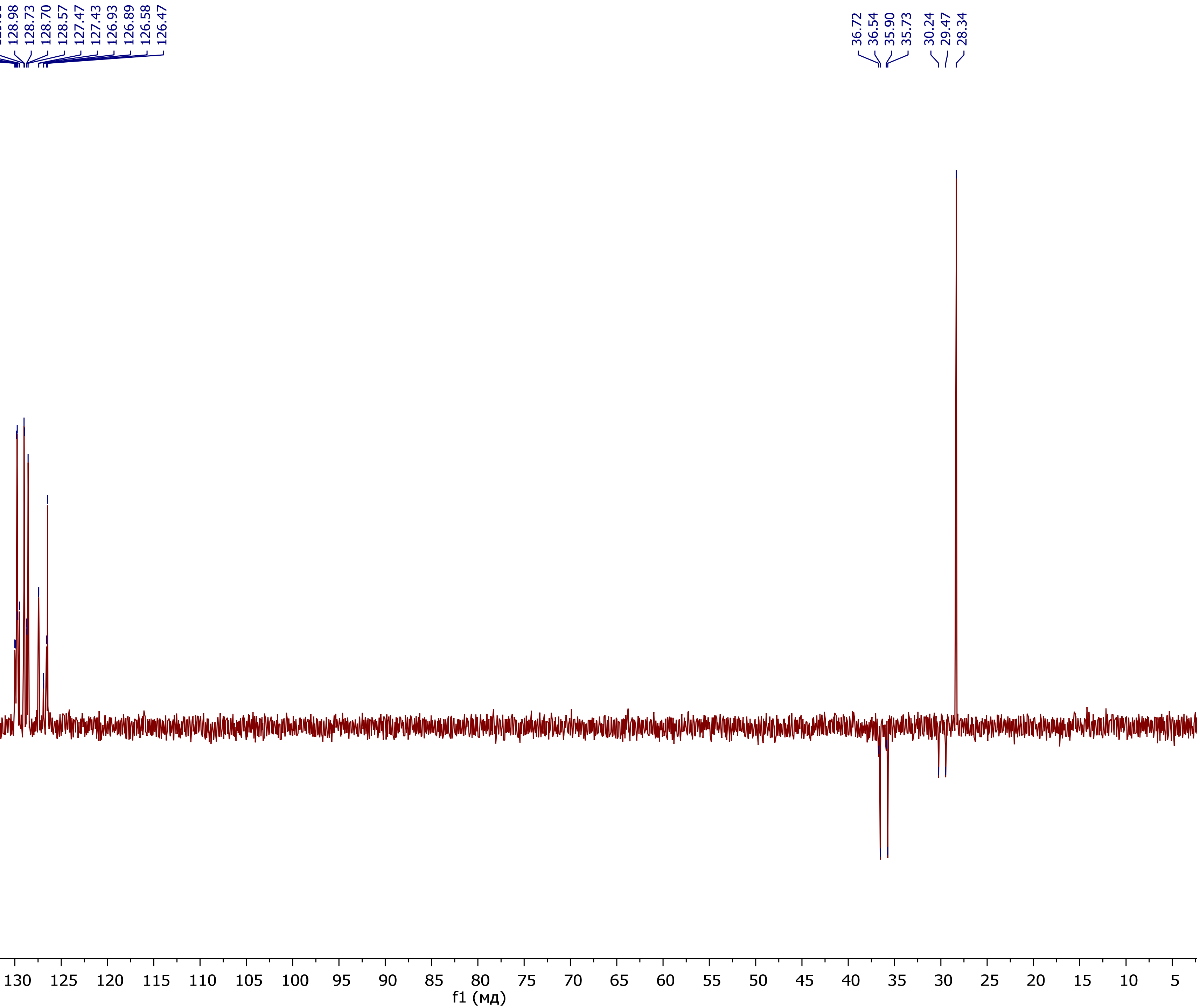
130.00
129.93
129.83
129.76
129.69
129.51
129.01
128.98
128.73
128.70
128.57
127.47
127.43
126.93
126.89
126.58
126.47
36.72
36.54
35.90
35.73
30.24
29.47
28.34
155 150 145 140 135 130 125 120 115 110 105 100 95 90 85 80 75 70 65 60 55 50 45 40 35 30 25 20 15 10 5
f1 (мд)

KA622.701.{31P}INVGATED.31.fid
/ILDT KA622.701

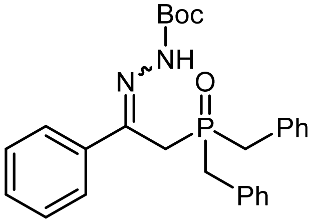

Z/*E* = 2 : 1

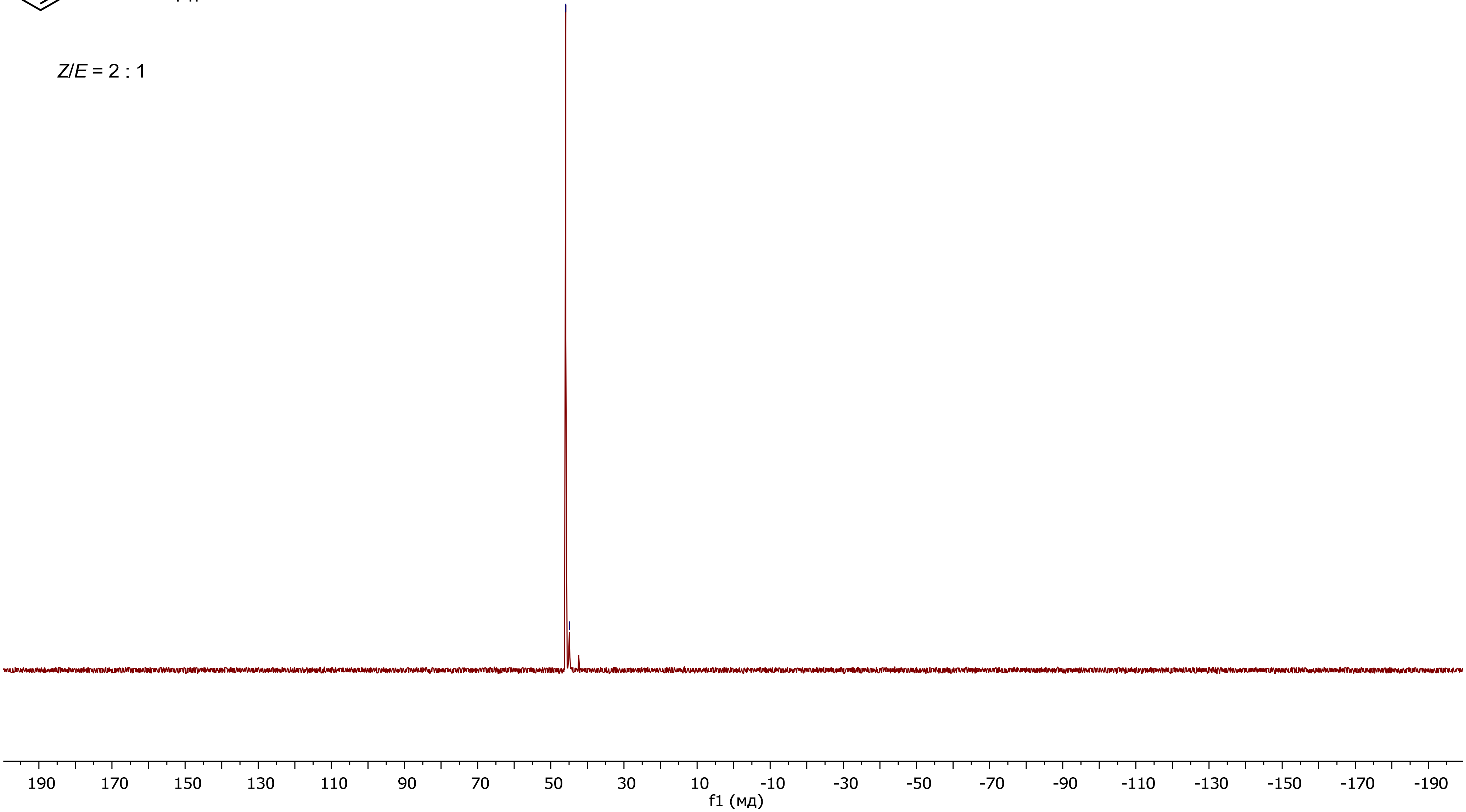

KA631.701-2-333K.{1H}.1.fid
/ILDT KA631.701-2-333K

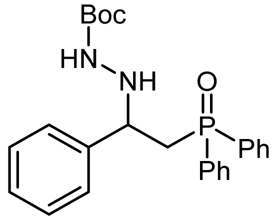

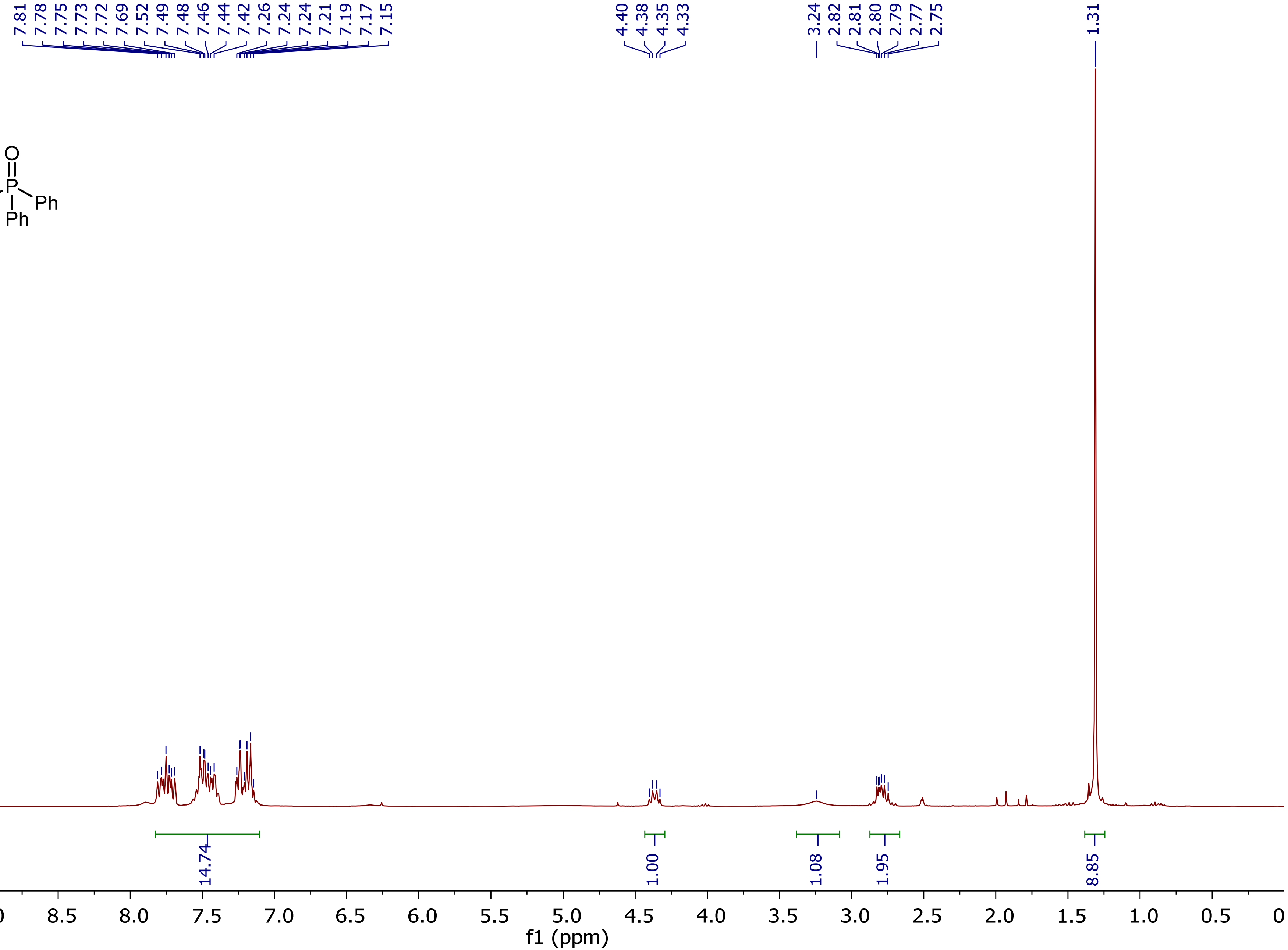

KA631.701-2-333K.{13C}.2.fid
/ILDT KA631.701-2-333K

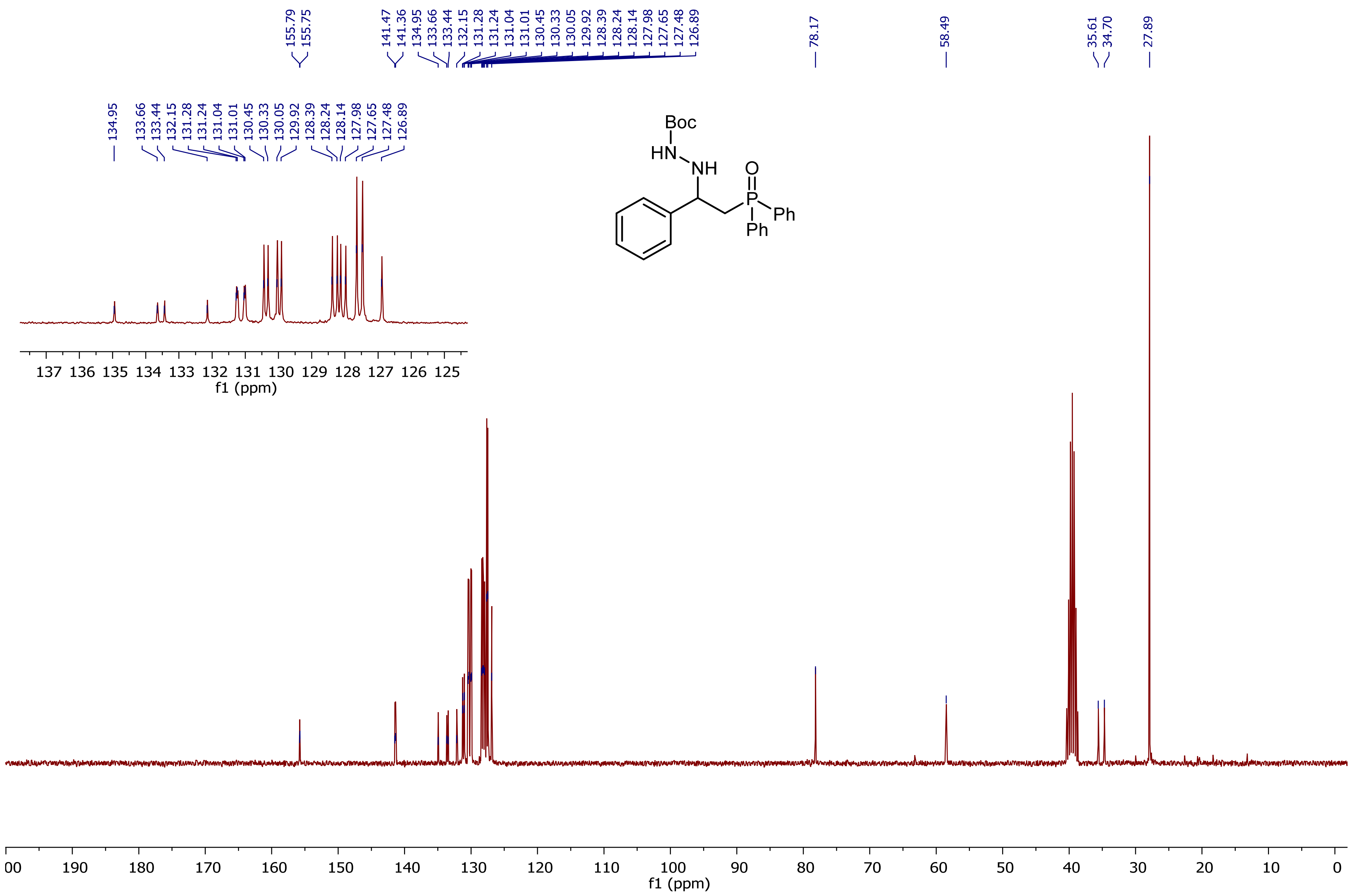

KA631.701-2-333K.{13C}deptsp135.3.fid
/ILDT KA631.701-2-333K

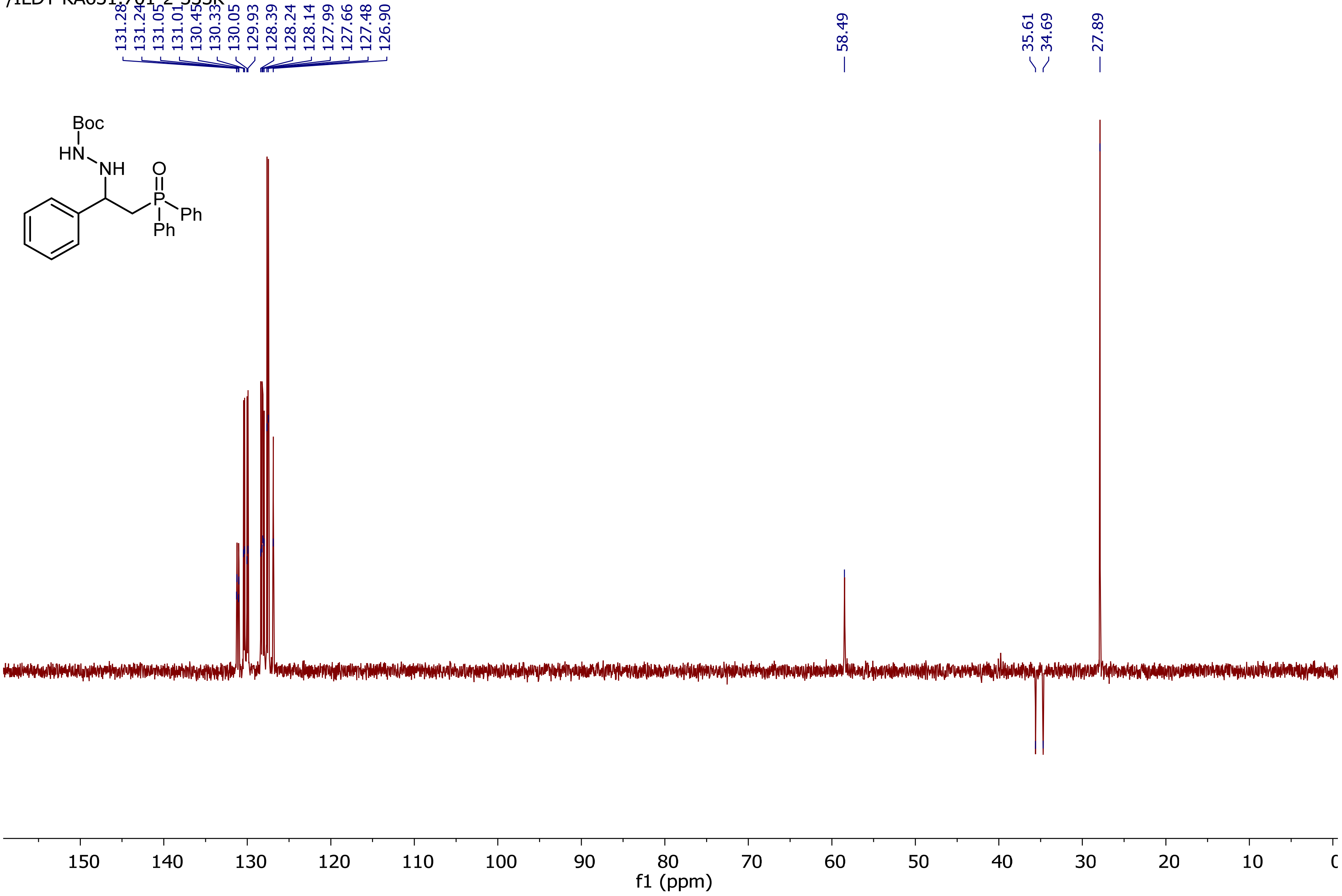

KA631.701-2-333K.{31P}INVGATED.31.fid
/ILDT KA631.701-2-333K

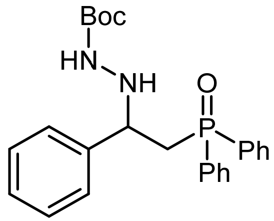

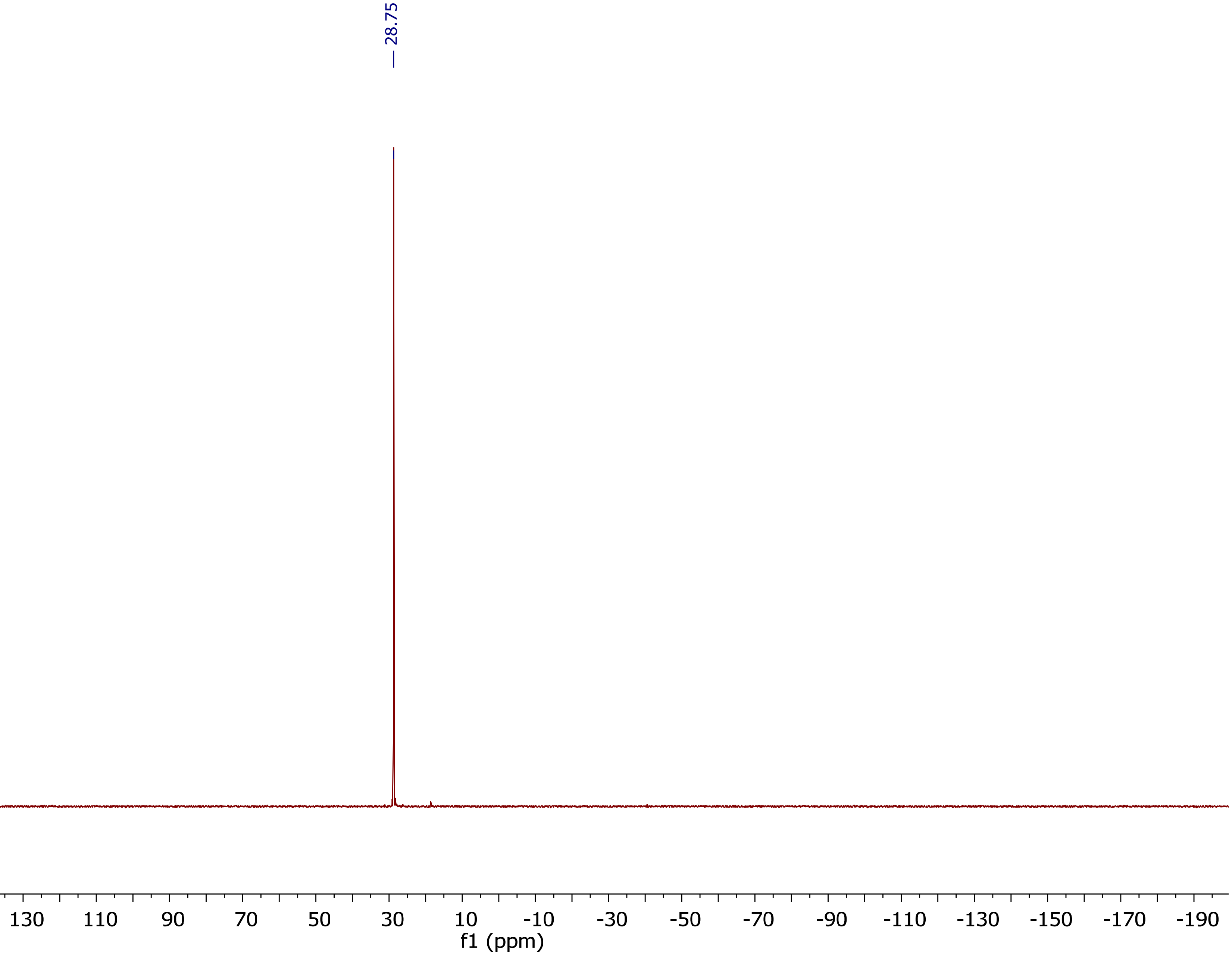

KA635.101-1.{1H}/1
/ILDT KA635.101-1

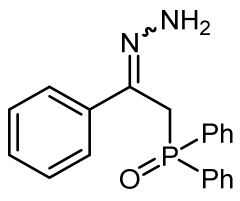

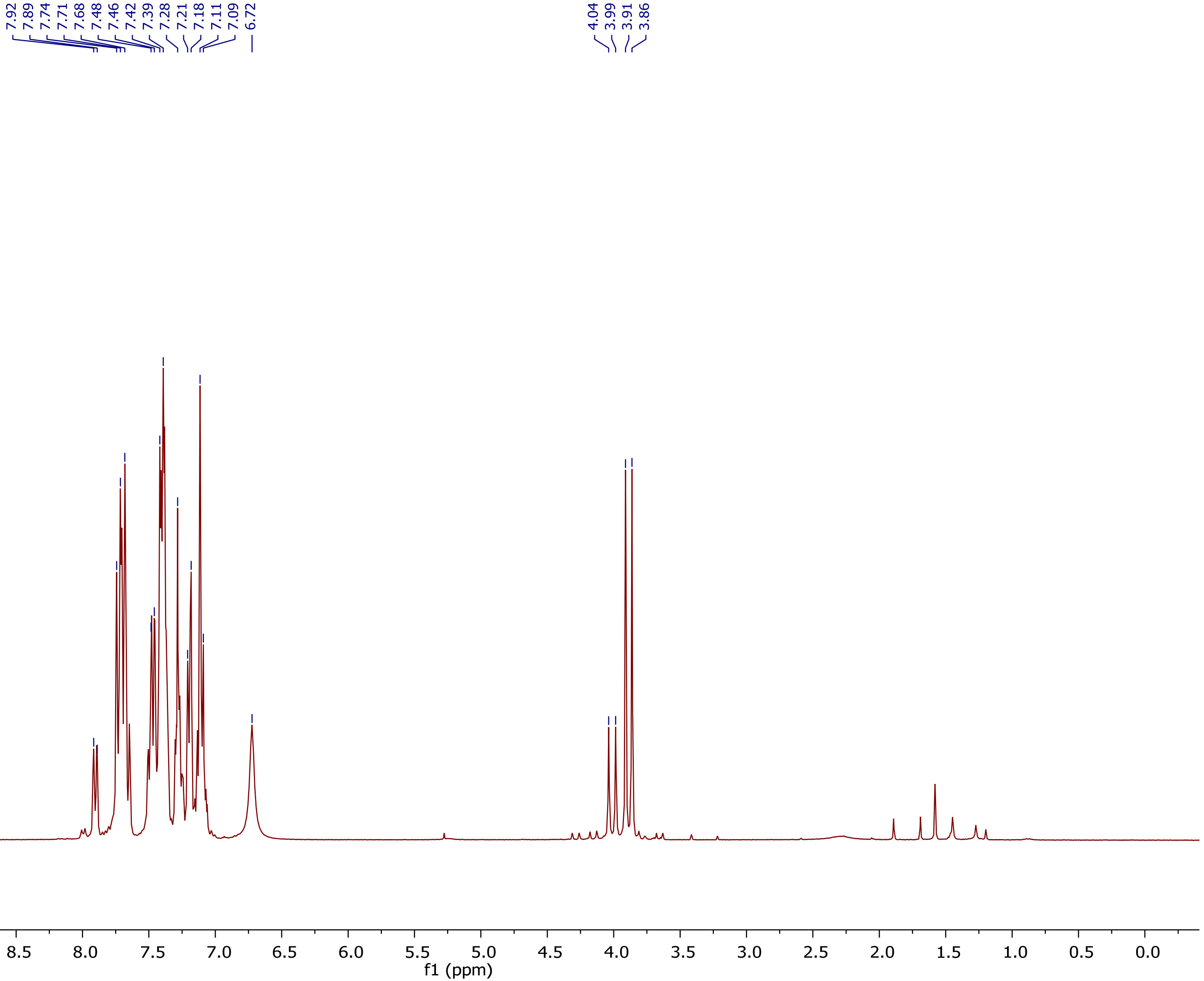

KA635.101-1.{13C}/2
/ILDT KA635.101-1

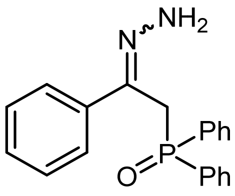

Z/*E* = 2 : 1

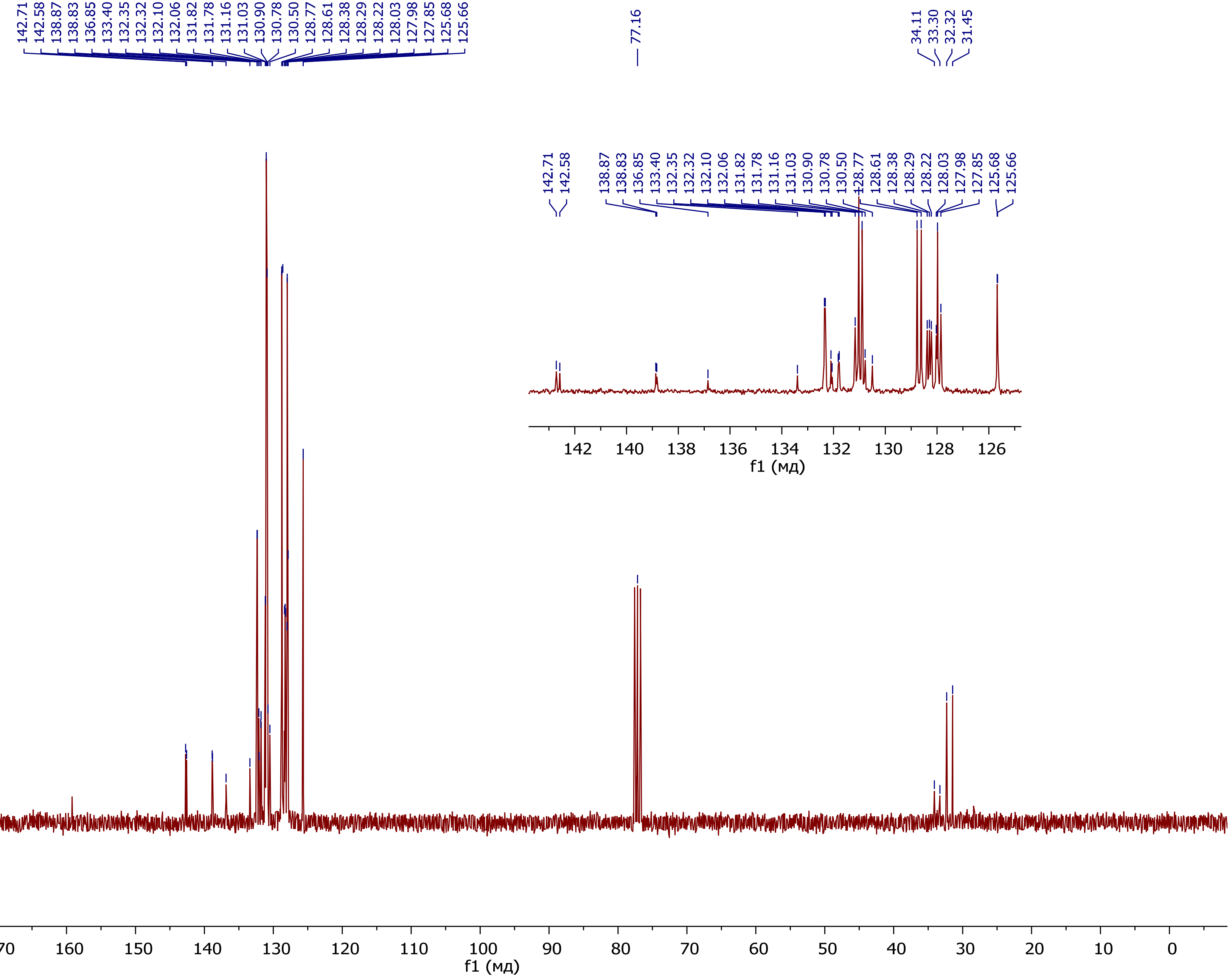

KA635.101-1.{13C}deptsp135/3
/ILDT KA635.101-1

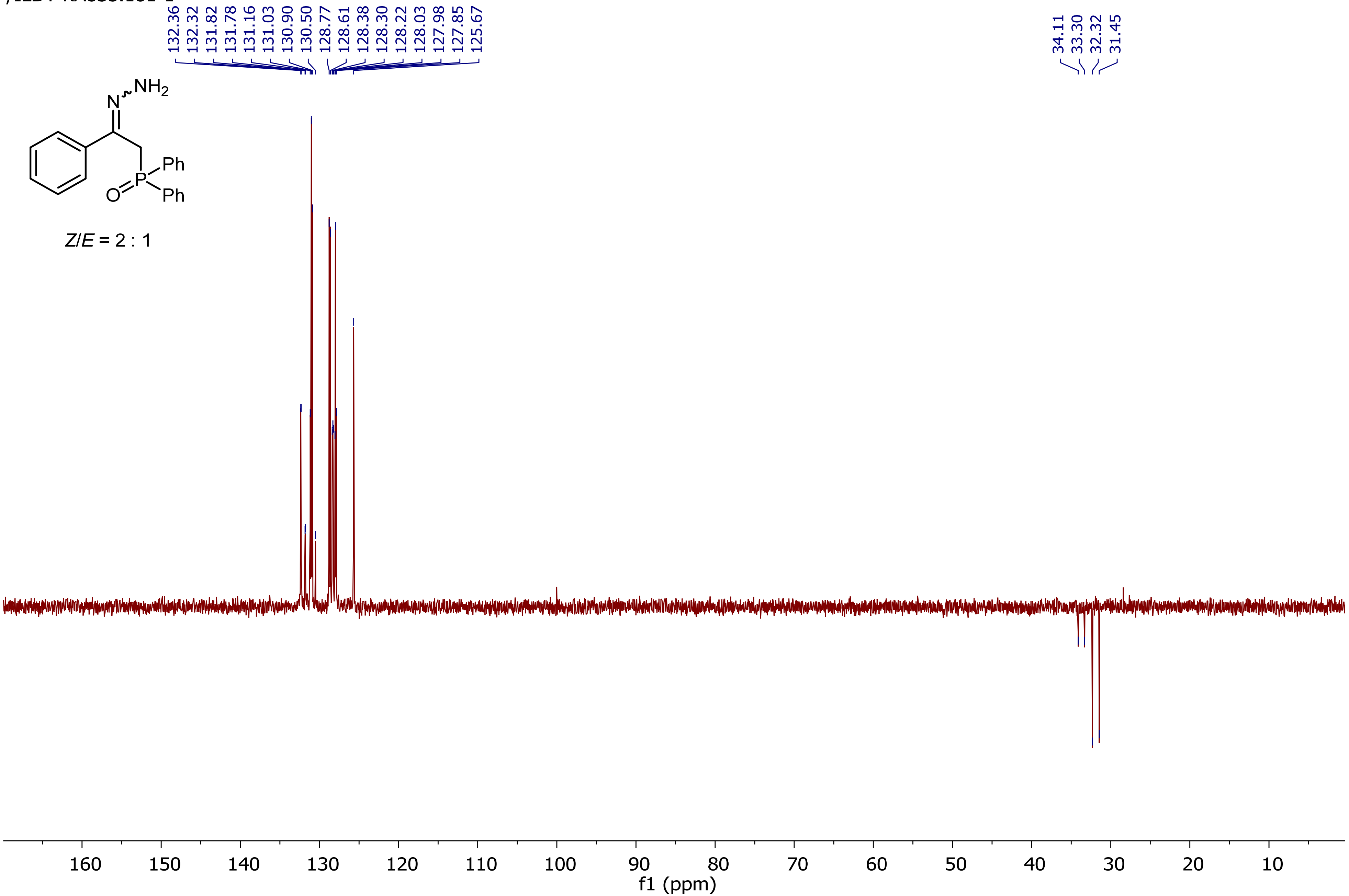

KA635.101-1.{31P}INVGATED/4
/ILDT KA635.101-1

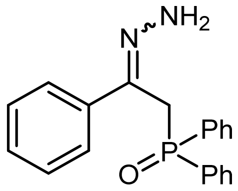

Z/*E* = 2 : 1

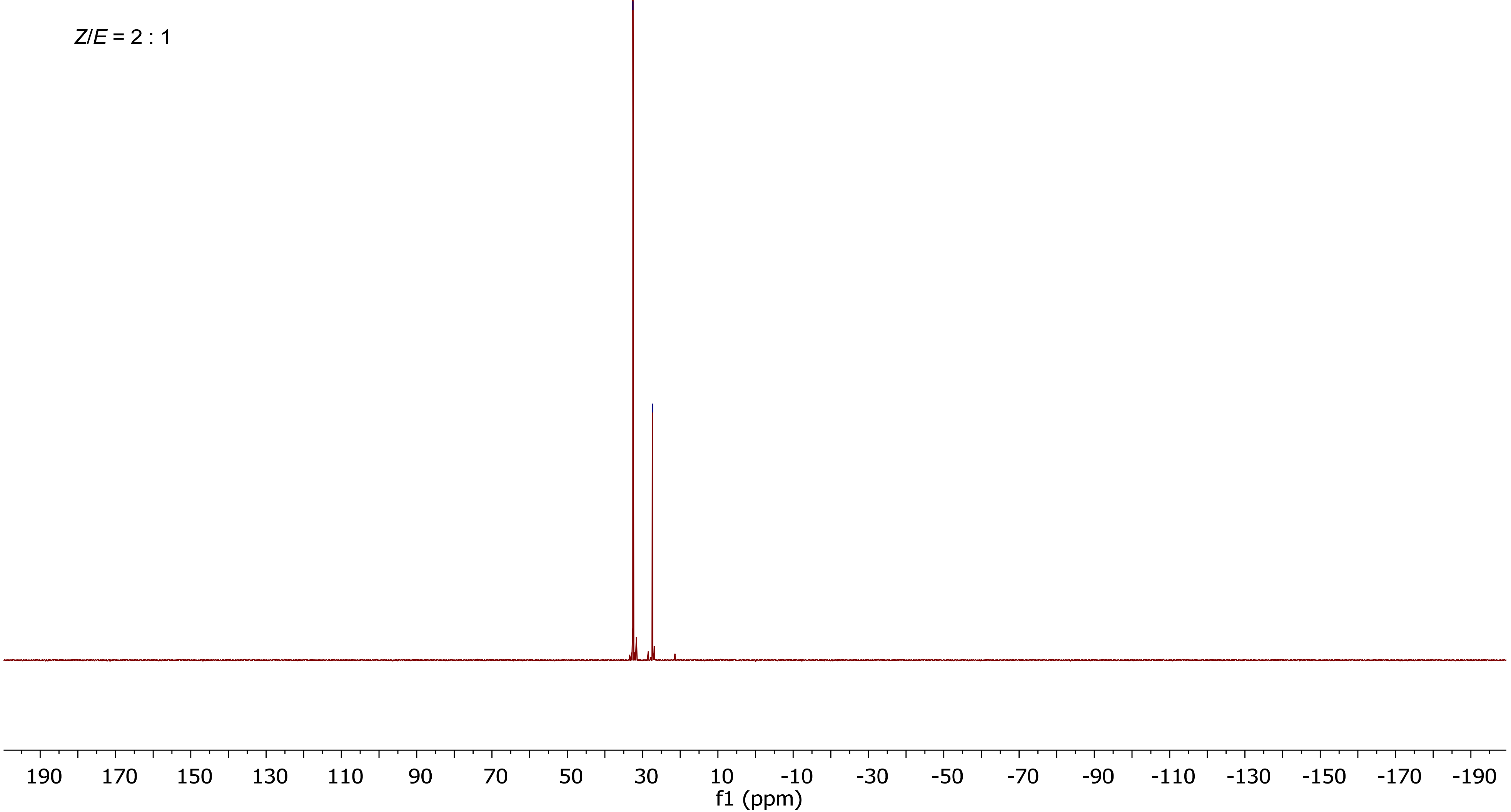

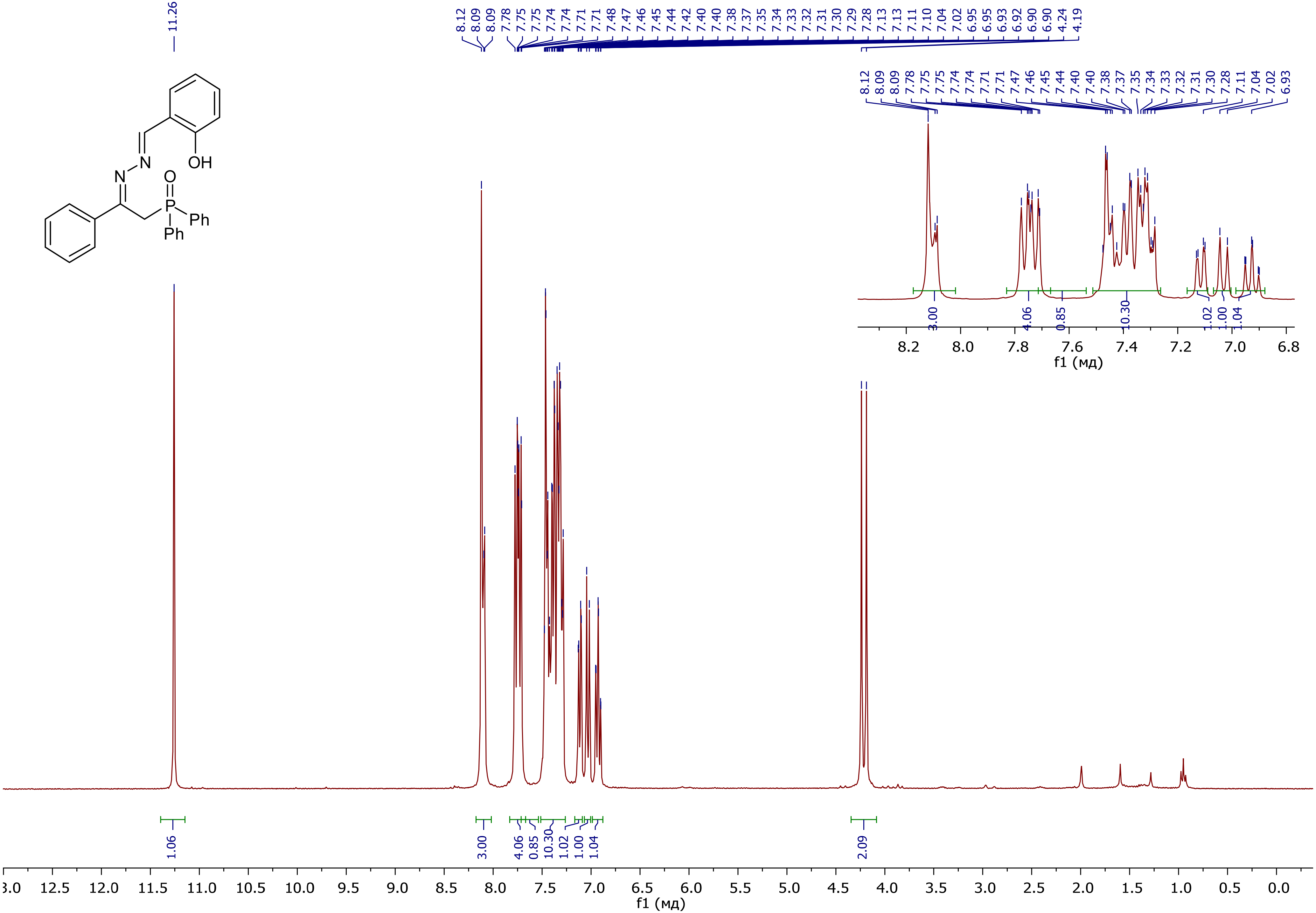

OH
N
N
O
P
Ph
Ph
f1 (мд)

KA741.111-1.{13C}/2
/ILDT KA741.111-1

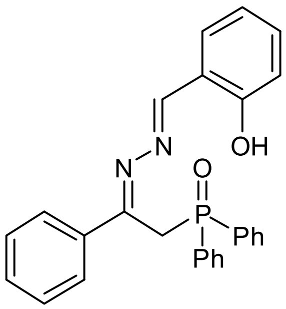

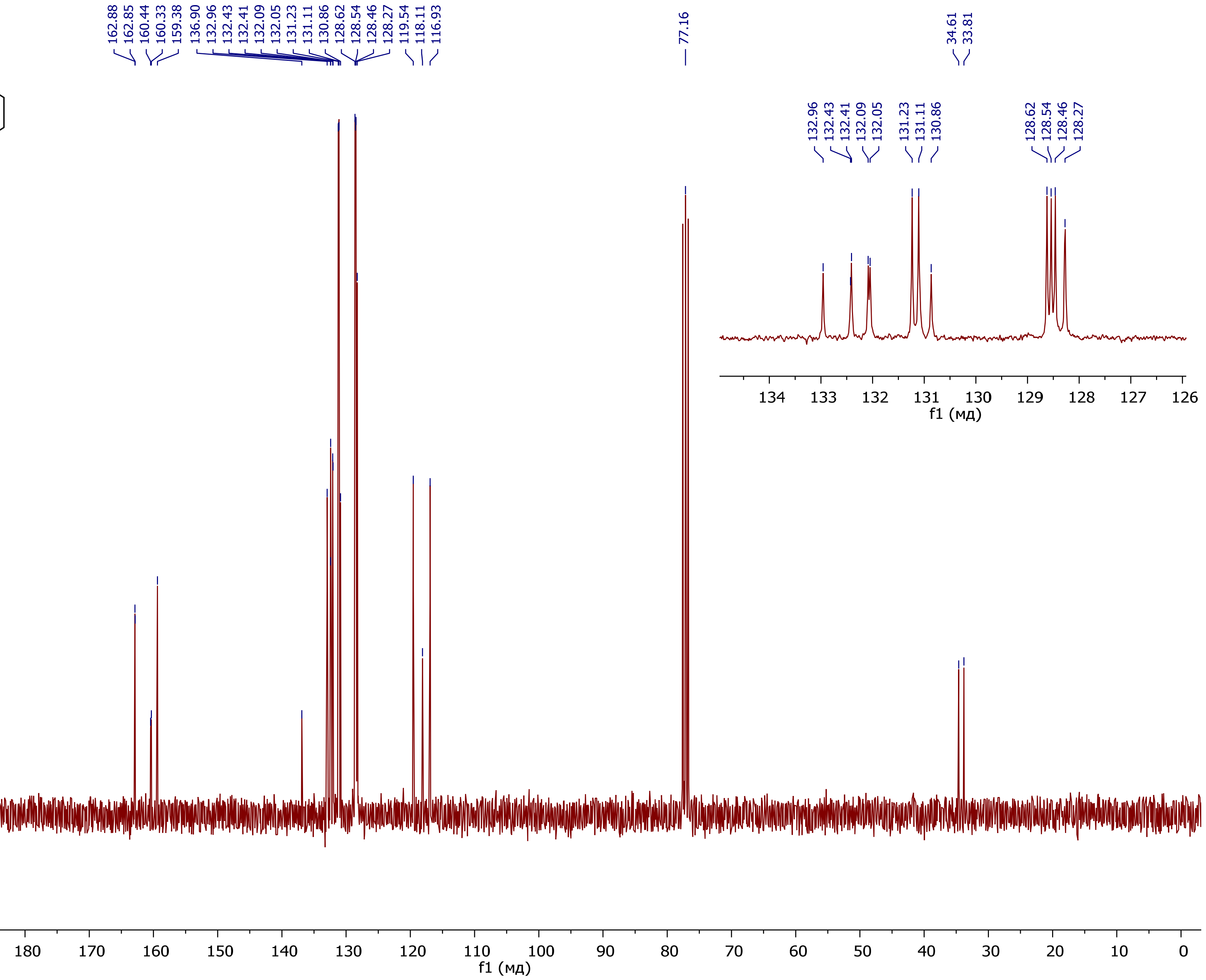

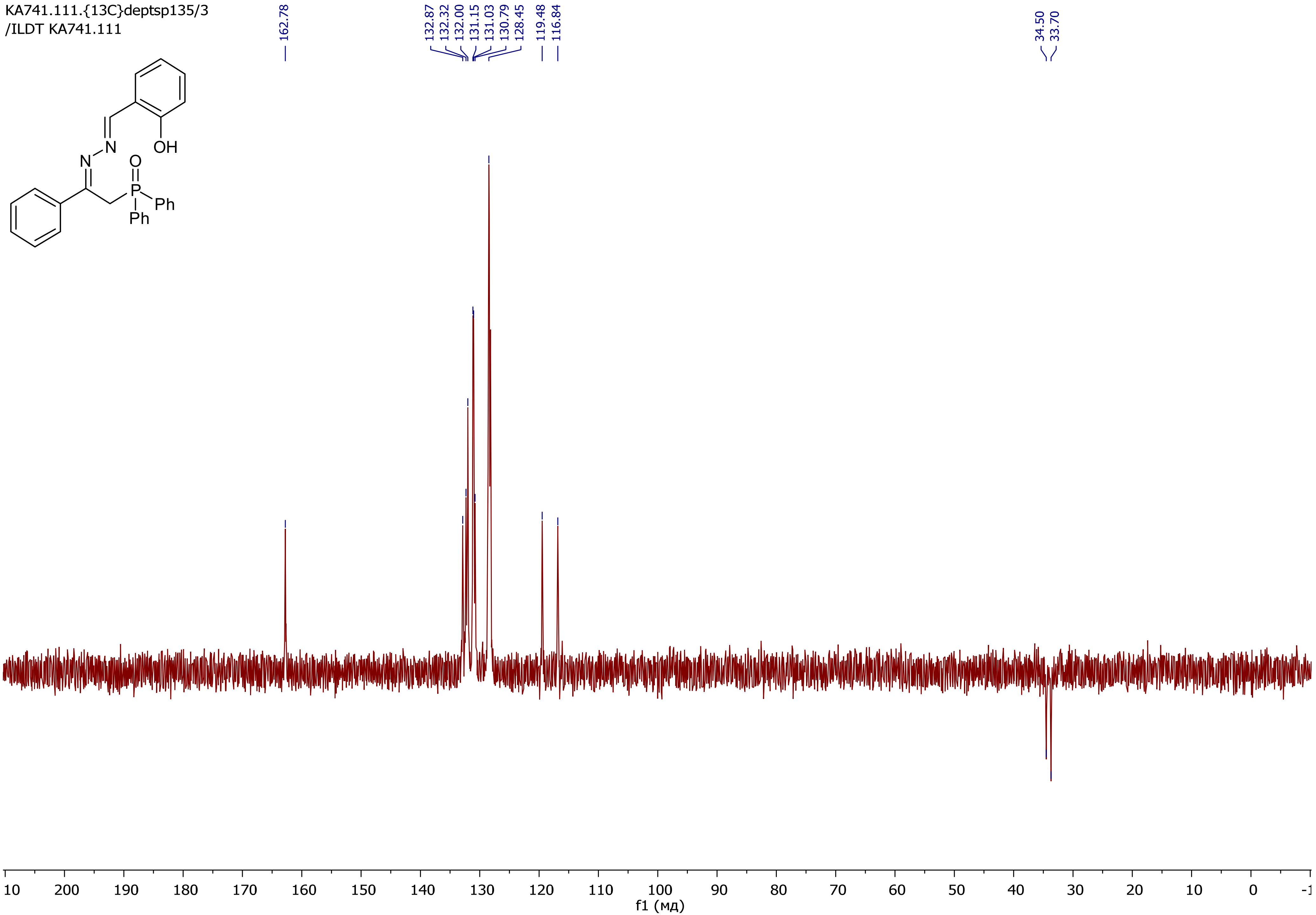
KA741.111.{13C}deptsp135/3
/ILDT KA741.111
N
N
OH
O
P
Ph
Ph
162.78
132.87
132.32
132.00
131.15
131.03
130.79
128.45
119.48
116.84
34.50
33.70
f1 (мд)

KA741.111-1.{31P}INVGATED/31
/ILDT KA741.111-1

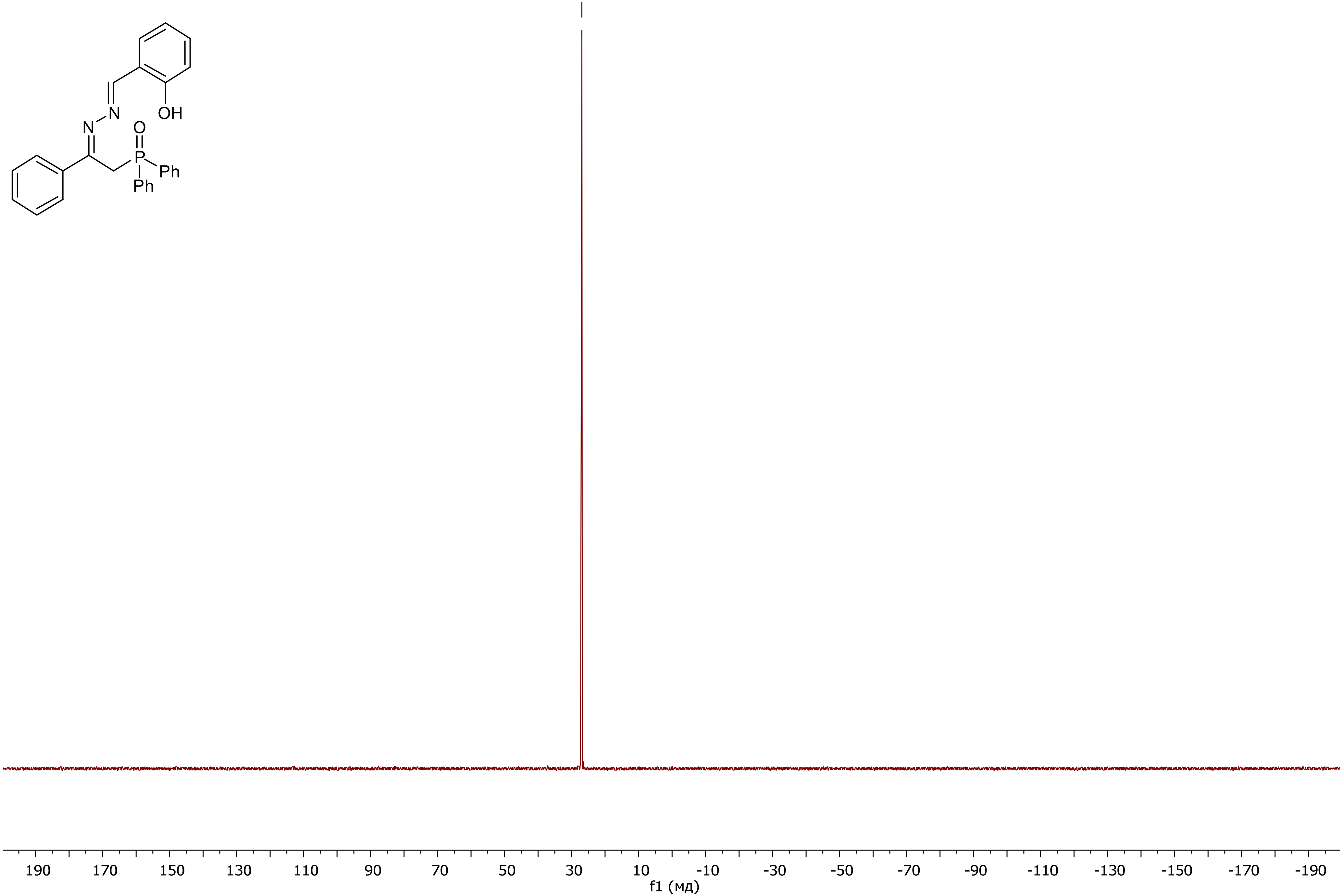

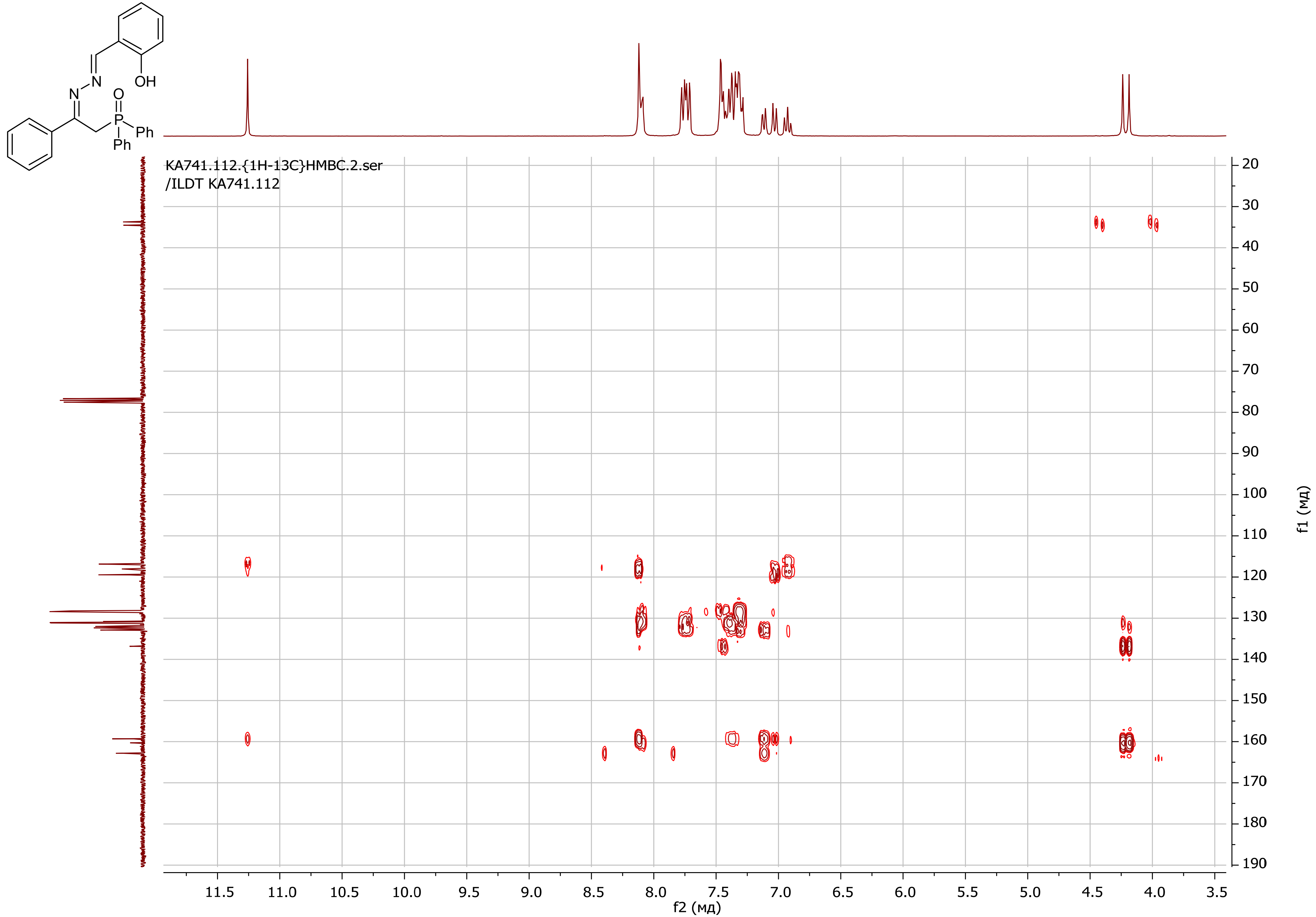
OH
N
N
O
P
Ph
Ph
KA741.112.{1H-13C}HMBC.2.ser
/ILDT KA741.112
f2 (мд)
f1 (мд)

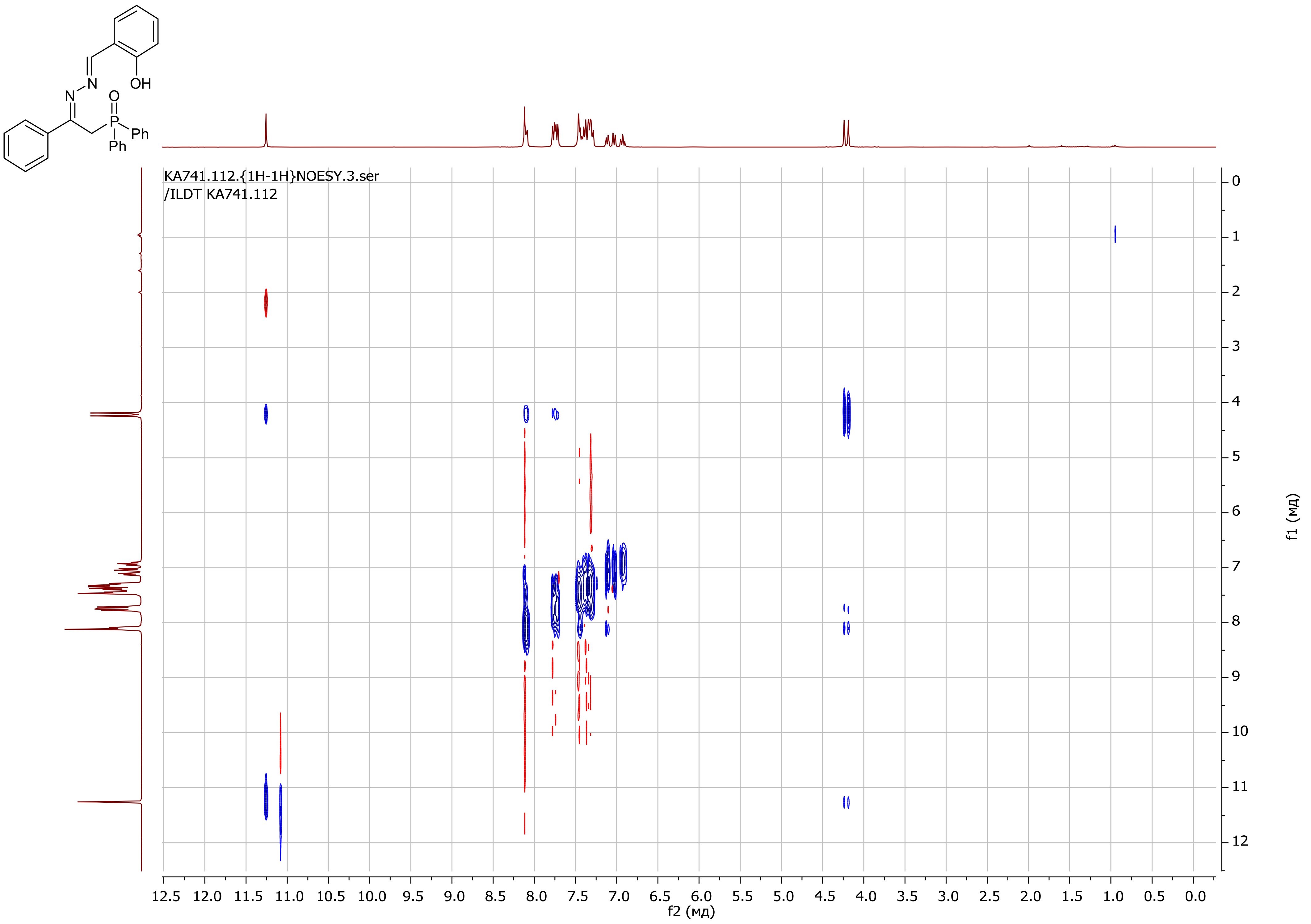
OH
N
N
O
P
Ph
Ph
KA741.112.{1H-1H}NOESY.3.ser
/ILDT KA741.112
f2 (мд)
f1 (мд)
